# Risk Factors Influencing Neurodevelopmental Outcome and Survival in Preterm Infants: A Prospective Cohort Study

**DOI:** 10.3390/children13091214

**Published:** 2026-09-08

**Authors:** Gianluigi Laccetta, Maria Di Chiara, Flavia Gloria, Daniela Regoli, Barbara Caravale, Gianluca Terrin

**Affiliations:** 1 Department of Maternal and Child Health and Urological Sciences, Sapienza University of Rome, 00161 Rome, Italy; 2 Department of Developmental and Social Psychology, Sapienza University of Rome, 00185 Rome, Italy

**Keywords:** preterm infant, very low birth weight, neurodevelopmental outcome, Bayley-III, risk factors, intraventricular haemorrhage, retinopathy of prematurity, neonatal sepsis

## Abstract

**Highlights:**

**What are the main findings?**

- In 146 infants born very preterm and/or with very low birth weight, severe intraventricular haemorrhage was independently associated with language, sepsis with motor and socio-emotional, and male sex with socio-emotional development.
- No nutritional, metabolic, or postnatal growth variable was associated with any domain, and no association survived control for independent growth; no the false discovery rate.

**What are the implications of the main findings?**

- These neonatal morbidities are best read as markers of illness severity that identify infants at higher risk, not as demonstrated therapeutic targets.
- Single-centre cohorts of this size can generate hypotheses but cannot establish them: multicentre external validation and longer follow-up are required.

**Abstract:**

Background/Objectives: Survival of infants born very preterm or with very low birth weight has improved without a comparable reduction in neurodevelopmental morbidity, and the exposures acting during neonatal intensive care are usually studied in isolation. We aimed to identify which prenatal, perinatal and postnatal factors are independently associated, domain by domain, with neurodevelopment at 24 months’ corrected age (CA) and with the composite outcome of neurodevelopment and survival. Methods: Single-centre prospective cohort of 146 infants (135 assessed at 24 months’ CA and 11 who died between day 8 of life and the assessment) with gestational age ≤ 32 weeks and/or birth weight ≤ 1500 g admitted to a level III unit (2018–2022). Neurodevelopment was assessed with the Bayley-III; a score < 85, rather than the conventional <70 being taken as pathological, was used, with the third edition yielding systematically higher scores. Eighty-one candidate exposures were screened; for each domain a primary composite outcome (score < 85 or death) and a secondary survivors-only outcome were analysed by exploratory multivariable logistic regression. Results: Twenty-nine of 164 eligible survivors (17.68%) were lost to follow-up and did not differ appreciably from the analysed cohort (all standardised differences ≤ 0.10). Severe intraventricular haemorrhage was associated with language, sepsis and anaemia requiring transfusion with motor, and male sex and sepsis with socio-emotional development, whereas no covariate was associated with cognition. No nutritional, metabolic or postnatal-growth variable was independently associated with any domain, and no association survived Benjamini–Hochberg control of the false discovery rate (optimism-corrected c-statistics 0.598–0.739). Conclusions: These findings are associations rather than evidence of causality; they are hypothesis-generating and warrant multicentre external validation and longer follow-up.

## 1. Introduction

Over the past decades, advances in neonatal care have improved the survival of preterm infants, particularly extremely preterm and VLBW infants [1]; this survival gain, however, has not been accompanied by a comparable reduction in neurodevelopmental morbidity. A substantial proportion of survivors show, at 2 years’ CA, cerebral palsy or cognitive, language and motor delay, with risk increasing as GA decreases [2,3]. These sequelae frequently persist beyond infancy, affecting learning, autonomy and quality of life, and generate considerable demand for health, rehabilitative and educational services, with a major public-health impact.

Improving prognosis requires the identification of factors that can be recognised during the hospital stay and, where possible, modified. The available evidence, however, has limitations. Narrative syntheses have listed a very large number of prenatal, perinatal and postnatal factors associated with neurodevelopment after preterm birth—low GA and BW, structural brain injury, infection and systemic inflammation, male sex, features of neonatal intensive care itself, nutrition and postnatal growth, and the social and family environment—while noting that most of them derive from cohort or case–control studies in which confounding is difficult to exclude [4]. Two recent systematic reviews restricted to representative geographical or network cohorts have quantified this evidence. At 18 to 36 months’ CA, brain injury (IVH grade III–IV and/or periventricular leukomalacia) carried the highest adjusted odds of moderate-to-severe neurodevelopmental impairment, followed by neonatal seizures and ROP, with bronchopulmonary dysplasia (BPD), postnatal corticosteroids, necrotising enterocolitis (NEC), sepsis, surgery, male sex, absent antenatal corticosteroids, lower GA and lower parental education contributing to a lesser degree, whereas small for gestational age gave inconsistent effects and lower maternal age none [5]. Beyond 36 months and into childhood, brain injury, BPD (particularly when treated with postnatal corticosteroids), lower socio-economic status and, to a lesser extent, male sex were the most consistent predictors of impairment, IQ, cerebral palsy and fine motor and behavioural outcomes [6]. Both reviews had to report their results descriptively, meta-analysis being precluded by the heterogeneity of the definitions of the risk factors and of the outcomes and of the assessment tools; the included studies also span wide enrolment periods over which the definitions of many morbidities and the clinical management of preterm infants changed substantially. Finally, most primary studies examine a single risk factor, or a small group of factors, in isolation, whereas in clinical practice each infant is simultaneously exposed to numerous factors that jointly determine the final outcome; the combined contribution of nutritional, metabolic and postnatal growth variables, in particular, remains poorly explored.

Prospective studies conducted within a recent and narrow time window, with homogeneous management and consistent definitions, and able to evaluate the independent contribution of multiple factors simultaneously while accounting for mortality as a competing risk, are therefore lacking. We hypothesised that a limited number of prenatal, perinatal and postnatal factors are associated with long-term neurodevelopment in a domain-specific manner, and that these factors can be identified only if a wide range of exposures—clinical, nutritional, metabolic and growth-related—is examined jointly within the same cohort, rather than one at a time in separate studies, an approach that loses sight of the complexity of the exposures acting simultaneously on the same infant. The primary objective of this study was accordingly to identify which prenatal, perinatal, and postnatal factors are independently associated, for each Bayley-III domain (cognitive, language, motor, and socio-emotional), with the neurodevelopmental outcome at 24 months’ CA and with the composite outcome of neurodevelopment and long-term survival, in infants with GA ≤ 32 weeks and/or BW ≤ 1500 g.

## 2. Materials and Methods

### 2.1. Study Population

We enrolled infants with GA ≤ 32 weeks and/or BW ≤ 1500 g consecutively admitted to the Neonatal Intensive Care Unit (NICU) of Policlinico Umberto I, Sapienza University of Rome, between 1 January 2018 and 31 December 2022. We excluded outborn infants, infants of mothers with autoimmune disease, and infants with congenital infection, congenital hypothyroidism, congenital anomalies, genetic syndromes, death within the first week of life, and those transferred to or undergoing neurological follow-up at other centres. Written parental consent was required for inclusion.

Infants who died within the first week of life were not included because in these infants the extreme instability of the clinical condition leaves little room for the neonatal intensive care that this study was designed to examine: our purpose was to establish what happens during the neonatal stay, and what of it the neonatologist may influence, in infants who survive the first days and remain exposed to the treatments, morbidities, nutritional strategies and growth trajectories of that stay, most of which are ascertained over days to weeks and are undefined in an infant dying on the first or second day of life. We recognise that this choice conditions the cohort on survival to day 7 and may introduce a survival bias: factors whose effect operates mainly through very early death are, by construction, less visible in our analysis, and the estimates reported here apply to the population of infants alive at day 7. To avoid the analogous and more severe bias that would arise from restricting the analysis to infants surviving to 24 months’ CA, deaths occurring between day 8 of life and the neurodevelopmental assessment were retained and incorporated into the primary composite outcome (Section 2.3).

### 2.2. Data Collected

For each enrolled infant we prospectively collected maternal and pregnancy-related prenatal data, perinatal data up to the first hour of life, and postnatal data, the latter including respiratory support, medications administered during the stay, in-hospital morbidities, fluid-electrolyte and acid-base disturbances, nutritional and body-growth data up to discharge, and PMA at discharge, given the possible influence of these factors on long-term neurodevelopment (Table 1, Table 2, Table 3 and Table 4) [7,8,9,10,11,12,13,14,15,16,17,18,19,20,21,22,23,24,25,26,27,28,29]. The legend to Supplementary Table S1, to which the legend to Table 1, Table 2, Table 3 and Table 4 refers, provides definitions for variables that may be open to interpretation [7,8,9,10,11,12,13,14,15,16,17,18,19,20,21,22,23,24,25,26]. In Table 1, Table 2, Table 3 and Table 4, data are also presented in relation to the study outcomes, defined separately for each neurodevelopmental subscale (cognitive, language, motor, socio-emotional): (1) the primary, composite outcome, consisting of suboptimal neurodevelopment at 24 months’ CA or death between day 8 of life and 24 months’ CA; and (2) the secondary outcome, consisting of neurodevelopment at 24 months’ CA among infants who survived to assessment. For both outcomes, infants classified as “cases” had died (composite outcome only) or had a suboptimal score, whereas “controls” were infants with normal development on the considered scale.

### 2.3. Neurodevelopmental Outcome and Study Design

Long-term neurodevelopment was assessed with the Bayley Scales of Infant and Toddler Development, third edition (Bayley-III); specifically, we evaluated the influence of each factor on each neurodevelopmental subscale (cognitive, language, motor, socio-emotional). Scores < 85 on each Bayley-III scale were considered pathological, and scores ≥ 85 were considered normal. Assessment was performed at 24 months’ CA by a single, independent, and experienced examiner (a child neuropsychiatrist) within the follow-up programme provided at our centre for infants at neurodevelopmental risk.

The threshold of 85 (−1 standard deviation) rather than the conventional 70 (−2 standard deviations) was adopted deliberately. Although the Bayley-III provides separate cognitive, language, motor and socio-emotional scales, it yields systematically higher scores than the Bayley-II and consequently classifies fewer children as impaired [30,31,32,33]. In extremely preterm children assessed with both editions at the same session, the combined Bayley-III cognitive/language score was on average 7 points higher than the Bayley-II mental developmental index (MDI), the discrepancy widening as ability decreased; a Bayley-III cut-off of <70 identified only 58% of the children with an MDI < 70, whereas a cut-off of <80 achieved 89% sensitivity and 99% specificity [31]. In term-born infants assessed after neonatal encephalopathy, the median Bayley-III cognitive composite was 7 points and the median motor composite 18 points higher than the corresponding Bayley-II indices, and Bayley-III scores < 85 classified essentially the same infants as severely delayed as Bayley-II scores < 70 [32,33]. Conversion algorithms derived in mixed term and preterm samples point in the same direction, with an MDI of 70 corresponding to a calculated Bayley-III cognitive score of about 93 [34]. The practical consequence is visible in population studies: in the Swedish EXPRESS cohort the prevalence of moderate or severe impairment was two to three times lower when Bayley-III scores were referred to the published norms than when they were referred to a contemporaneous term-born control group [35]. Using the conventional <70 threshold in a cohort such as ours would therefore have classified as normal a substantial number of infants with clinically meaningful delay, and would have made our results incomparable with the historical literature based on the Bayley-II, whose predictive value for later cognitive functioning has been quantified meta-analytically [36]. We accordingly adopted <85, which is also the threshold at which “any delay” is conventionally defined in recent systematic reviews of neurodevelopment at 2 years [5]. The implication of this choice, which must be borne in mind when comparing our figures with those of other cohorts, is that the outcome analysed here identifies infants with mild as well as with moderate-to-severe difficulties, so that the proportions of adverse outcomes we report are necessarily higher than those of studies adopting <70.

The fourth edition of the Bayley scales was not used. It was published in 2019 [37], immediately before the neurodevelopmental assessment of the infants born in 2018 began, at a time when we had neither normative experience nor sufficient practical familiarity with the new edition; the assessments were therefore started with the Bayley-III and continued with the same instrument until the end of the study, so that all infants were evaluated with one scale and their scores remain mutually comparable.

For each of the four subscales, we defined two separately analysed outcomes. The primary outcome was composite and consisted of a pathological score (<85) on the corresponding Bayley-III subscale at 24 months’ CA or death between day 8 of life and 24 months’ CA, compared with normal development (score ≥ 85); “cases” included both deceased infants and survivors with a pathological score, whereas “controls” were survivors with a normal score. A composite outcome incorporating mortality was chosen as the primary outcome because death represents a competing risk closely associated with the same prognostic factors under investigation: analysing survivors only would condition the sample on survival and introduce a selection bias that could underestimate—or even reverse—the effect of factors most strongly associated with mortality. The secondary outcome, configured as a sensitivity analysis, consisted of neurodevelopment at 24 months’ CA (pathological vs. normal) among survivors only, and aimed to isolate the correlates of neurodevelopment among survivors, to be interpreted with caution given the conditioning on survival. Comparing the two outcomes allows discrimination between factors acting mainly through mortality (significant in the composite analysis only), factors with a robust effect on neurodevelopment itself (significant in both analyses), and factors relevant among survivors only. For the composite outcome to fulfil this function, the infants who died must actually contribute to the estimation, and this constrained the covariates that could be entered in the primary models, as described in Section 2.5.

### 2.4. Bayley-III Administration

The Bayley-III was administered in Italian or in the patient’s preferred language, given the examiner’s ability to also speak other languages (English, French and Spanish).

### 2.5. Statistical Analysis

Continuous variables are reported as mean ± standard deviation when normally distributed and as median (interquartile range) otherwise; categorical variables are reported as counts and percentages. The normality of continuous variables was assessed with the Shapiro–Wilk test and the homogeneity of variance with Levene’s test. Associations between each candidate variable and each study outcome were first examined at the univariate level: categorical variables were compared using the chi-square test, or Fisher’s exact test when expected cell counts were below five, whereas continuous variables were compared using the independent-samples *t*-test when normally distributed and the Mann–Whitney U test when non-normally distributed. All tests were two-sided, and a *p*-value < 0.05 was considered statistically significant.

Candidate covariates for the multivariable models were identified in two steps. First, the whole set of variables listed in Supplementary Tables S1–S4 was screened at the univariate level. Second, the variables associated with the outcome at *p* < 0.05 were reviewed on clinical and causal grounds before being entered: for each domain we drew a directed acyclic graph in which the exposures collected in this study were connected according to the pathophysiology of preterm birth (for example gestational age → invasive mechanical ventilation → bronchopulmonary dysplasia → postnatal corticosteroids → postmenstrual age at discharge, or chorioamnionitis → early-onset sepsis → duration of antibiotic therapy), and used it to distinguish confounders, which were retained, from variables lying on the pathway between another candidate and the outcome, or constituting near-duplicate measurements of the same construct, which were not entered [38,39]. The edges of these graphs, whose core structure is common to the four domains and which differ only in the exposures reaching the univariate threshold for each domain, are reported together in Supplementary Table S5. The intention was therefore not to reduce each model to the few strongest associations but to remove redundancy: to the best of our judgement, no clinically important confounder available in the database was excluded, whereas variables carrying essentially the same information as a retained covariate—for instance, the several nutritional variables describing the composition of the same feeds, whose relative proportions are fixed by the product—were dropped. We acknowledge the recognised limitation of univariate screening, which may exclude a variable that acts as a confounder without being marginally associated with the outcome [40]; the clinical and causal review of the second step was applied precisely in order to mitigate it, and the univariate results are reported for every variable analysed, so that the reader may judge what was and what was not carried forward.

Gestational age and birth weight were both allowed to enter the models as candidates, since they capture distinct constructs—maturity at birth and intrauterine growth—whose relation is neither constant nor deterministic, a higher gestational age not necessarily corresponding to a higher birth weight and vice versa, as everyday neonatal practice illustrates; their correlation in this cohort (r = 0.620) leaves the greater part of the variance of each unshared and would not by itself have precluded simultaneous adjustment. In the end, birth weight was not retained in any of the twelve final models, so that no model adjusted for the two variables at the same time (Supplementary Table S6). Birth weight was not retained because in every domain-specific graph it lay downstream of gestational age and of the prenatal determinants of foetal growth that were themselves available as candidates (intrauterine growth restriction, hypertensive disorders of pregnancy, abnormal umbilical Doppler flow), so that it added no control of confounding that gestational age together with those variables did not already provide, while its correlation with gestational age would have inflated the variance of both coefficients.

The number of covariates entered was deliberately kept large relative to the number of events, and this choice requires justification. The purpose of the study was not to derive a parsimonious prediction rule but to describe the joint action of a broad set of prenatal, perinatal and postnatal exposures, most of which are strongly interrelated: a model containing three or four predictors would have produced apparently precise estimates that in fact absorb the effect of the omitted, correlated exposures, thereby exchanging the risk of overfitting for the less visible and, for an aetiological question, more serious risk of confounding by variables that had actually been measured. The conventional requirement of ten events per variable, moreover, is a rule of thumb derived from a small simulation study [41] whose generality has since been questioned: with the effect sizes and covariate distributions typical of observational cohorts, five to nine events per variable are frequently adequate, and no single threshold separates acceptable from unacceptable models, the required sample size depending on the number of candidate predictors, the outcome proportion and the expected explained variation [42,43]. Rather than reduce the covariate set in order to satisfy this rule, we quantified the resulting optimism directly: events per variable are reported for every model, the c-statistic was corrected for optimism by bootstrapping, the calibration slope was estimated as an explicit measure of the shrinkage that the coefficients require, collinearity was examined by several diagnostics and the false discovery rate was controlled (Supplementary Tables S6–S9). The analyses are accordingly presented as exploratory and the coefficients as upper bounds of the underlying associations requiring shrinkage, rather than as transportable effect sizes; the models are not proposed for individual risk prediction.

To assess the potential for selection bias, the infants lost to follow-up were compared with the infants included in the analysis with respect to the main prenatal, perinatal and postnatal characteristics, in two comparisons: against the 135 infants assessed at 24 months’ CA and against the whole analysed cohort of 146 infants. Because the comparison involves 29 infants and is therefore underpowered—so that a non-significant *p*-value could not be taken as evidence of comparability—the primary metric was the standardised difference, which does not depend on sample size and whose absolute value is conventionally taken to indicate negligible imbalance when below 0.10 and acceptable imbalance when below 0.20 [44]; conventional significance tests are reported alongside it for completeness, with their limited power explicitly acknowledged.

For readability, Table 1, Table 2, Table 3 and Table 4 report the variables that reached, or approached, the conventional significance threshold (*p* < 0.10) in at least one of the two comparisons; the complete tables, containing every variable analysed together with the full legend and list of abbreviations, are provided as Supplementary Tables S1–S4, and the analysis variables are additionally listed by category (prenatal, perinatal, postnatal clinical, nutritional, growth, metabolic, acid-base and electrolyte) in Supplementary Table S10. Throughout the manuscript, *p*-values between 0.05 and 0.10 are reported for completeness and are not described as trends towards significance, and statistical significance is kept distinct from clinical relevance in the interpretation of the results.

For each of the eight outcomes (the four Bayley-III domains, each analysed both as the primary composite outcome and as the secondary survivors-only outcome), a multivariable binary logistic regression model was fitted; because the composite outcome was modelled in two specifications, the primary one and the sensitivity one described below, twelve models were fitted in all. Candidate covariates were those significant at univariate analysis, further reviewed on clinical and causal grounds as described above in order to remove redundancy rather than to minimise the number of predictors; the absence of relevant collinearity among the selected covariates was confirmed by variance inflation factors below five. For the four models of the primary composite outcome, a further restriction was applied: only covariates that are defined in every infant of the cohort were entered, so that the 11 infants who died contributed to the estimation and the composite outcome performed the function for which it was chosen. The models were not prespecified: the analysis plan was drawn up after data collection had been completed, and this is acknowledged among the limitations. In each model, the dependent variable was coded so that the modelled event was normal neurodevelopment; accordingly, an Exp(B) (odds ratio) greater than 1 indicates higher odds of a normal outcome and a value below 1 indicates higher odds of the adverse or composite outcome. Effect estimates are reported as Exp(B) with 95% confidence intervals. Model calibration was assessed with the Hosmer–Lemeshow goodness-of-fit test and the explained variation with the Nagelkerke R-squared.

For each model, we report the number of events per variable (EPV), calculated as the number of participants in the less frequent outcome category divided by the number of estimated covariate parameters, the conventional threshold being 10 events per variable. Because the models were fitted by listwise deletion of incomplete cases, events per variable were computed on the analytic sample of each model, that is, on the participants with complete data for all of the covariates entered, and not on the whole cohort of 146 infants; the analytic sample size and the resulting value are reported for every model in Supplementary Table S7. Internal validation was performed by non-parametric bootstrapping: 1000 samples of the same size as the analysed sample were drawn with replacement, and the model, with its covariate set held fixed, was refitted in each of them and applied to the original sample; optimism was estimated as the mean difference in the c-statistic between the bootstrap sample and the original sample, and the optimism-corrected c-statistic was obtained by subtracting this quantity from the apparent value. The bootstrap calibration slope, which quantifies the shrinkage required by the regression coefficients, was estimated as the mean slope of the logistic regression of the observed outcome on the linear predictor derived from each bootstrap model. Performance measures were computed on the participants with complete data for all covariates entered in the corresponding model (N = 133–135). Listwise deletion has a further and more important consequence for the composite models, and it determined the way the primary analysis was specified. The postmenstrual age at hospital discharge is undefined in every infant who died before discharge, and the retinopathy status and the growth Z-scores at 36 weeks’ postmenstrual age are undefined in the infants who died before those time points; a composite model containing any of these covariates is therefore fitted, after listwise deletion, on survivors only or almost only, and reintroduces precisely the conditioning on survival that the composite outcome was designed to avoid. The four models of the primary composite outcome reported in the main text (with the complete estimates in Supplementary Table S11) were accordingly restricted to the covariates that are defined in every infant, so that all 146 infants, including the 11 deaths, contribute to the estimation. The corresponding models retaining the complete covariate set, which achieve a more complete adjustment but which listwise deletion restricts to survivors, are reported as a sensitivity analysis in Supplementary Tables S12–S15. Because of the small number of events per variable and of the near-complete separation observed for some covariates, the primary composite models were additionally estimated by Firth’s penalised likelihood. Resamples in which the maximum-likelihood estimate failed to converge because of near-complete separation were excluded from the primary estimate (0.1% to 27.5% of resamples, depending on the model); a sensitivity analysis including them is reported in the footnote to Supplementary Table S7. Because the covariate set was held fixed within the bootstrap, this procedure does not account for the optimism introduced by the preceding univariate selection step, so the optimism reported here is a lower bound, and the optimism-corrected c-statistic and the calibration slope are correspondingly upper bounds of the internal validity of the models, that is, still optimistic.

Discrimination was displayed as receiver operating characteristic curves and calibration as flexible calibration plots, obtained by lowess smoothing of the observed outcome on the predicted probability and complemented by the observed proportions within quintiles of predicted risk, for the four primary composite models (Supplementary Figures S1 and S2) and for the eight models of Supplementary Tables S12–S19 (Supplementary Figures S3 and S4). The assumption of a linear effect of each continuous covariate on the logit was examined by replacing the linear term with a restricted cubic spline with three knots placed at the 10th, 50th and 90th centiles of the covariate and comparing the two models with a likelihood-ratio test on one degree of freedom; covariates with fewer than six distinct values, or whose 10th, 50th and 90th centiles coincided, were not amenable to this test. The test was applied to the covariate terms of the models of Supplementary Tables S12–S19; because the covariates of the four primary composite models are a subset of those of the corresponding models of Supplementary Tables S12–S15, every continuous term entering the primary models was covered by it. With events per variable ranging from 1.7 to 17.0 in the models concerned, and below six in six of the eight, this test has low power, so that the absence of evidence of a departure from linearity must not be read as evidence of linearity. For the terms in which this test reached the nominal threshold, the corresponding models were refitted with the covariate entered as a restricted cubic spline, all the other covariates being unchanged, and the covariates significant at the nominal level were compared with those of the linear models (Supplementary Table S20). Although the multivariable analyses were regarded as exploratory, the false discovery rate was additionally controlled with the Benjamini–Hochberg procedure, applied separately within each family of univariate comparisons, a family being defined as all variables tested against one outcome in one of the two comparisons, and within the covariates of each multivariable model as well as across all 169 covariate tests of the twelve models; adjusted values are reported as q values in Supplementary Tables S8 and S9. Collinearity was examined beyond the variance inflation factor by inspecting tolerances, the largest absolute pairwise correlation among the covariates of each model and the condition indices of the column-centred and column-scaled design matrix, bearing in mind that the conventional threshold of 30 was derived for the scaled but uncentred matrix, so that these indices serve as a comparison between models rather than as a test against that threshold (Supplementary Table S6). The extent of missing data was quantified for every analysis variable, together with its distribution with respect to survival and the reason for each missing value (Supplementary Table S21).

Given the number of models and covariates examined, the analyses were regarded as exploratory and hypothesis-generating, and the primary inference was not conditioned on a formal correction for multiple comparisons, the false discovery rate being controlled as a sensitivity analysis as described above; no a priori sample-size calculation was performed. Variables with incomplete ascertainment were analysed on available cases, and the corresponding denominators are reported in Table 1, Table 2, Table 3 and Table 4. Missingness was entirely predictable from death, which is itself observed, but the problem it raises is not one of missing data in the usual sense: for the great majority of the missing values the quantity is not unobserved but undefined, the time at which it would have been measured lying beyond the time of death, so that this is a situation of truncation by death rather than of missingness. Multiple imputation was therefore not used, since imputing a postmenstrual age at discharge, or a growth Z-score at 36 weeks’ postmenstrual age, for an infant who died before that time point would generate values without a referent rather than recover unobserved ones. Seven of the 149 missing values are an exception, being quantities that existed but were not recorded (the day of initiation of enteral feeding in one infant and the day of recovery of birth weight in six infants who died); given their number, they were also analysed on available cases. Bootstrap analyses were performed in Python 3.11 with the statsmodels 0.14 library; all remaining analyses were performed with SPSS version 27 (IBM Corp., Armonk, NY, USA).

## 3. Results

### 3.1. Participant Flow

**Figure 1:**
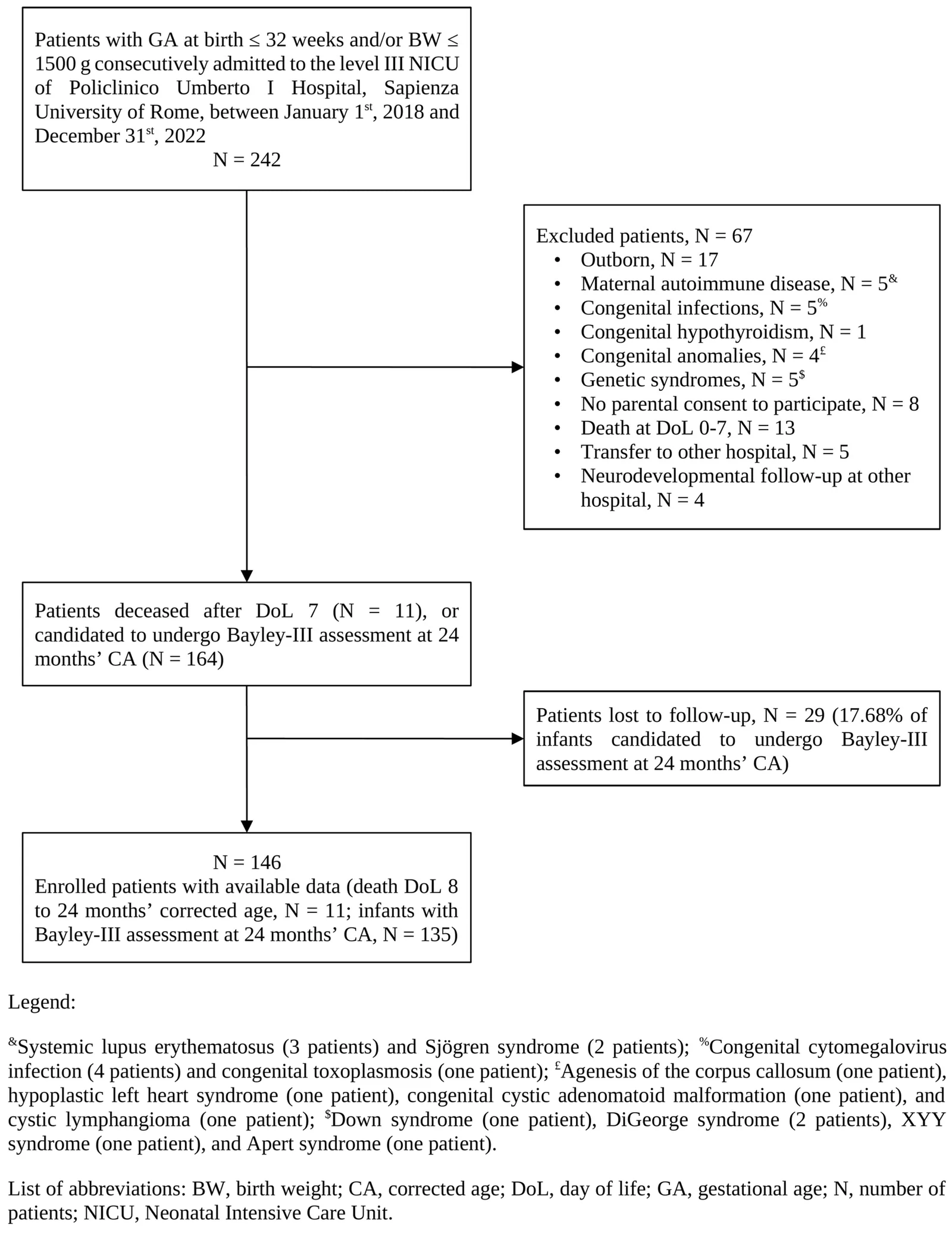
Flow chart providing a comprehensive overview of enrolment, exclusions, and follow-up of the study sample. Detailed exclusion categories are reported in the figure legend. See references [29,30,31,32,33].

Between 1 January 2018 and 31 December 2022, 242 infants with GA ≤ 32 weeks and/or BW ≤ 1500 g were consecutively admitted to the level III NICU of Policlinico Umberto I (Sapienza University of Rome). Sixty-seven infants were excluded for the following reasons: outborn birth (n = 17), maternal autoimmune disease (n = 5), congenital infection (n = 5), congenital hypothyroidism (n = 1), congenital anomalies (n = 4), genetic syndromes (n = 5), lack of parental consent (n = 8), death within the first 7 days of life (n = 13), transfer to another hospital (n = 5), and neurodevelopmental follow-up at another centre (n = 4). Of the 175 remaining infants, 11 died after day 7 of life, and 164 were eligible for Bayley-III assessment at 24 months’ CA; of the latter, 29 (17.68%) were lost to follow-up. The final analysis therefore included 146 infants: 135 survivors with neurodevelopmental assessment at 24 months’ CA and 11 who died between day 8 of life and 24 months’ CA (Figure 1) [45,46,47,48,49].

The reasons for loss to follow-up were untraceability of the family (12/29, 41.38%), transfer of the neurodevelopmental follow-up to another centre after discharge (10/29, 34.48%), parental refusal of the assessment (4/29, 13.79%), and change in residence (3/29, 10.34%).

### 3.2. Baseline Characteristics

The prenatal (maternal and pregnancy-related), perinatal, and postnatal characteristics of the population, reported in relation to each outcome and to each of the two comparisons (composite analysis and survivors-only analysis), are detailed in Table 1, Table 2, Table 3 and Table 4. Overall, of the 146 included infants, 77 (52.74%) were male and 128 (87.67%) were of Caucasian ethnicity; 135 (92.47%) survived to assessment at 24 months’ CA and 11 (7.53%) died between day 8 of life and 24 months’ CA. No infant in the cohort had culture-proven early-onset sepsis; the covariate designated early- and/or late-onset sepsis therefore corresponds in practice to culture-proven late-onset sepsis, which occurred in 22 of the 146 infants (15.07%), and is to be read as such throughout. The 11 infants who died differed markedly from the survivors in the severity of their course: none of them had received antenatal corticosteroids at any dose (0/11 vs. 115/135, 85.19%), all had required invasive mechanical ventilation (11/11 vs. 37/135, 27.41%), 10 of 11 had IVH grade III–IV (vs. 9/135, 6.67%) and 10 of 11 culture-proven late-onset sepsis (vs. 12/135, 8.89%).

### 3.3. Infants Lost to Follow-Up

The 29 infants lost to follow-up were compared with the 135 infants assessed at 24 months’ CA and with the 146 infants included in the analysis (Table 5). No characteristic differed appreciably between the groups: all standardised differences were ≤0.16 against the infants assessed at 24 months’ CA and ≤0.10 against the whole analysed cohort, and no comparison approached statistical significance (all *p* ≥ 0.44). The largest imbalances, both within the range conventionally regarded as negligible to acceptable, concerned culture-proven late-onset sepsis (13.79% vs. 8.89%; standardised difference 0.16) and IVH grade III–IV (10.34% vs. 6.67%; standardised difference 0.13), both marginally more frequent among the infants lost to follow-up than among those assessed. Gestational age, birth weight, head circumference, 5 min Apgar score, sex, twin birth, maternal ethnicity and educational level, antenatal corticosteroid exposure, mode of delivery, chorioamnionitis, invasive mechanical ventilation, ROP, BPD, NEC, treated patent ductus arteriosus and PMA at discharge were almost identical across the groups, as was the year of birth (standardised difference −0.07 against the infants assessed at 24 months’ CA and −0.10 against the whole cohort). These findings do not exclude selection bias, which could still arise from characteristics that were not measured, but they make it unlikely that the analysed cohort differs systematically from the eligible population with respect to the exposures examined in this study.

### 3.4. Primary Outcome

**Figure 2:**
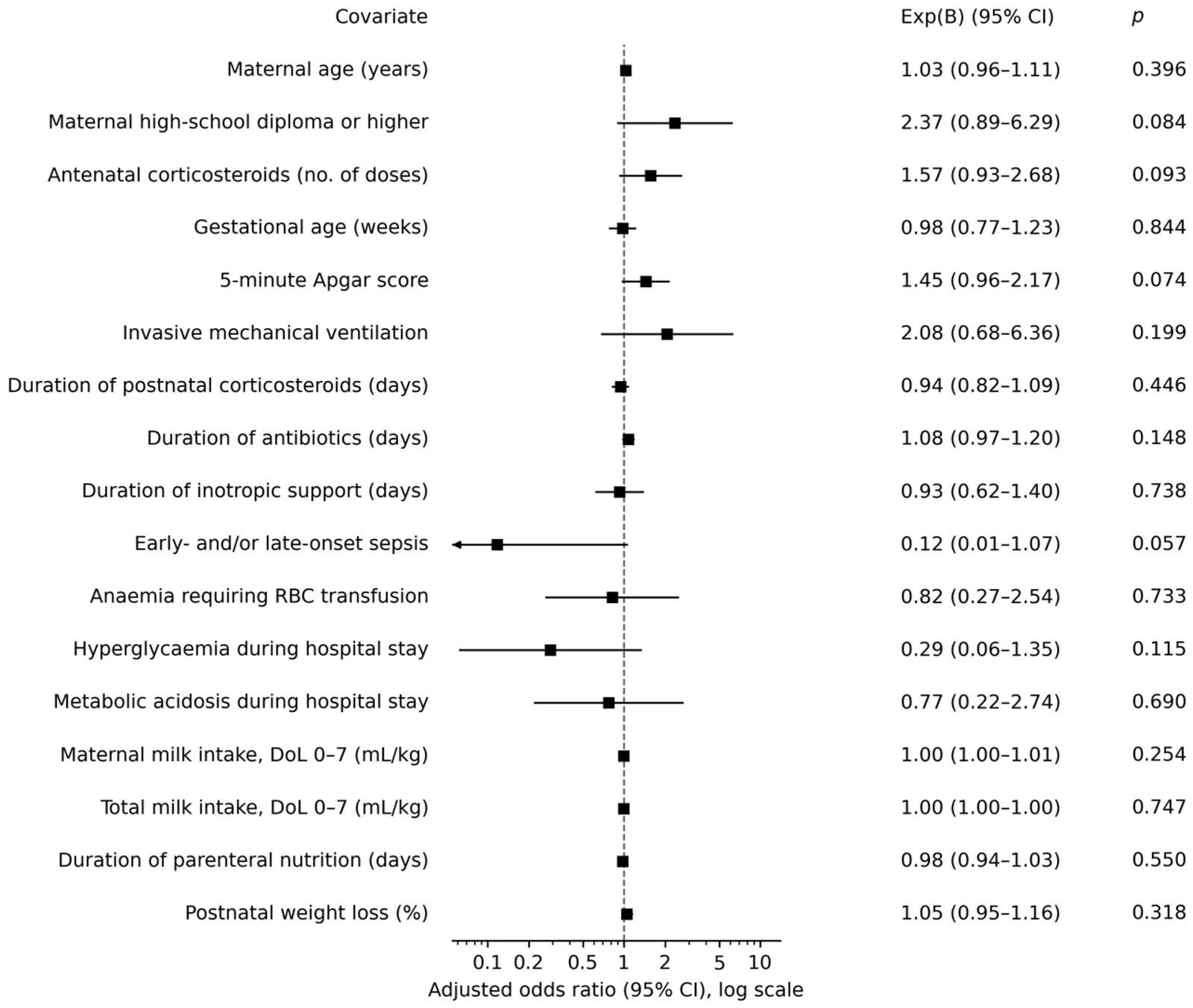
Primary multivariable analysis of the composite outcome for the cognitive domain, fitted on all 146 infants. Adjusted odds ratios [Exp(B)] with 95% confidence intervals from a binary logistic regression model restricted to the covariates that are defined in every infant of the cohort, so that the 11 infants who died also contribute to the estimation; the modelled event was normal development (Exp(B) > 1, higher odds of a normal outcome; <1, higher odds of the adverse/composite outcome). Dashed line, odds ratio = 1; arrowheads, confidence limits beyond the axis range; statistical significance was set at *p* < 0.05. No covariate remained significant after Benjamini–Hochberg adjustment (Supplementary Table S9). Cases/controls 64/82; 17 covariates; events per variable 3.8; Nagelkerke R^2^ = 0.367; Hosmer–Lemeshow *p* = 0.201; apparent c = 0.798, optimism-corrected c = 0.703, bootstrap calibration slope 0.55. The complete model, with Firth-penalised estimates, is reported in Supplementary Table S11. List of abbreviations: CI, confidence interval; DoL, days of life; RBC, red blood cell.

**Figure 3:**
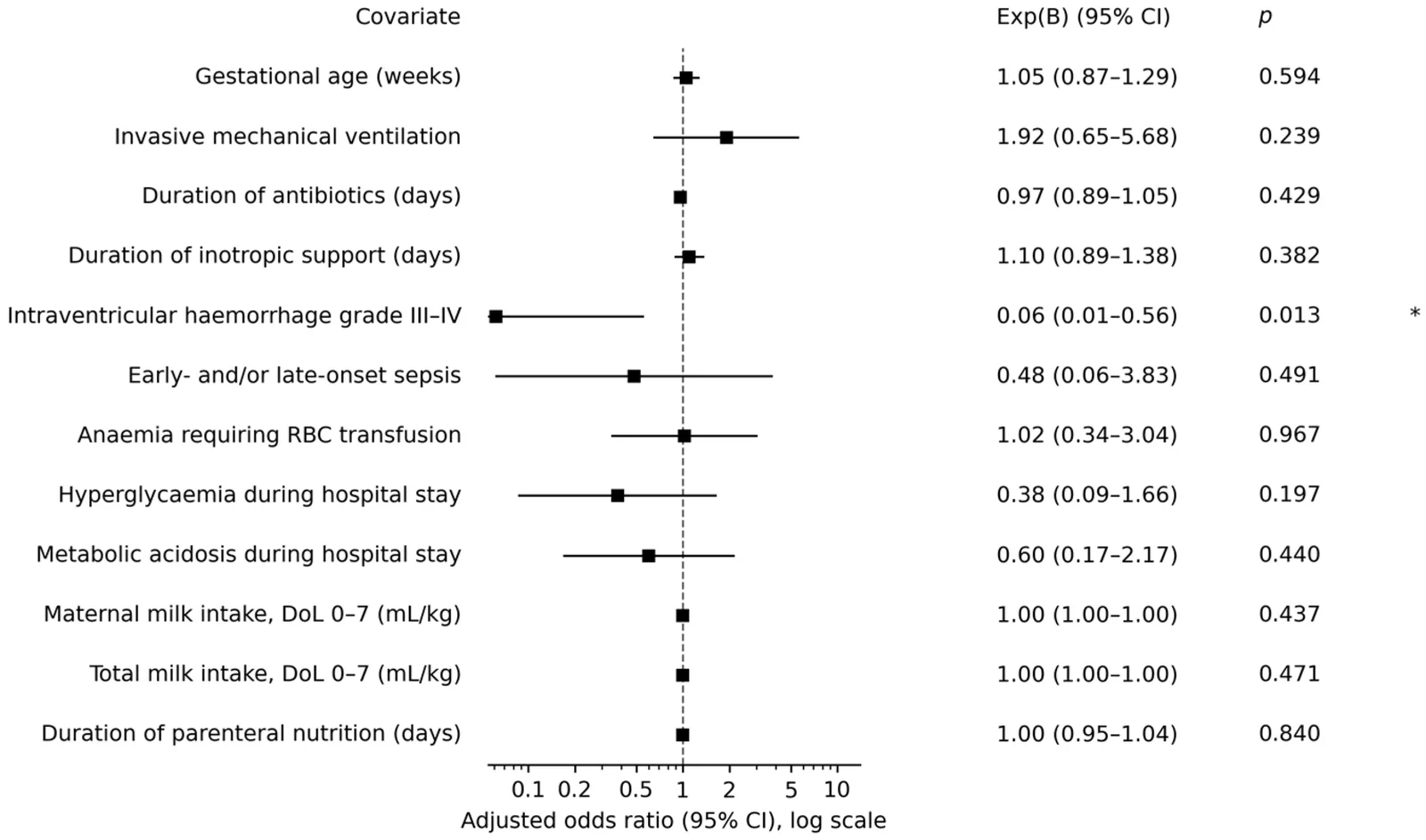
Primary multivariable analysis of the composite outcome for the language domain, fitted on all 146 infants. * *p* < 0.05. Details as in Figure 2. Cases/controls 78/68; 12 covariates; events per variable 5.7; Nagelkerke R^2^ = 0.284; Hosmer–Lemeshow *p* = 0.883; apparent c = 0.768, optimism-corrected c = 0.696, bootstrap calibration slope 0.60. List of abbreviations: CI, confidence interval; DoL, days of life; RBC, red blood cell.

**Figure 4:**
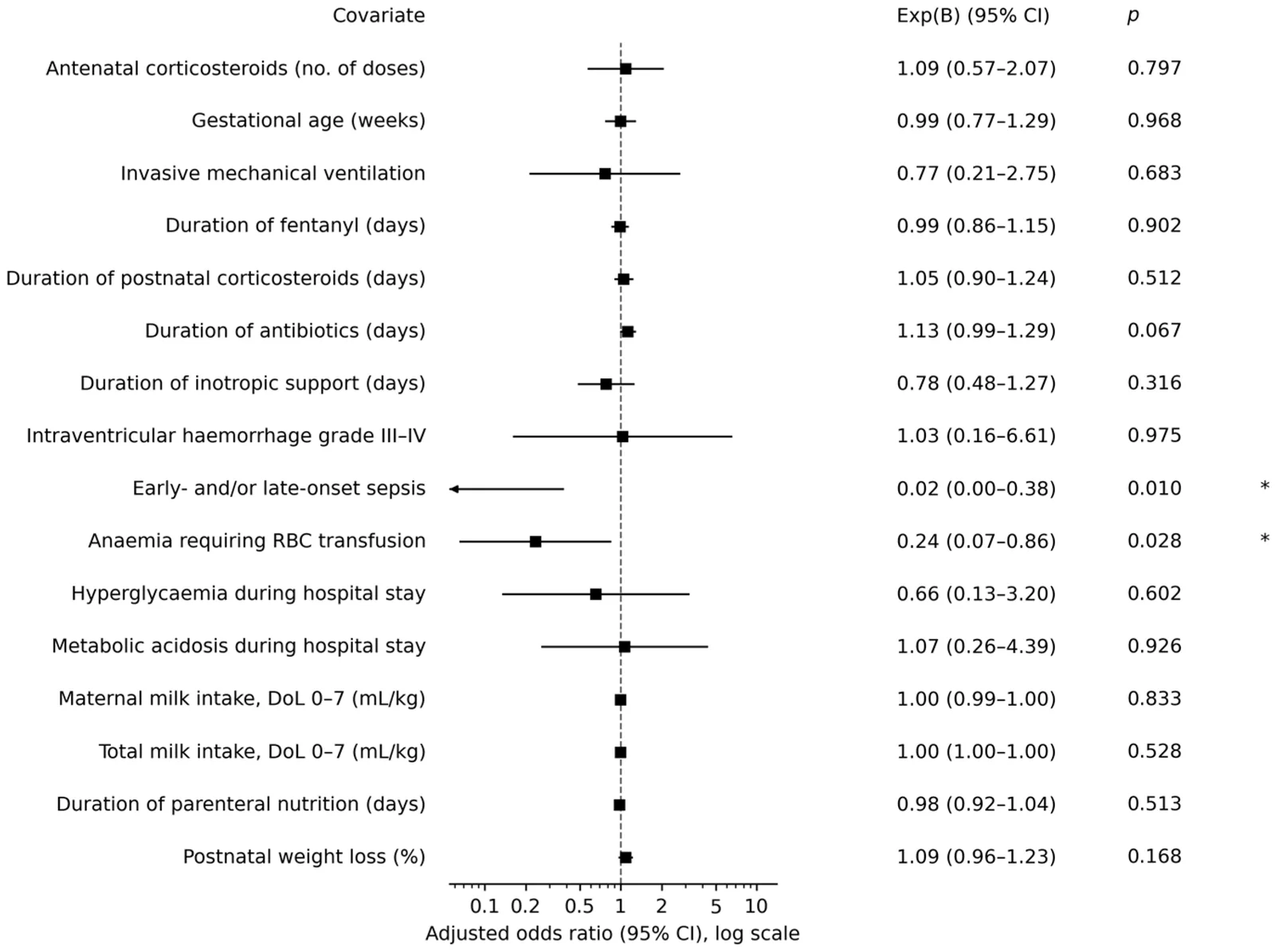
Primary multivariable analysis of the composite outcome for the motor domain, fitted on all 146 infants. * *p* < 0.05. Details as in Figure 2. Cases/controls 45/101; 16 covariates; events per variable 2.8; Nagelkerke R^2^ = 0.507; Hosmer–Lemeshow *p* = 0.085; apparent c = 0.825, optimism-corrected c = 0.739, bootstrap calibration slope 0.57. List of abbreviations: CI, confidence interval; DoL, days of life; RBC, red blood cell.

**Figure 5:**
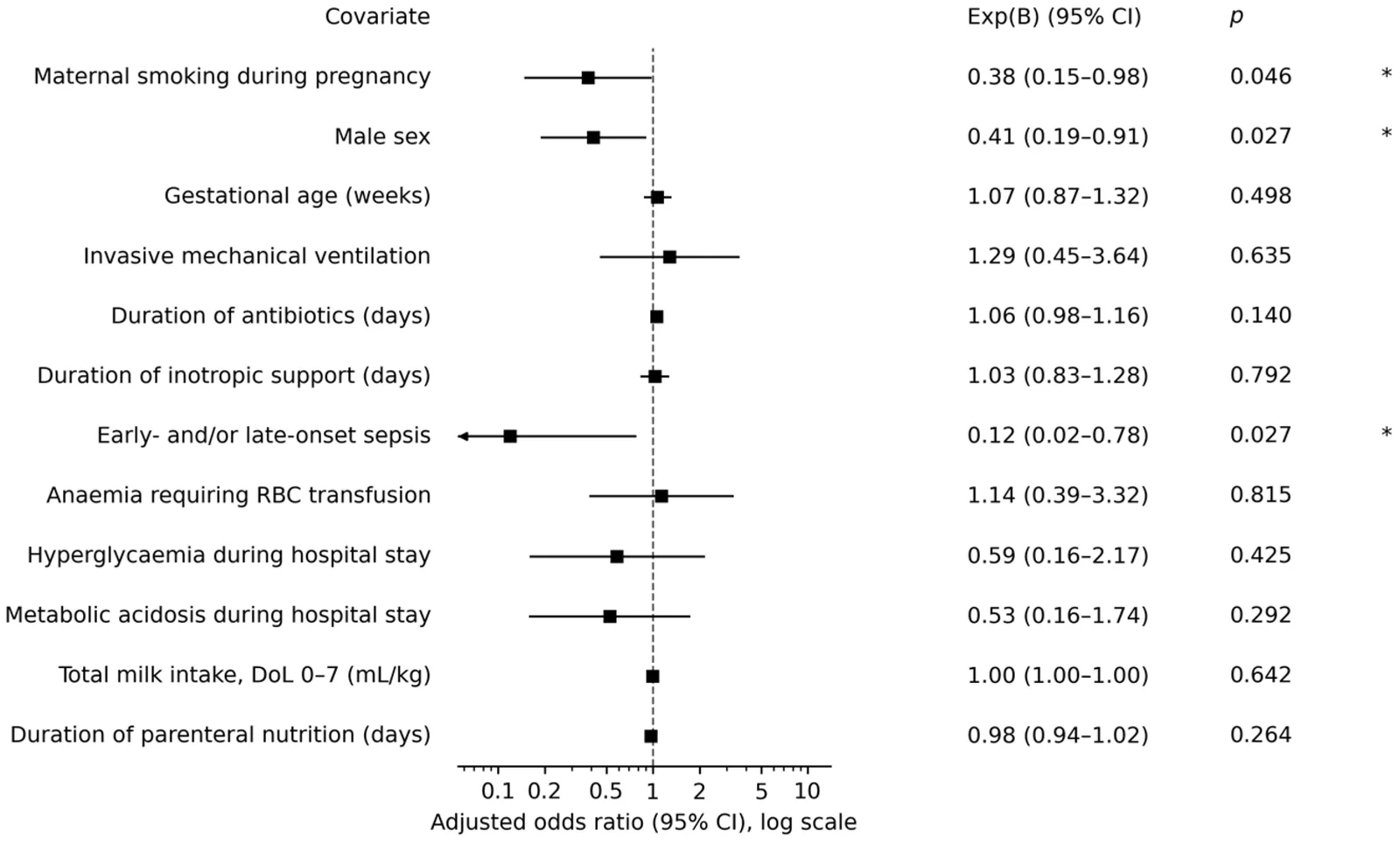
Primary multivariable analysis of the composite outcome for the socio-emotional domain, fitted on all 146 infants. * *p* < 0.05. Details as in Figure 2. Of the three covariates marked as significant, maternal smoking during pregnancy did not remain so under Firth’s penalised likelihood (*p* = 0.061). Cases/controls 62/84; 12 covariates; events per variable 5.2; Nagelkerke R^2^ = 0.267; Hosmer–Lemeshow *p* = 0.345; apparent c = 0.753, optimism-corrected c = 0.672, bootstrap calibration slope 0.56. List of abbreviations: CI, confidence interval; DoL, day of life; RBC, red blood cell.

Considering deceased infants as cases, a Bayley-III score in the normal range (≥85, the threshold adopted here in place of the conventional ≥70; Section 2.3) was recorded in 82/146 (56.16%) infants for the cognitive scale, 68/146 (46.58%) for language, 101/146 (69.18%) for motor and 84/146 (57.53%) for socio-emotional. In the primary multivariable analysis, in which all 146 infants including the 11 who died contributed to the estimation, no covariate was associated with the cognitive outcome (Figure 2); IVH grade III–IV was associated with the language outcome [Exp(B) = 0.062; 95% CI 0.007–0.562; *p* = 0.013] (Figure 3); early- and/or late-onset sepsis [Exp(B) = 0.018; 95% CI 0.001–0.382; *p* = 0.010] and anaemia requiring one or more red blood cell transfusions [Exp(B) = 0.236; 95% CI 0.065–0.856; *p* = 0.028] with the motor outcome (Figure 4); and male sex [Exp(B) = 0.414; 95% CI 0.189–0.906; *p* = 0.027], early- and/or late-onset sepsis [Exp(B) = 0.120; 95% CI 0.019–0.783; *p* = 0.027] and maternal smoking during pregnancy [Exp(B) = 0.382; 95% CI 0.148–0.983; *p* = 0.046] with the socio-emotional outcome (Figure 5). Estimates obtained by Firth’s penalised likelihood were concordant in direction and slightly attenuated in magnitude, and remained significant at the nominal level for every covariate except maternal smoking during pregnancy, for which the penalised estimate no longer reached that level (Firth *p* = 0.061); this covariate is therefore the least robust of the six and is not discussed further. The remaining covariates included in each model did not reach statistical significance (Figure 2, Figure 3, Figure 4 and Figure 5); the complete models, with regression coefficients, standard errors and Firth estimates, are reported in Supplementary Table S11. In the sensitivity analysis in which the same four models retained the complete covariate set, and which listwise deletion restricts to survivors, a maternal educational level greater than or equal to high-school diploma [Exp(B) = 2.980; 95% CI 1.023–8.680; *p* = 0.045] and ROP of any stage [Exp(B) = 0.248; 95% CI 0.063–0.976; *p* = 0.046] were associated with the cognitive outcome, IVH grade III–IV with language [Exp(B) = 0.069; 95% CI 0.007–0.710; *p* = 0.025], PMA at discharge with the motor outcome [Exp(B) = 0.697; 95% CI 0.519–0.937; *p* = 0.017] and male sex with the socio-emotional outcome [Exp(B) = 0.442; 95% CI 0.199–0.984; *p* = 0.045] (Supplementary Tables S12–S15).

### 3.5. Secondary Outcomes

Among survivors, a Bayley-III score in the normal range (≥85, not ≥ 70; Section 2.3) was recorded in 82/135 (60.74%) infants for the cognitive scale, 68/135 (50.37%) for language, 101/135 (74.81%) for motor, and 84/135 (62.22%) for socio-emotional. On multivariable analysis restricted to survivors (suboptimal vs. normal development), the following were significantly associated: for cognitive development, chorioamnionitis [Exp(B) = 0.192; 95% CI 0.043–0.866; *p* = 0.032] (Supplementary Figure S5); for language development, IVH grade III–IV [Exp(B) = 0.105; 95% CI 0.011–0.970; *p* = 0.047] (Supplementary Figure S6); for motor development, early- and/or late-onset sepsis [Exp(B) = 0.023; 95% CI 0.001–0.607; *p* = 0.024] (Supplementary Figure S7); and for socio-emotional development, male sex [Exp(B) = 0.409; 95% CI 0.194–0.863; *p* = 0.019] (Supplementary Figure S8). The remaining covariates included in each model did not reach statistical significance (Supplementary Figures S5–S8).

### 3.6. Additional Analyses

In univariate analysis, numerous prenatal, perinatal and postnatal variables were associated (*p* < 0.05) with at least one of the outcomes considered, in both the composite and the survivors-only comparisons. Significant associations involved in particular postnatal morbidities and treatments (invasive mechanical ventilation, early- and/or late-onset sepsis, ROP, anaemia of prematurity, hyperglycaemia, metabolic acidosis, duration of antibiotic prophylaxis/therapy and of inotropic support), nutritional and growth parameters (total milk intake in the first week of life, duration of parenteral nutrition, postnatal weight loss, and change in head circumference Z-score between DoL 14 and 36 weeks’ PMA) and PMA at discharge, as well as GA, maternal educational level, chorioamnionitis and sex; frequencies, medians (interquartile range) and corresponding *p*-values are reported, for the variables with *p* < 0.10, in Table 1, Table 2, Table 3 and Table 4 and, for every variable analysed, in Supplementary Tables S1–S4. Covariates entered into the multivariable models were selected from those significant at univariate analysis on clinical and causal grounds, with the aid of domain-specific directed acyclic graphs, and after verifying the absence of relevant collinearity. Across all models, the variance inflation factor was <5 for every covariate, the Hosmer–Lemeshow test indicated adequate goodness of fit (*p* between 0.085 and 0.964), and the Nagelkerke R-squared ranged from 0.116 to 0.507 (reported in the legends of Figure 2, Figure 3, Figure 4 and Figure 5 and Supplementary Figures S5–S8 and in Supplementary Tables S7 and S11). The complete primary composite models are provided in Supplementary Table S11; the survivors-only models in Supplementary Tables S16–S19; the composite models retaining the complete covariate set, presented as a sensitivity analysis, in Supplementary Tables S12–S15; and an abridged version restricted to the covariates significant at the nominal level in Supplementary Table S22.

All 146 infants contributed to each of the four primary composite models, in which events per variable were 3.8 for the cognitive, 5.7 for the language, 2.8 for the motor, and 5.2 for the socio-emotional domain; the apparent c-statistic ranged from 0.753 to 0.825 and, after bootstrap correction for optimism, from 0.672 to 0.739, with bootstrap calibration slopes between 0.55 and 0.60. The analytic samples of the four survivors-only models comprised 133 to 135 infants, with events per variable between 2.1 and 17.0, apparent c-statistics between 0.677 and 0.820, optimism-corrected c-statistics between 0.653 and 0.704, and calibration slopes between 0.48 and 0.88, the most favourable values belonging to the only model containing three covariates. Events per variable were therefore below the conventional threshold of 10 in eleven of the twelve models fitted, and the calibration slopes indicate that the regression coefficients require shrinkage in all of them. These results are detailed in Supplementary Table S7.

In the sensitivity analysis in which the four composite models retained the complete covariate set, the covariates that are undefined in the infants who died removed, by listwise deletion, all 11 deaths from the cognitive, language and motor models and 9 of the 11 from the socio-emotional model, because no infant who died had been discharged; the outcome distributions of the cognitive and motor models are consequently identical to those of the corresponding survivors-only models. Events per variable fell to between 1.7 and 4.8, optimism-corrected c-statistics to between 0.598 and 0.692, and bootstrap calibration slopes to between 0.37 and 0.43. In these models, a maternal educational level greater than or equal to a high-school diploma and ROP were associated with the cognitive outcome, IVH grade III–IV with language, PMA at discharge with the motor outcome, and male sex with the socio-emotional outcome (Supplementary Tables S7 and S12–S15). The comparison with the primary models is informative in two directions: the associations with the language and socio-emotional domains are common to both specifications, whereas those with maternal educational level, ROP, and PMA at discharge appear only when the analysis is, in effect, restricted to survivors.

Of the 112 covariate terms fitted across the eight models of Supplementary Tables S12–S19, 50 had fewer than six distinct values and a further nine had coincident 10th, 50th and 90th centiles, leaving 53 continuous terms for which departure from linearity could be tested. The likelihood-ratio test for non-linearity reached the nominal threshold in 4 of these 53 terms (smallest *p* = 0.005) and in none of them after Benjamini–Hochberg adjustment; continuous covariates were therefore retained as linear terms. In a sensitivity analysis, the three models containing one of these four terms were refitted with the covariate entered as a restricted cubic spline. Every covariate significant at the nominal level in the linear models remained significant, in the same direction and with a similar magnitude: PMA at hospital discharge in the motor composite model [Exp(B) 0.697, *p* = 0.017, becoming Exp(B) 0.618, *p* = 0.004], IVH grade III–IV in the language survivors-only model [0.105, *p* = 0.047, becoming 0.105, *p* = 0.048] and sepsis in the motor survivors-only model [0.023, *p* = 0.024, becoming 0.025, *p* = 0.032]. In the motor composite model two further covariates reached the nominal threshold once the head circumference Z-score at 36 weeks’ PMA was entered as a spline, the duration of postnatal corticosteroid therapy [Exp(B) 1.327, *p* = 0.028] and the spline term itself (joint test on two degrees of freedom, *p* = 0.046), the Nagelkerke R-squared rising from 0.439 to 0.499 and the events per variable falling from 1.7 to 1.6; in the language survivors-only model neither spline term was significant (joint *p* = 0.19 for both). Because these additional signals arise in the model with the least favourable ratio of events to variables and rest on a departure from linearity that does not survive control of the false discovery rate, they are more plausibly attributable to overfitting than to a genuine non-linear effect, and the linear specification was retained for the models presented here (Supplementary Table S20). Receiver operating characteristic curves and flexible calibration plots are shown in Supplementary Figures S1 and S2 for the four primary composite models and in Supplementary Figures S3 and S4 for the eight models of Supplementary Tables S12–S19. Because the covariates of the four primary composite models are a subset of those of the corresponding models of Supplementary Tables S12–S15, the linearity of every continuous term entering the primary models was covered by the 53 tests described above.

After control of the false discovery rate among the 81 univariate comparisons performed for each outcome, associations remained significant at a false discovery rate below 5% for 19 of 81 variables for the cognitive composite outcome, nine of 81 for language, 22 of 81 for motor and two of 81 for socio-emotional development, and for 15 of 81 variables for motor development among survivors, whereas none remained significant for the cognitive, language and socio-emotional outcomes among survivors (Supplementary Table S8). In the four primary composite models six covariates reached the nominal threshold, and in the models of Supplementary Tables S12–S19 nine did; none of the fifteen retained significance after Benjamini–Hochberg adjustment, either within its own model (smallest adjusted value q = 0.156) or across the 169 covariate tests of the twelve models fitted, for which the adjusted value was q = 0.507 throughout (Supplementary Table S9).

Beyond the variance inflation factor, which never exceeded 3.88, tolerances did not fall below 0.257, the largest absolute pairwise correlation between two covariates of the same model was 0.687 and the largest condition index of the column-centred and column-scaled design matrix was 7.3 (Supplementary Table S6); because centring lowers the condition indices systematically, whereas the conventional threshold of 30 was derived for the scaled but uncentred matrix, these values are to be read as a comparison between models rather than against that threshold. Gestational age and birth weight were correlated in the cohort (r = 0.620) but were never simultaneously adjusted for, as birth weight was not retained in any of the twelve multivariable models.

Of the 81 candidate explanatory variables listed in Supplementary Table S10, 66 were complete, and 15 had at least one missing value; adding the four domain-specific outcome variables of the survivors-only analysis, which are undefined in the 11 infants who died, 19 variables in all had at least one missing value, the largest proportion of missing values for any variable being 7.5%. Missing values were concentrated in the infants who died: 137 of the 149 missing values (91.9%) occurred in the 11 infants who died between day 8 of life and 24 months’ corrected age. For every variable ascertained at a defined postmenstrual age or day of life, each of these missing values corresponded to an infant who had died before that time point, as verified against the postmenstrual age at death (all 11 infants died between 24 and 41 weeks’ postmenstrual age and none of them had been discharged): all six infants with a missing retinopathy of prematurity status had died before 31 weeks’ postmenstrual age, when screening begins, all eight with a missing auditory brainstem response before 34 weeks’, and all nine with missing bronchopulmonary dysplasia status or missing growth Z-scores before 36 weeks’ (Supplementary Table S21).

The remaining 12 missing values, which occurred in survivors, likewise correspond to quantities that are undefined rather than unobserved: six concern the proportion of maternal milk in the total enteral intake of the first week of life in infants whose total enteral intake over that week was zero, so that the ratio has no denominator; four concern the changes in Z-score of body weight and of head circumference between DoL 14 and 36 weeks’ PMA in the two infants born at 35 and 36 weeks’ GA, for whom that interval does not exist; and two concern the Z-scores of body weight and head circumference at birth in an infant born at 23 weeks’ GA, the INTERGROWTH-21st standards being applicable from 24 weeks + 0 days onwards. Missing data in this database are therefore deterministic rather than random, with the exception of the seven values noted above that existed but were not recorded, and this is why multiple imputation was not applied (Supplementary Table S21).

## 4. Discussion

The objective of this study was to describe, within a single contemporary cohort and with an unusually broad characterisation of the exposures acting during neonatal intensive care, which prenatal, perinatal and postnatal factors are independently associated with long-term neurodevelopment and with the composite outcome of neurodevelopment and survival in infants with GA ≤ 32 weeks and/or BW ≤ 1500 g. Three findings emerge. First, despite numerous associations at univariate analysis, only a small set of factors emerged as independent correlates, with a domain-specific pattern that reproduces what the existing literature would predict: severe IVH for language, sepsis and anaemia requiring transfusion for the motor domain, male sex and sepsis for the socio-emotional domain, and—in the analyses restricted to survivors—chorioamnionitis and maternal educational level for the cognitive domain, ROP and the PMA at discharge in addition. Second, none of the nutritional, metabolic and postnatal-growth variables, which this cohort characterises in unusual detail, were independently associated with any domain. Third, no association, in any of the twelve models fitted, survived control of the false discovery rate, and the optimism-corrected discrimination of the models was modest. The first finding places this cohort within the existing body of evidence; the second and the third are, in our view, the more informative, and they are discussed in Section 4.4 and Section 4.7, respectively.

### 4.1. Comparison with the Literature

At the top of the evidence hierarchy, our results are consistent with the pooled estimates of the available meta-analyses on individual risk factors. The two correlates that emerge in the primary analysis alongside male sex are supported by meta-analytic evidence: severe IVH by a meta-analysis of preterm brain injury [50] and neonatal sepsis by two meta-analyses [51,52]. The correlates that appear only in the analyses restricted to survivors are likewise supported, although the evidence behind them is weaker: ROP by a recent meta-analysis [53] and maternal chorioamnionitis by meta-analytic evidence [54] and by the historical study of Wu and Colford [55]. Anaemia requiring transfusion, which emerges in the primary analysis of the motor domain, and ROP are discussed separately at the end of this section, because for both of them the direction and the certainty of the meta-analytic evidence require comment. No meta-analysis, however, has pooled a complete multivariable risk-factor model analogous to ours, and the two most recent systematic reviews [5,6] could not perform a meta-analysis owing to heterogeneity in the definitions of variables and outcomes and in the assessment tools. It should also be emphasised that most of the correlates we identified (sex, education, IVH, ROP, sepsis, anaemia requiring transfusion) are not randomisable: direct experimental evidence is therefore lacking, and the body of evidence on these prognostic factors is, of necessity, observational, whereas randomised trials concern upstream preventive interventions.

Among observational studies with a similar design, our results agree with cohorts that assessed neurodevelopment with the Bayley scale or Griffiths’ Mental Developmental Scales at 24 months’ CA: IVH/severe brain injury as the main correlate is reported by single- and multi-centre Italian cohorts [56,57] and by the Dutch national cohort [3]; the role of low maternal education by the latter [3] and by a Singapore cohort [58]; and that of male sex by the same Singapore cohort [58] and by an Italian cohort [56], consistent also with national cohorts [2,59]. Some of our findings are, by contrast, original: chorioamnionitis assessed in isolation is not considered in the review by Axford et al. [5] nor analysed as an independent correlate in comparable studies; PMA at discharge is not evaluated in either cohorts or reviews; and the socio-emotional domain is rarely analysed in isolation. Finally, the metabolic-nutritional factors significant at our univariate analysis are poorly represented in the literature.

The two correlates that emerge in the primary analysis of the motor domain deserve separate comment. Anaemia requiring transfusion has not been shown, in randomised trials, to be a modifiable determinant of neurodevelopment: a meta-analysis of six trials comparing restrictive with liberal haemoglobin thresholds in 3483 very low birthweight infants found no difference in all-cause mortality or in the composite of death or neurodevelopmental impairment, with a high certainty of evidence [60], and the 2025 Cochrane update of the same question likewise reports little or no difference between restrictive and liberal thresholds in the outcomes assessed at hospital discharge and at neurodevelopmental follow-up [61]. Observational cohorts, by contrast, associate transfusion exposure itself with worse neurodevelopment after adjustment, with a recent cohort reporting an adjusted odds ratio of 2.47 (95% CI 1.13–5.44) for severe neurodevelopmental impairment in infants born before 32 weeks [62]. The discrepancy between the two bodies of evidence is informative rather than contradictory: trials randomise the transfusion threshold, whereas observational studies compare infants who were transfused with infants who were not, and therefore compare sicker with less sick infants. Our covariate—anaemia requiring one or more red blood cell transfusions—belongs to the second class, and the association we report is best read, consistently with Section 4.3, as reflecting the severity of the underlying illness rather than an effect of the transfusion. The systematic review of anaemia, red blood cell transfusion, cerebral oxygenation, brain injury and neurodevelopmental outcome by Kalteren et al. reaches a similar conclusion and does not permit pooling [63].

For retinopathy of prematurity the meta-analysis by Diggikar et al. reports that any ROP is associated with cognitive impairment or intellectual disability (n = 83,506; OR 2.56, 95% CI 1.40–4.69) and that type 1 or severe ROP carries a higher risk than type 2 ROP at 18 to 24 months (n = 5167; OR 3.56, 95% CI 2.60–4.86), with the certainty of the evidence graded as very low throughout [53]. Our own data are consistent with that low certainty: ROP was associated with the cognitive outcome only in the models retaining the complete covariate set, which listwise deletion restricts to survivors, and not in the primary analysis in which the infants who died also contributed. Cohorts that adjust for birth weight and for the grade of intraventricular haemorrhage have similarly reported that the association between the severity of ROP and neurodevelopmental scores does not survive adjustment, which is what our own comparison between the two specifications suggests.

### 4.2. Biological Plausibility

From an interpretative standpoint, the underlying mechanisms are biologically plausible and partly shared. Severe IVH may cause direct and compressive parenchymal damage, with repercussions particularly on language, which requires the integration of cognitive and motor skills. Sepsis and chorioamnionitis presumably act through inflammatory and oxidative-stress mechanisms during a period of extreme vulnerability and increased blood–brain barrier permeability, with possible epigenetic effects on brain development; because no infant had culture-proven early-onset sepsis, the septic exposure captured in this cohort is entirely late-onset, which locates this mechanism in the postnatal weeks of intensive care rather than at birth, and distinguishes it from the antenatal inflammatory exposure represented by chorioamnionitis. ROP may represent a surrogate of substantial respiratory morbidity and of oxygen exposure, with free-radical damage at the retinal and cerebral level, and may also hamper learning through reduced visual acuity. Maternal educational level presumably reflects socio-economic and environmental-stimulation factors; male sex is a well-recognised unfavourable factor attributable to genetic and neuroendocrine differences in brain development; and PMA at discharge may act as a synthetic surrogate of overall morbidity and of the effects of prolonged hospitalisation.

### 4.3. Markers of Illness Severity and the Limits of Causal Interpretation

Several of the variables associated with the outcomes in this study are best understood as markers of the severity of the underlying illness rather than as causes of impaired neurodevelopment. The PMA at hospital discharge is not an exposure but a summary of everything that happened during the stay; the duration of antibiotic prophylaxis and therapy indexes the number and the severity of the suspected or proven infections; the number of red blood cell transfusions indexes the degree of anaemia and of iatrogenic blood loss in the sickest infants; and the duration of postnatal corticosteroid therapy indexes the severity of the respiratory disease that made that treatment necessary. Late-onset sepsis, which is the whole of the septic exposure in this cohort, belongs to the same category: it arises in the infants exposed longest to central lines, parenteral nutrition and invasive ventilation, and therefore indexes the cumulative burden of intensive care as much as the infection itself. This is a deliberate feature of the study design rather than an accident of the analysis: wherever possible we chose to characterise a morbidity not by its mere presence but by the degree of severity that clinical practice uses to grade it, so that anaemia entered the analysis as anaemia requiring one or more red blood cell transfusions, and respiratory disease as disease requiring a given duration of ventilatory or corticosteroid treatment. The corollary is that the associations reported for these variables must not be read as implying that shortening a course of antibiotics, withholding a transfusion or discharging an infant earlier would improve neurodevelopment: the association would in all likelihood persist unchanged, because what is being measured is the severity of the disease and not the treatment. Only trials of different treatment strategies could address that question, and the present data cannot.

### 4.4. Nutritional Variables

The study collected unusually detailed nutritional information, and several nutritional variables—the day of initiation of enteral feeding, the total and the maternal milk intake in the first week of life, the duration of parenteral nutrition—were associated with the outcomes at univariate analysis; few of them entered the multivariable models, and none emerged as an independent correlate. Four explanations, which are not mutually exclusive, deserve consideration. The first is statistical power: with 34 to 68 events per model (Supplementary Table S7) and a large covariate set, the confidence intervals around the nutritional coefficients were wide, and an effect of the magnitude plausible for a single nutritional exposure over a two-year horizon would not have been detected reliably. The second is mediation: enteral feeding is interrupted, and parenteral nutrition prolonged, precisely by the morbidities that also predict neurodevelopment—NEC, sepsis, haemodynamic instability—so that a nutritional variable entered together with those morbidities is adjusted for the very pathway through which it plausibly acts, and its coefficient is attenuated towards the null [64]. The third is collinearity and redundancy: the nutritional variables measure overlapping quantities (maternal milk intake and total milk intake in the first week of life were correlated at r = 0.687, and the proportion of maternal milk is by construction the ratio of the two), and the composition of a given milk is fixed, so that entering them together would inflate the variance of each coefficient without adding information; some of these variables were consequently not carried forward. The fourth is a genuine absence of association at 24 months’ CA, at least for the aspects of nutrition captured here and at a horizon at which the effect of nutrition may be difficult to separate from that of the illness which dictated it. Our data cannot discriminate between these explanations, and we regard the absence of nutritional factors from the final models as an open question rather than as evidence that early nutrition is unimportant for later neurodevelopment.

### 4.5. Clinical Implications

From a clinical perspective, the factors identified here are best used for prognostic stratification and for the organisation of follow-up rather than as therapeutic targets. Severe IVH, sepsis and anaemia requiring transfusion identify, in the primary analysis, infants whose probability of an adverse neurodevelopmental outcome is higher, and non-modifiable characteristics such as male sex add to that stratification; ROP, maternal educational level and the PMA at discharge, which emerged only in the analyses restricted to survivors, may serve the same purpose in infants who reach discharge; together they may guide family counselling, the intensity of neurodevelopmental surveillance and the timing of referral to early-intervention programmes. Whether preventing or attenuating these morbidities would in itself improve neurodevelopment is a different question, which an observational study cannot answer: reducing the incidence of IVH, of nosocomial sepsis and of severe ROP is desirable on its own merits, and it is plausible that neurodevelopment would benefit, but our data provide no evidence of a causal effect and should not be used to justify any specific intervention, which would require trials of the interventions themselves. Future research should test these associations in larger, multicentre cohorts with follow-up extended well beyond 24 months’ CA. The associations found for the cognitive domain call for particular caution, since no covariate was associated with that domain in the primary analysis, in which the infants who died also contributed to the composite outcome (Figure 2 and Supplementary Table S11).

### 4.6. Strengths

Strengths of this study include its prospective design with consecutive enrolment, its single-centre setting with homogeneous clinical management and consistent disease definitions over time, and a recent and narrow enrolment window (2018–2022), which limit the heterogeneity that affects cohorts and reviews with wide enrolment intervals. The study included a particularly broad set of variables, including metabolic, nutritional and growth variables that are rarely considered, and adopted a composite outcome incorporating mortality as a competing risk, complemented by a survivors-only sensitivity analysis. The assessment, domain-specific and inclusive of the socio-emotional domain analysed in isolation, was performed with a single standardised scale (Bayley-III) by a single, independent and experienced examiner.

The breadth of the variable set is itself a strength: rather than isolating one exposure at a time, the study describes the simultaneous action of clinical, nutritional, metabolic and growth-related factors within the same infants, which is the situation the neonatologist actually faces, and the price paid for this breadth in terms of events per variable has been quantified rather than concealed. The comparison of the infants lost to follow-up with those analysed, showing negligible imbalance in every characteristic examined, is a further element in favour of the internal validity of the results.

### 4.7. Limitations

The limitations must be acknowledged with equal candour. The modest sample size, combined with the large number of covariates, results in a low number of events per variable in most models, with wide confidence intervals and a risk of overfitting; the analyses are multiple and, in the primary inference, not adjusted for multiplicity, the false discovery rate having been controlled as a sensitivity analysis, and are therefore exploratory and hypothesis-generating. Internal validation quantified the magnitude of this problem: with events per variable below the conventional threshold of 10 in eleven of the twelve models fitted, and between 2.8 and 5.7 in the four primary composite models, bootstrap calibration slopes of 0.37 to 0.88 across all the models and of 0.55 to 0.60 in the primary ones indicate that the regression coefficients on the log-odds scale are, on average, approximately twofold too extreme, so that the reported odds ratios require shrinkage towards unity by approximately taking their square root, and optimism-corrected c-statistics between 0.598 and 0.739 indicate modest rather than good discriminative ability; the effect estimates presented here should therefore be read as upper bounds of the true associations rather than as transportable effect sizes, the models are not suitable for individual risk prediction, and any future use of them would require external validation together with recalibration. The single-centre origin, while ensuring homogeneity, limits generalisability. The survivors-only analysis is conditioned on survival, with a potential selection bias. The choice of a composite outcome deserves comment in its own right. Combining death with neurodevelopmental impairment avoids the selection bias that arises when only survivors are analysed and keeps the whole cohort under analysis, but it carries two costs: it assigns the same weight to death and to a Bayley-III score below 85, endpoints that are plainly not equivalent for families or for clinicians, and it does not allow the effect of a factor on mortality to be distinguished from its effect on neurodevelopment. Alternative approaches exist. Cause-specific hazard models, or subdistribution hazard models of the Fine and Gray type, would treat death as a competing event and would estimate the two components separately, and two features of the present data argue against them. The first, and the more fundamental, is that neurodevelopment is not a time-to-event outcome: it has no time of onset and was ascertained at a single fixed horizon, 24 months’ corrected age, at which vital status was known for every infant, so that no competing-risk formulation can model both components of the composite. Such a model could describe mortality alone—the individual times to death were in fact recorded, all 11 deaths having occurred in hospital between 24 and 41 weeks’ postmenstrual age—but mortality alone is not the question this study set out to answer. The second is the number of events: with 11 deaths, a subdistribution hazard model adjusted for even a small number of covariates would not be estimable with any useful precision. At a fixed horizon and with complete ascertainment of mortality, the binary composite corresponds to the cumulative incidence of the combined event, and the survivors-only analysis presented alongside it acts as the sensitivity analysis for the weighting that the composite implies. A competing-risk formulation would nevertheless be preferable in a study designed with repeated assessments over time, and we regard the parallel presentation of the two outcomes as a partial rather than a complete substitute for it. At a fixed horizon the unequal clinical importance of the two components could also have been addressed without time-to-event data, either by a multinomial model with three outcome states (death, pathological score, normal score) or by a hierarchical ordinal ranking of the composite endpoint; both were considered and neither was pursued, because with 11 deaths the multinomial model would have had almost no information for the death category and the ordinal approach would have required a weighting of the two endpoints that our data cannot inform. A further limitation concerns the price paid to make the composite outcome operative. Because the postmenstrual age at discharge, the retinopathy status and the growth Z-scores at 36 weeks’ postmenstrual age are undefined in an infant who dies before the corresponding time point, and no infant who died had been discharged, these covariates had to be excluded from the primary composite models in order for the 11 deaths to contribute at all; the primary models are therefore adjusted for a smaller set of confounders than the models of Supplementary Tables S12–S15, which retain the complete set but which listwise deletion restricts to survivors. Neither specification is free of a defect, and we present both: the primary models purchase the inclusion of the deaths at the cost of a less complete adjustment, the sensitivity models purchase the complete adjustment at the cost of the conditioning on survival that the composite outcome was designed to avoid. The associations with the language and the socio-emotional domains are common to the two specifications; those with maternal educational level, retinopathy of prematurity, and the postmenstrual age at discharge appear only in the sensitivity models and are correspondingly more likely to reflect conditioning on survival, residual confounding, or both. This is an additional reason for regarding the domain-specific associations reported here as hypotheses rather than as established effects. Finally, follow-up is limited to 24 months’ CA and does not include variables relating to the period after hospital discharge, although this cohort will continue to be followed over time. Three further considerations temper the interpretation of these results. The first is multiplicity: 648 univariate comparisons and 169 covariate tests within the twelve multivariable models were performed, and while a substantial number of the univariate associations withstood control of the false discovery rate, none of the multivariable associations did, so that the individual correlates identified here are to be regarded as candidate signals awaiting confirmation rather than as established effects. We regard this as a result in its own right and not only as a limitation: it quantifies, in a cohort of the size that single-centre neonatal follow-up studies typically achieve, how little of what reaches nominal significance would survive a correction for multiplicity, and it suggests that comparable cohorts reporting uncorrected associations are reporting signals of similar fragility. The second is residual confounding: the variables available to us describe the pregnancy, the perinatal period and the neonatal hospital stay, whereas no information was collected on parental characteristics other than maternal educational level, on the home environment, or on rehabilitation and nutrition after discharge, all of which plausibly influence neurodevelopment at 24 months’ corrected age and are correlated with the exposures examined; the estimates reported here are therefore adjusted for the measured confounders only. The third concerns causality: the design is observational, and most of the correlates identified cannot be randomised, so that the associations reported in this study, however consistent with biologically plausible mechanisms and with the available meta-analytic evidence, do not by themselves establish causation. For all these reasons, external validation in independent, multicentre cohorts, with recalibration of the models and with follow-up extended beyond 24 months’ corrected age, is a precondition for any clinical application of these findings.

Two potential sources of selection bias deserve separate mention. The first is loss to follow-up, which affected 29 of the 164 eligible survivors (17.68%): the comparison reported in Table 5 shows negligible imbalance in every characteristic examined, but the comparison is limited to the variables available for these infants and has low power, so that a bias operating through unmeasured characteristics—for example family engagement with follow-up, which may itself be associated with the developmental environment—cannot be excluded. The second is the exclusion of the 13 infants who died within the first week of life, which conditions the whole analysis on survival to day 7: the results describe the infants who reach the phase of neonatal intensive care that this study set out to examine, and factors acting mainly through very early death are correspondingly under-represented. The phenotype of the 11 infants who died after day 7 illustrates the same gradient: none had received antenatal corticosteroids, all had required invasive mechanical ventilation and almost all had severe intraventricular haemorrhage or late-onset sepsis, so that the exposures acting before or immediately after birth enter the composite outcome only through infants of this extreme phenotype.

The external validity of these findings deserves explicit comment. The cohort was recruited in a single Italian level III unit, and while this ensures that the indications for ventilation, transfusion, antibiotic therapy, advancement of enteral feeding and discharge were uniform across the whole cohort, and that the definitions of the morbidities were applied consistently by the same team, it also means that the estimates reported here are conditional on the case mix, the care practices and the social context of one centre within one national health system. The frequency of the exposures themselves—antenatal corticosteroid coverage, the proportion of infants receiving maternal milk, the incidence of late-onset sepsis and of severe ROP—differs substantially between countries and between units of the same country, and the maternal educational level that emerged as a correlate of the cognitive outcome is a proxy for a socio-economic and educational environment specific to this setting; both the magnitude and, for the weaker associations, the direction of the effects might therefore differ elsewhere. Neurodevelopment was moreover assessed at a single time point, 24 months’ CA, at which developmental testing has limited ability to predict cognitive functioning at school age, so that the factors identified here as correlates of early development are not necessarily those that will matter for later intellectual, executive and behavioural outcomes [6]. For these reasons, we regard multicentre replication and longer follow-up as necessary next steps rather than as optional refinements, and we intend to extend the study in both directions, enrolling infants from additional centres and following the present cohort beyond 24 months’ CA, in order to establish which of these associations are robust across settings and persist with age.

A further limitation, which defines the direction of our next analyses, concerns the period after discharge. The exposures examined here were deliberately confined to the prenatal, perinatal and postnatal period up to discharge from the neonatal unit, because the question we set out to answer was what happens to a preterm infant during neonatal intensive care and what of it is associated with later neurodevelopment. Rehabilitation and early-intervention programmes, nutritional status and growth after discharge, and the social and family environment in which the child grows are known to influence neurodevelopment at 24 months’ CA and beyond, and their omission is a source of residual confounding for the estimates reported here. Adding them to the present models would have further worsened an already unfavourable ratio of events to variables; we therefore plan to include them in future analyses conducted on a larger cohort, in which a greater number of events will allow a correspondingly greater number of covariates to be examined with an acceptable risk of overfitting.

## 5. Conclusions

In this contemporary cohort of high-risk preterm infants, characterised by unusual breadth across clinical, nutritional, metabolic and growth-related exposures, three results stand out. The domain-specific pattern expected from the existing literature was reproduced: severe IVH for language, sepsis and anaemia requiring transfusion for the motor domain, and male sex and sepsis for the socio-emotional domain, with chorioamnionitis, maternal educational level, ROP and the PMA at discharge appearing in the analyses restricted to survivors. No nutritional, metabolic, or postnatal-growth variable was independently associated with any domain, an informative null in an area in which the published evidence is almost uniformly positive and rarely adjusted for the morbidities that mediate it. And no association, in any of the twelve models fitted, survived control of the false discovery rate, while optimism-corrected discrimination remained modest (c-statistic 0.598 to 0.739). Given the observational and single-centre design of the study and the exploratory character of the analyses, these findings represent associations rather than evidence of causality, several of them plausibly reflecting the severity of the underlying illness; they are hypothesis-generating, and they also delimit what a single-centre cohort of this size can and cannot establish. Multicentre external validation and follow-up extended beyond 24 months’ CA are accordingly necessary next steps rather than optional refinements.

## Figures and Tables

**Table 1:** Characteristics of the study population in relation to (1) composite outcome of cognitive development at 24 months’ CA and death between DoL 8 and neurodevelopmental assessment at 24 months’ CA, and (2) cognitive development at 24 months’ CA for long-term survivors. Only the variables with *p* < 0.10 in at least one of the two comparisons are shown; the complete table, with all the variables analysed, is provided as Supplementary Table S1. Normally distributed continuous variables are expressed as mean ± standard deviation; non-normally distributed continuous variables are expressed as median and interquartile range.

		Cognitive Development and Death			Cognitive Development (Survivors)		
		Cases ^b^	Controls ^c^	*p*-Value	Cases ^d^	Controls ^c^	*p*-Value
	Patients, N (%)	64 (43.84)	82 (56.16)	-	53 (39.26)	82 (60.74)	-
	Score, median (IQR)	80.00 (9.00) *	95.00 (10.00) *	<0.001 *	80.00 (9.00) *	95.00 (10.00) *	<0.001 *
**Prenatal: maternal data**	Maternal age, years	32.00 (9.00) *	35.50 (8.00) *	0.041 *	32.00 (9.00)	35.50 (8.00)	0.054
	Maternal high school diploma or higher educational level, N (%)	43/64 (67.19) *	67/82 (81.71) *	0.043 *	37/53 (69.81)	67/82 (81.71)	0.109
**Perinatal: delivery and first hour of life**	Chorioamnionitis ^g^, N (%)	9/64 (14.06)	4/82 (4.88)	0.053	9/53 (16.98) *	4/82 (4.88) *	0.020 *
	Antenatal corticosteroids ^j^, number of doses	1.00 (2.00) *	2.00 (1.00) *	0.001 *	1.00 (1.00)	2.00 (1.00)	0.085
	Gestational age, weeks	29.00 (5.00) *	30.50 (3.00) *	<0.001 *	29.00 (3.00) *	30.50 (3.00) *	0.030 *
	Birth weight, g	1084.00 (545.50) *	1267.50 (489.50) *	0.005 *	1200.00 (457.50)	1267.50 (489.50)	0.086
	Head circumference, cm	26.50 (3.85) *	27.80 (2.50) *	0.033 *	27.00 (4.10)	27.80 (2.50)	0.174
	5 min Apgar score	7.00 (1.00) *	8.00 (2.00) *	<0.001 *	8.00 (1.00) *	8.00 (2.00) *	0.020 *
**Postnatal: ventilation**	Invasive mechanical ventilation ^l^, N (%)	29/64 (45.31) *	19/82 (23.17) *	0.005 *	18/53 (33.96)	19/82 (23.17)	0.170
**Postnatal: medications**	Duration of postnatal corticosteroid therapy ^p^, days	0.00 (0.00) *	0.00 (0.00) *	0.008 *	0.00 (0.00) *	0.00 (0.00) *	0.040 *
	Duration of antibiotic prophylaxis/therapy, days	7.00 (7.25) *	5.00 (7.00) *	0.002 *	7.00 (5.00) *	5.00 (7.00) *	0.029 *
	Duration of inotropic support ^r^, days	0.00 (1.00) *	0.00 (0.00) *	<0.001 *	0.00 (0.00)	0.00 (0.00)	0.142
**Postnatal: morbidities**	Intraventricular haemorrhage grade III–IV, N (%)	12/64 (18.75)	7/82 (8.54)	0.069	2/53 (3.77)	7/82 (8.54)	0.482
	Early-onset and/or late-onset sepsis, N (%)	19/64 (29.69) *	3/82 (3.66) *	<0.001 *	9/53 (16.98) *	3/82 (3.66) *	0.012 *
	Retinopathy of prematurity (any stage) ^u^, N (%)	18/58 (31.03) *	9/82 (10.98) *	0.003 *	18/53 (33.96) *	9/82 (10.98) *	0.001 *
	Bronchopulmonary dysplasia ^w^, N (%)	7/55 (12.73) *	1/82 (1.22) *	0.007 *	5/53 (9.43) *	1/82 (1.22) *	0.034 *
	Anaemia requiring one or more red blood cell transfusions ^y^, N (%)	30/64 (46.87) *	15/82 (18.29) *	<0.001 *	20/53 (37.74) *	15/82 (18.29) *	0.012 *
**Postnatal: metabolic disorders**	Hyperglycaemia during hospital stay, N (%)	16/64 (25.00) *	4/82 (4.88) *	<0.001 *	9/53 (16.98) *	4/82 (4.88) *	0.020 *
**Postnatal: acid-base disorders**	Metabolic acidosis during hospital stay, N (%)	21/64 (32.81) *	9/82 (10.98) *	0.001 *	11/53 (20.75)	9/82 (10.98)	0.118
	Respiratory alkalosis during hospital stay, N (%)	6/64 (9.37)	2/82 (2.44)	0.138	6/53 (11.32)	2/82 (2.44)	0.057
**Postnatal: electrolyte disturbances**	Hyponatraemia during hospital stay, N (%)	35/64 (54.69)	33/82 (40.24)	0.083	29/53 (54.72)	33/82 (40.24)	0.099
	Hyperchloraemia during hospital stay, N (%)	37/64 (57.81)	34/82 (41.46)	0.050	30/53 (56.60)	34/82 (41.46)	0.085
**Postnatal: nutritional data**	Start of enteral nutrition, days of life	2.00 (4.00) *	1.00 (1.00) *	0.004 *	1.00 (4.00)	1.00 (1.00)	0.079
	Maternal milk intake DoL 0–7, mL/kg/first week of life	8.58 (42.04) *	50.30 (235.30) *	0.003 *	11.63 (43.61) *	50.30 (235.30) *	0.013 *
	Total milk intake DoL 0–7, mL/kg/first week of life	61.63 (226.23) *	230.12 (381.16) *	<0.001 *	85.23 (262.79) *	230.12 (381.16) *	0.005 *
	Duration of parenteral nutrition, days	13.00 (15.00) *	8.00 (11.00) *	0.005 *	13.00 (15.00) *	8.00 (11.00) *	0.024 *
**Postnatal: body growth**	Postnatal weight loss, %	−13.22 ± 5.73 *	−11.22 ± 3.98 *	0.019 *	−12.48 ± 5.05	−11.22 ± 3.98	0.110
	∆Z-score head circumference 36 weeks’ PMA−DoL 14	−0.90 ± 1.03 *	−0.30 ± 1.11 *	0.002 *	−0.86 ± 1.02 *	−0.30 ± 1.11 *	0.005 *
**Postnatal: hospital discharge**	PMA at hospital discharge, weeks	39.00 (5.00) *	38.00 (2.00) *	0.020 *	39.00 (5.00) *	38.00 (2.00) *	0.020 *

Legend: Cases are infants with a pathological Bayley-III score (<85) on the domain considered or, for the composite outcome, infants who died between DoL 8 and the day of the neurodevelopmental assessment at 24 months’ CA; controls are infants with a normal score (≥85) on the domain considered. *, *p* < 0.05. Variables reaching *p* < 0.05 are marked with an asterisk; *p*-values between 0.05 and 0.10 are shown for completeness and are not to be interpreted as trends towards significance. Variable definitions, footnotes, uperscript letters, and the complete list of abbreviations are given in the legend to Supplementary Table S1.

**Table 2:** Characteristics of the study population in relation to (1) composite outcome of language development at 24 months’ CA and death between DoL 8 and neurodevelopmental assessment at 24 months’ CA, and (2) language development at 24 months’ CA for long-term survivors. Only the variables with *p* < 0.10 in at least one of the two comparisons are shown; the complete table, with all the variables analysed, is provided as Supplementary Table S2. Normally distributed continuous variables are expressed as mean ± standard deviation; non-normally distributed continuous variables are expressed as median and interquartile range.

		Language Development and Death			Language Development (Survivors)		
		Cases ^b^	Controls ^c^	*p*-Value	Cases ^d^	Controls ^c^	*p*-Value
	Patients, N (%)	78 (53.42)	68 (46.58)	-	67 (49.63)	68 (50.37)	-
	Score, median (IQR)	77.00 (11.00) *	91.00 (7.25) *	<0.001 *	77.00 (11.00) *	91.00 (7.25) *	<0.001 *
**Prenatal: pregnancy data**	Maternal smoking during pregnancy, N (%)	20/78 (25.64)	9/68 (13.23)	0.061	17/67 (25.37)	9/68 (13.23)	0.074
	Alterations in Doppler flow velocimetry, N (%)	11/78 (14.10)	18/68 (26.47)	0.062	11/67 (16.42)	18/68 (26.47)	0.155
**Perinatal: delivery and first hour of life**	Chorioamnionitis ^g^, N (%)	10/78 (12.82)	3/68 (4.41)	0.075	10/67 (14.92) *	3/68 (4.41) *	0.038 *
	Caesarean section, N (%)	63/78 (80.77)	62/68 (91.18)	0.074	55/67 (82.09)	62/68 (91.18)	0.120
	Male sex, N (%)	47/78 (60.26)	30/68 (44.12)	0.051	41/67 (61.19) *	30/68 (44.12) *	0.047 *
	Gestational age, weeks	29.00 (4.00) *	30.00 (4.00) *	0.003 *	29.34 ± 2.23 *	30.15 ± 2.34 *	0.043 *
	Birth weight, g	1128.00 (575.00)	1253.50 (417.50)	0.050	1225.00 (538.00)	1253.50 (417.50)	0.335
	Head circumference, cm	26.90 (4.12)	27.95 (2.38)	0.054	27.00 (4.00)	27.95 (2.38)	0.196
**Postnatal: ventilation**	Invasive mechanical ventilation ^l^, N (%)	32/78 (41.03) *	16/68 (23.53) *	0.025 *	21/67 (31.34)	16/68 (23.53)	0.309
**Postnatal: medications**	Duration of postnatal corticosteroid therapy ^p^, days	0.00 (0.00)	0.00 (0.00)	0.059	0.00 (0.00)	0.00 (0.00)	0.195
	Duration of antibiotic prophylaxis/therapy, days	7.00 (5.75) *	5.00 (4.00) *	<0.001 *	7.00 (5.00) *	5.00 (4.00) *	0.002 *
	Duration of inotropic support ^r^, days	0.00 (0.00) *	0.00 (0.00) *	0.014 *	0.00 (0.00)	0.00 (0.00)	0.509
**Postnatal: morbidities**	Intraventricular haemorrhage grade III–IV, N (%)	18/78 (23.08) *	1/68 (1.47) *	<0.001 *	8/67 (11.94) *	1/68 (1.47) *	0.017 *
	Early-onset and/or late-onset sepsis, N (%)	19/78 (24.36) *	3/68 (4.41) *	<0.001 *	9/67 (13.43)	3/68 (4.41)	0.066
	Retinopathy of prematurity (any stage) ^u^, N (%)	19/72 (26.39) *	8/68 (11.76) *	0.028 *	19/67 (28.36) *	8/68 (11.76) *	0.016 *
	Bronchopulmonary dysplasia ^w^, N (%)	7/69 (10.14)	1/68 (1.47)	0.062	5/67 (7.46)	1/68 (1.47)	0.115
	Anaemia requiring one or more red blood cell transfusions ^y^, N (%)	33/78 (42.31) *	12/68 (17.65) *	0.001 *	23/67 (34.33) *	12/68 (17.65) *	0.027 *
**Postnatal: metabolic disorders**	Hyperglycaemia during hospital stay, N (%)	17/78 (21.79) *	3/68 (4.41) *	0.002 *	10/67 (14.92) *	3/68 (4.41) *	0.038 *
	Hypoglycaemia during hospital stay, N (%)	15/78 (19.23)	6/68 (8.82)	0.074	11/67 (16.42)	6/68 (8.82)	0.184
**Postnatal: acid-base disorders**	Metabolic acidosis during hospital stay, N (%)	23/78 (29.49) *	7/68 (10.29) *	0.004 *	13/67 (19.40)	7/68 (10.29)	0.136
**Postnatal: electrolyte disturbances**	Hypernatraemia during hospital stay, N (%)	17/78 (21.79)	7/68 (10.29)	0.061	10/67 (14.92)	7/68 (10.29)	0.417
	Hyponatraemia during hospital stay, N (%)	42/78 (53.85)	26/68 (38.23)	0.059	36/67 (53.73)	26/68 (38.23)	0.071
	Hyperchloraemia during hospital stay, N (%)	43/78 (55.13)	28/68 (41.18)	0.092	36/67 (53.73)	28/68 (41.18)	0.144
**Postnatal: nutritional data**	Start of enteral nutrition, days of life	2.00 (4.00) *	1.00 (1.00) *	0.014 *	1.00 (3.00)	1.00 (1.00)	0.111
	Maternal milk intake DoL 0–7, mL/kg/first week of life	12.13 (74.91) *	43.24 (263.23) *	0.023 *	13.15 (81.88)	43.24 (263.23)	0.070
	Total milk intake DoL 0–7, mL/kg/first week of life	83.22 (255.04) *	234.51 (411.17) *	0.002 *	109.28 (284.39) *	234.51 (411.17) *	0.016 *
	Duration of parenteral nutrition, days	13.50 (15.00) *	8.00 (10.00) *	0.001 *	14.00 (15.00) *	8.00 (10.00) *	0.006 *
**Postnatal: body growth**	Postnatal weight loss, %	−12.72 (6.37)	−11.01 (5.22)	0.055	−12.29 ± 4.51	−11.15 ± 4.36	0.138
	∆Z-score head circumference 36 weeks’ PMA−DoL 14	−0.71 ± 1.11	−0.38 ± 1.11	0.087	−0.67 ± 1.10	−0.38 ± 1.11	0.133
**Postnatal: hospital discharge**	PMA at hospital discharge, weeks	38.00 (5.00) *	38.00 (2.00) *	0.033 *	38.00 (5.00) *	38.00 (2.00) *	0.033 *

For legend, see Table 1.

**Table 3:** Characteristics of the study population in relation to (1) composite outcome of motor development at 24 months’ CA and death between DoL 8 and neurodevelopmental assessment at 24 months’ CA, and (2) motor development at 24 months’ CA for long-term survivors. Only the variables with *p* < 0.10 in at least one of the two comparisons are shown; the complete table, with all the variables analysed, is provided as Supplementary Table S3. Normally distributed continuous variables are expressed as mean ± standard deviation; non-normally distributed continuous variables are expressed as median and interquartile range.

		Motor Development and Death			Motor Development (Survivors)		
		Cases ^b^	Controls ^c^	*p*-Value	Cases ^d^	Controls ^c^	*p*-Value
	Patients, N (%)	45 (30.82)	101 (69.18)	-	34 (25.18)	101 (74.81)	-
	Score, median (IQR)	79.00 (12.00) *	97.00 (10.50) *	<0.001 *	79.00 (12.00) *	97.00 (10.50) *	<0.001 *
**Prenatal: pregnancy data**	Maternal alcohol consumption during pregnancy, N (%)	2/45 (4.44)	0/101 (0.00)	0.094	2/34 (5.88)	0/101 (0.00)	0.062
	Maternal diabetes during pregnancy ^e^, N (%)	9/45 (20.00)	10/101 (9.90)	0.094	5/34 (14.71)	10/101 (9.90)	0.528
**Perinatal: delivery and first hour of life**	Chorioamnionitis ^g^, N (%)	6/45 (13.33)	7/101 (6.93)	0.221	6/34 (17.65)	7/101 (6.93)	0.091
	Antenatal corticosteroids ^j^, number of doses	1.00 (2.00) *	2.00 (1.00) *	0.005 *	1.00 (1.00)	2.00 (1.00)	0.407
	Caesarean section, N (%)	35/45 (77.78)	90/101 (89.11)	0.072	27/34 (79.41)	90/101 (89.11)	0.156
	Gestational age, weeks	28.00 (4.00) *	30.00 (3.00) *	<0.001 *	29.00 (4.00) *	30.00 (3.00) *	0.002 *
	Birth weight, g	930.00 (526.50) *	1285.00 (391.00) *	<0.001 *	975.00 (522.50) *	1285.00 (391.00) *	<0.001 *
	Head circumference, cm	25.72 ± 2.85 *	27.64 ± 1.89 *	<0.001 *	25.75 (4.13) *	27.80 (2.50) *	<0.001 *
	5 min Apgar score	8.00 (1.00)	8.00 (1.00)	0.072	8.00 (2.00)	8.00 (1.00)	0.798
	Umbilical cord arterial pH	7.29 (0.10)	7.26 (0.08)	0.603	7.29 (0.08)	7.26 (0.08)	0.091
**Postnatal: ventilation**	Invasive mechanical ventilation ^l^, N (%)	27/45 (60.00) *	21/101 (20.79) *	<0.001 *	16/34 (47.06) *	21/101 (20.79) *	0.003 *
	Surfactant ^m^, number of doses	1.00 (2.00)	0.00 (1.00)	0.078	1.00 (1.00)	0.00 (1.00)	0.392
**Postnatal: medications**	Duration of fentanyl administration ^n^, days	0.00 (6.00) *	0.00 (0.00) *	<0.001 *	0.00 (1.00)	0.00 (0.00)	0.085
	Duration of postnatal corticosteroid therapy ^p^, days	0.00 (0.00) *	0.00 (0.00) *	0.018 *	0.00 (0.00)	0.00 (0.00)	0.148
	Duration of diuretic therapy ^q^, days	0.00 (0.00)	0.00 (0.00)	0.083	0.00 (0.00)	0.00 (0.00)	0.403
	Duration of antibiotic prophylaxis/therapy, days	10.00 (11.00) *	5.00 (4.00) *	<0.001 *	9.00 (11.50) *	5.00 (4.00) *	0.013 *
	Duration of inotropic support ^r^, days	0.00 (3.00) *	0.00 (0.00) *	<0.001 *	0.00 (0.00) *	0.00 (0.00) *	0.004 *
**Postnatal: morbidities**	Intraventricular haemorrhage grade III–IV, N (%)	13/45 (28.89) *	6/101 (5.94) *	<0.001 *	3/34 (8.82)	6/101 (5.94)	0.691
	Electrical-only and/or electroclinical seizures, N (%)	2/45 (4.44)	0/101 (0.00)	0.094	2/34 (5.88)	0/101 (0.00)	0.062
	Early-onset and/or late-onset sepsis, N (%)	20/45 (44.44) *	2/101 (1.98) *	<0.001 *	10/34 (29.41) *	2/101 (1.98) *	<0.001 *
	Retinopathy of prematurity (any stage) ^u^, N (%)	14/39 (35.90) *	13/101 (12.87) *	0.002 *	14/34 (41.18) *	13/101 (12.87) *	<0.001 *
	Bronchopulmonary dysplasia ^w^, N (%)	8/36 (22.22) *	0/101 (0.00) *	<0.001 *	6/34 (17.65) *	0/101 (0.00) *	<0.001 *
	Anaemia requiring one or more red blood cell transfusions ^y^, N (%)	29/45 (64.44) *	16/101 (15.84) *	<0.001 *	19/34 (55.88) *	16/101 (15.84) *	<0.001 *
**Postnatal: metabolic disorders**	Hyperglycaemia during hospital stay, N (%)	14/45 (31.11) *	6/101 (5.94) *	<0.001 *	7/34 (20.59) *	6/101 (5.94) *	0.019 *
	Hypophosphataemic hypercalcaemia during hospital stay, N (%)	10/45 (22.22)	12/101 (11.88)	0.107	8/34 (23.53)	12/101 (11.88)	0.098
**Postnatal: acid-base disorders**	Metabolic acidosis during hospital stay, N (%)	20/45 (44.44) *	10/101 (9.90) *	<0.001 *	10/34 (29.41) *	10/101 (9.90) *	0.006 *
**Postnatal: electrolyte disturbances**	Hyponatraemia during hospital stay, N (%)	26/45 (57.78)	42/101 (41.58)	0.070	20/34 (58.82)	42/101 (41.58)	0.081
	Hypercalcaemia during hospital stay, N (%)	11/45 (24.44)	16/101 (15.84)	0.216	10/34 (29.41)	16/101 (15.84)	0.083
	Hypocalcaemia during hospital stay, N (%)	11/45 (24.44)	12/101 (11.88)	0.054	7/34 (20.59)	12/101 (11.88)	0.254
	Hypophosphataemia during hospital stay, N (%)	13/45 (28.89)	18/101 (17.82)	0.131	11/34 (32.35)	18/101 (17.82)	0.074
**Postnatal: nutritional data**	Start of enteral nutrition, days of life	2.00 (5.75) *	1.00 (1.00) *	0.006 *	1.00 (4.00)	1.00 (1.00)	0.205
	Maternal milk intake DoL 0–7, mL/kg/first week of life	10.60 (44.64) *	24.33 (187.46) *	0.037 *	12.13 (60.59)	24.33 (187.46)	0.173
	Total milk intake DoL 0–7, mL/kg/first week of life	31.93 (187.82) *	223.33 (370.43) *	<0.001 *	38.67 (235.57) *	223.33 (370.43) *	<0.001 *
	Duration of parenteral nutrition, days	17.00 (18.00) *	9.00 (10.00) *	<0.001 *	17.00 (19.00) *	9.00 (10.00) *	<0.001 *
**Postnatal: body growth**	Postnatal weight loss, %	−14.08 ± 5.78 *	−11.21 ± 4.20 *	0.004 *	−13.21 ± 4.90 *	−11.21 ± 4.20 *	0.023 *
	Body weight at 36 weeks’ PMA Z-score	−1.95 (2.01)	−1.43 (1.77)	0.056	−1.95 (2.00) *	−1.43 (1.77) *	0.047 *
	Head circumference at 36 weeks’ PMA Z-score	−1.65 (1.66) *	−0.46 (1.75) *	<0.001 *	−1.57 (1.67) *	−0.46 (1.75) *	0.001 *
	∆Z-score head circumference 36 weeks’ PMA−DoL 14	−1.07 ± 1.10 *	−0.36 ± 1.06 *	<0.001 *	−1.02 ± 1.10 *	−0.36 ± 1.06 *	0.002 *
**Postnatal: hospital discharge**	PMA at hospital discharge, weeks	40.00 (5.00) *	38.00 (2.00) *	<0.001 *	40.00 (5.00) *	38.00 (2.00) *	<0.001 *

For legend, see Table 1.

**Table 4:** Characteristics of the study population in relation to (1) composite outcome of socio-emotional development at 24 months’ CA and death between DoL 8 and neurodevelopmental assessment at 24 months’ CA, and (2) socio-emotional development at 24 months’ CA for long-term survivors. Only the variables with *p* < 0.10 in at least one of the two comparisons are shown; the complete table, with all the variables analysed, is provided as Supplementary Table S4. Normally distributed continuous variables are expressed as mean ± standard deviation; non-normally distributed continuous variables are expressed as median and interquartile range.

		Socio-Emotional Development and Death			Socio-Emotional Development (Survivors)		
		Cases^b^	Controls ^c^	*p*-Value	Cases ^d^	Controls ^c^	*p*-Value
	Patients, N (%)	62 (42.47)	84 (57.53)	-	51 (37.78)	84 (62.22)	-
	Score, median (IQR)	80.00 (9.00) *	105.00 (15.00) *	<0.001 *	80.00 (9.00) *	105.00 (15.00) *	<0.001 *
**Prenatal: maternal data**	Maternal university degree or higher educational level, N (%)	13/62 (20.97)	29/84 (34.52)	0.074	13/51 (25.49)	29/84 (34.52)	0.272
**Prenatal: pregnancy data**	Maternal smoking during pregnancy, N (%)	17/62 (27.42) *	12/84 (14.29) *	0.049 *	14/51 (27.45)	12/84 (14.29)	0.060
	Maternal pressure disorders during pregnancy, N (%)	12/62 (19.35)	27/84 (32.14)	0.084	11/51 (21.57)	27/84 (32.14)	0.185
**Perinatal: delivery and first hour of life**	Male sex, N (%)	39/62 (62.90) *	38/84 (45.24) *	0.035 *	33/51 (64.71) *	38/84 (45.24) *	0.028 *
	Gestational age, weeks	29.00 (4.00) *	30.00 (3.00) *	0.004 *	29.00 (3.00)	30.00 (3.00)	0.109
	Birth weight, g	1192.50 (610.00)	1247.50 (450.25)	0.075	1245.00 (505.00)	1247.50 (450.25)	0.610
	Head circumference, cm	26.53 ± 2.74 *	27.42 ± 2.03 *	0.032 *	27.50 (4.10)	27.75 (3.30)	0.301
	Twin infants, N (%)	19/62 (30.64)	18/84 (21.43)	0.206	18/51 (35.29)	18/84 (21.43)	0.077
	5 min Apgar score	8.00 (1.00)	8.00 (1.00)	0.619	8.00 (1.00)	8.00 (1.00)	0.071
**Postnatal: ventilation**	Invasive mechanical ventilation ^l^, N (%)	27/62 (43.55) *	21/84 (25.00) *	0.018 *	16/51 (31.37)	21/84 (25.00)	0.421
**Postnatal: medications**	Duration of fentanyl administration ^n^, days	0.00 (2.25)	0.00 (0.00)	0.079	0.00 (0.00)	0.00 (0.00)	0.749
	Duration of antibiotic prophylaxis/therapy, days	7.00 (6.25) *	5.00 (7.00) *	0.011 *	5.00 (7.00)	5.00 (7.00)	0.128
	Duration of inotropic support ^r^, days	0.00 (1.00) *	0.00 (0.00) *	0.002 *	0.00 (0.00)	0.00 (0.00)	0.358
**Postnatal: morbidities**	Necrotising enterocolitis (any stage), N (%)	7/62 (11.29)	3/84 (3.57)	0.097	3/51 (5.88)	3/84 (3.57)	0.673
	Early-onset and/or late-onset sepsis, N (%)	18/62 (29.03) *	4/84 (4.76) *	<0.001 *	8/51 (15.69)	4/84 (4.76)	0.057
	Retinopathy of prematurity (any stage) ^u^, N (%)	15/56 (26.79)	12/84 (14.29)	0.066	15/51 (29.41) *	12/84 (14.29) *	0.033 *
	Jaundice requiring phototherapy and/or exchange transfusion ^x^, N (%)	45/62 (72.58)	55/84 (65.48)	0.361	41/51 (80.39)	55/84 (65.48)	0.064
	Anaemia requiring one or more red blood cell transfusions ^y^, N (%)	26/62 (41.93) *	19/84 (22.62) *	0.012 *	16/51 (31.37)	19/84 (22.62)	0.260
**Postnatal: metabolic disorders**	Hyperglycaemia during hospital stay, N (%)	14/62 (22.58) *	6/84 (7.14) *	0.007 *	7/51 (13.72)	6/84 (7.14)	0.238
**Postnatal: acid-base disorders**	Metabolic acidosis during hospital stay, N (%)	21/62 (33.87) *	9/84 (10.71) *	<0.001 *	11/51 (21.57)	9/84 (10.71)	0.085
**Postnatal: nutritional data**	Start of enteral nutrition, days of life	2.00 (4.00)	1.00 (1.75)	0.073	1.00 (2.00)	1.00 (1.75)	0.531
	Maternal milk intake DoL 0–7, mL/kg/first week of life	11.44 (80.51)	23.98 (220.22)	0.090	12.26 (106.07)	23.98 (220.22)	0.273
	Total milk intake DoL 0–7, mL/kg/first week of life	65.65 (253.12) *	223.91 (387.60) *	0.006 *	85.23 (304.02)	223.91 (387.60)	0.060
	Duration of parenteral nutrition, days	13.50 (15.00) *	9.00 (12.00) *	0.009 *	14.00 (15.00) *	9.00 (12.00) *	0.041 *
**Postnatal: body growth**	Postnatal weight loss, %	−13.06 ± 5.86	−11.39 ± 3.96	0.055	−12.26 ± 5.17	−11.39 ± 3.96	0.305
	∆Z-score head circumference 36 weeks’ PMA−DoL 14	−0.80 ± 1.03 *	−0.38 ± 1.15 *	0.036 *	−0.75 ± 1.01	−0.38 ± 1.15	0.066

For legend, see Table 1.

**Table 5:** Comparison of the prenatal, perinatal and postnatal characteristics of the infants lost to follow-up with those of the infants assessed at 24 months’ CA and of the whole analysed cohort. Continuous variables are expressed as median and interquartile range.

Variable	Lost to Follow-Up (n = 29)	Assessed at 24 Months’ CA (n = 135)			Analysed Cohort (n = 146)		
		Value	Std. diff.	*p*-Value	Value	Std. diff.	*p*-Value
**Gestational age, weeks**	29.00 (28.00–31.00)	30.00 (28.00–31.00)	−0.08	0.672	30.00 (28.00–31.00)	0.04	0.937
**Birth weight, g**	1310.00 (820.00–1400.00)	1245.00 (995.00–1443.00)	−0.04	0.969	1232.50 (960.00–1427.50)	0.06	0.716
**Head circumference at birth, cm**	27.50 (26.00–28.70)	27.50 (25.55–28.95)	−0.09	0.766	27.50 (25.42–28.90)	0.01	0.986
**5 min Apgar score**	8.00 (7.00–8.00)	8.00 (7.00–8.00)	−0.06	0.921	8.00 (7.00–8.00)	0.02	0.767
**Male sex, N (%)**	15/29 (51.72)	71/135 (52.59)	−0.02	1.000	77/146 (52.74)	−0.02	1.000
**Twin infants, N (%)**	8/29 (27.59)	36/135 (26.67)	0.02	1.000	37/146 (25.34)	0.05	0.818
**Maternal ethnicity: Caucasian, N (%)**	25/29 (86.21)	119/135 (88.15)	−0.06	0.758	128/146 (87.67)	−0.04	0.765
**Maternal high school diploma or higher educational level, N (%)**	22/29 (75.86)	104/135 (77.04)	−0.03	1.000	110/146 (75.34)	0.01	1.000
**Antenatal corticosteroids, any dose, N (%)**	24/29 (82.76)	115/135 (85.19)	−0.07	0.777	115/146 (78.77)	0.10	0.803
**Antenatal corticosteroids, complete course, N (%)**	15/29 (51.72)	72/135 (53.33)	−0.03	1.000	72/146 (49.32)	0.05	0.841
**Chorioamnionitis, N (%)**	3/29 (10.34)	13/135 (9.63)	0.02	1.000	13/146 (8.90)	0.05	0.732
**Caesarean section, N (%)**	25/29 (86.21)	117/135 (86.67)	−0.01	1.000	125/146 (85.62)	0.02	1.000
**Invasive mechanical ventilation, N (%)**	9/29 (31.03)	37/135 (27.41)	0.08	0.820	48/146 (32.88)	−0.04	1.000
**Intraventricular haemorrhage grade III–IV, N (%)**	3/29 (10.34)	9/135 (6.67)	0.13	0.446	19/146 (13.01)	−0.08	1.000
**Culture-proven early-onset sepsis, N (%)**	0/29 (0.00)	0/135 (0.00)	0.00	1.000	0/146 (0.00)	0.00	1.000
**Culture-proven late-onset sepsis, N (%)**	4/29 (13.79)	12/135 (8.89)	0.16	0.488	22/146 (15.07)	−0.04	1.000
**Retinopathy of prematurity (any stage), N (%)**	6/29 (20.69)	27/135 (20.00)	0.02	1.000	27/140 (19.29)	0.04	0.802
**Bronchopulmonary dysplasia, N (%)**	2/29 (6.90)	6/135 (4.44)	0.11	0.632	8/137 (5.84)	0.04	0.687
**Necrotising enterocolitis (any stage), N (%)**	2/29 (6.90)	6/135 (4.44)	0.11	0.632	10/146 (6.85)	0.00	1.000
**Treated patent ductus arteriosus, N (%)**	7/29 (24.14)	28/135 (20.74)	0.08	0.803	36/146 (24.66)	−0.01	1.000
**PMA at hospital discharge, weeks**	38.00 (37.00–40.00)	38.00 (37.00–40.00)	−0.01	0.962	38.00 (37.00–40.00)	−0.01	0.962

Legend: Lost to follow-up, infants eligible for the neurodevelopmental assessment at 24 months’ CA who were not assessed (n = 29); Assessed at 24 months’ CA, survivors with a complete Bayley-III assessment (n = 135); Analysed cohort, all infants included in the analysis, that is the 135 infants assessed plus the 11 infants who died between DoL 8 and 24 months’ CA (n = 146). Std. diff., standardised difference, calculated as the difference between the two means, or between the two proportions, divided by the pooled standard deviation; absolute values below 0.10 indicate negligible imbalance and values below 0.20 indicate acceptable imbalance, irrespective of sample size. *p*-values are from the Mann–Whitney U test for continuous variables and from Fisher’s exact test for categorical variables and are reported for completeness only: with 29 infants in the smaller group, these tests have low power, and a non-significant *p*-value must not be interpreted as evidence of the absence of a difference. Denominators are given for every categorical variable; for retinopathy of prematurity, bronchopulmonary dysplasia and PMA at hospital discharge, the denominator of the analysed cohort is smaller than 146 because these variables are undefined in the infants who died before the corresponding time point (Supplementary Table S21). No infant in either group had culture-proven early-onset sepsis. List of abbreviations: CA, corrected age; DoL, day of life; PMA, postmenstrual age; Std. diff., standardised difference.

## Data Availability

The data presented in this study are available on request from the corresponding author. The data are not publicly available owing to privacy and ethical restrictions.

## Supplementary Materials

The following supporting information can be downloaded at https://www.mdpi.com/article/10.3390/children13091214/s1, Supplementary Tables S1–S4: Complete versions of Table 1, Table 2, Table 3 and Table 4, containing every variable analysed; Supplementary Table S5: Edge lists of the domain-specific directed acyclic graphs used for covariate selection; Supplementary Table S6: Collinearity diagnostics beyond the variance inflation factor; Supplementary Table S7: Events per variable, apparent and optimism-corrected c-statistic, and bootstrap calibration slope for each of the twelve multivariable models; Supplementary Table S8: Control of the false discovery rate among the univariate comparisons; Supplementary Table S9: Control of the false discovery rate among the covariates of the multivariable models; Supplementary Table S10: Analysis variables grouped by category (prenatal, perinatal, postnatal clinical, nutritional, growth, metabolic, acid-base and electrolyte); Supplementary Table S11: Complete results of the four primary composite models shown in Figure 2, Figure 3, Figure 4 and Figure 5, restricted to the covariates defined in every infant so that all 146 infants contribute, with Firth penalised estimates and internal validation; Supplementary Tables S12–S15: Sensitivity analysis, the four composite-outcome models retaining the complete covariate set, which listwise deletion restricts to survivors; Supplementary Figures S1 and S2: Flexible calibration plots and receiver operating characteristic curves of the four primary composite models shown in Figure 2, Figure 3, Figure 4 and Figure 5; Supplementary Tables S16–S19: The four survivors-only (secondary) models; Supplementary Tables S12–S19 report the regression coefficient B, standard error, Wald statistic, Exp(B), 95% confidence interval and *p*-value for every covariate; Supplementary Figures S3 and S4: Flexible calibration plots and receiver operating characteristic curves of the eight models of Supplementary Tables S12–S19; Supplementary Table S20: Sensitivity analysis in which the three models containing a covariate with a nominal departure from linearity were refitted with that covariate entered as a restricted cubic spline; Supplementary Table S21: Missing data for the analysis variables; Supplementary Figures S5–S8: Multivariable forest plots for the survivors-only (secondary) analyses (cognitive, language, motor and socio-emotional domains); Supplementary Table S22: Abridged version of Supplementary Tables S12–S19, restricted to the covariates significant at the nominal level.

## Abbreviations

BW	birth weight
CA	corrected age
GA	gestational age
IVH	intraventricular haemorrhage
NICU	neonatal intensive care unit
PMA	postmenstrual age
ROP	retinopathy of prematurity
VLBW	very low birth weight
