# Supplementary material for: Risk Factors Influencing Neurodevelopmental Outcome and Survival in Preterm Infants: A Prospective Cohort Study
Source: Children (Basel). 2026 Sep 8;13(9):1214. doi: 10.3390/children13091214 (PMC13605276; doi:10.3390/children13091214)
Supplement: Supplementary file 1 [file children-13-01214-s001.zip › children-4477219-supplementary.pdf]

Supplementary Table S1. Complete version of Table 1: characteristics of the study population in relation to 1) the composite outcome of cognitive development at 24 months' CA and death between DoL 8 and the neurodevelopmental assessment at 24 months' CA, and 2) cognitive development at 24 months' CA for long-term survivors. All the variables analysed are reported, including those not reaching statistical significance. Normally-distributed continuous variables are expressed as mean ± standard deviation; non-normally distributed continuous variables are expressed as median and interquartile range.

		**Cognitive development[a] and Death**			**Cognitive development[a]**		
		**Cases[b]**	**Controls[c]**	**p-value**	**Cases[d]**	**Controls[c]**	**p-value**
	Patients, *N (%)*	64 (43.84)	82 (56.16)	-	53 (39.26)	82 (60.74)	-
	Score, *median (IQR)*	80.00 (9.00)*	95.00 (10.00)*	< 0.001*	80.00 (9.00)*	95.00 (10.00)*	< 0.001*
Prenatal variables: maternal data	Maternal age, *years*	32.00 (9.00)*	35.50 (8.00)*	0.041*	32.00 (9.00)	35.50 (8.00)	0.054
	Maternal ethnicity: Caucasian, *N (%)*	53/64 (82.81)	75/82 (91.46)	0.115	44/53 (83.02)	75/82 (91.46)	0.138
	Maternal high school diploma or higher educational level, *N (%)*	43/64 (67.19)*	67/82 (81.71)*	0.043*	37/53 (69.81)	67/82 (81.71)	0.109
	Maternal university degree or higher educational level, *N (%)*	14/64 (21.87)	28/82 (34.15)	0.104	14/53 (26.41)	28/82 (34.15)	0.343
Prenatal variables: pregnancy data	Maternal smoking during pregnancy, *N (%)*	15/64 (23.44)	14/82 (17.07)	0.339	12/53 (22.64)	14/82 (17.07)	0.423
	Maternal alcohol consumption during pregnancy, *N (%)*	2/64 (3.12)	0/82 (0.00)	0.190	2/53 (3.77)	0/82 (0.00)	0.152
	Maternal drug use during pregnancy, *N (%)*	1/64 (1.56)	0/82 (0.00)	0.438	1/53 (1.89)	0/82 (0.00)	0.393
	Maternal thyroid disorders during pregnancy, *N (%)*	6/64 (9.37)	12/82 (14.63)	0.338	5/53 (9.43)	12/82 (14.63)	0.374
	Maternal diabetes during pregnancy[e], *N (%)*	8/64 (12.50)	11/82 (13.41)	0.871	4/53 (7.55)	11/82 (13.41)	0.289
	Intrauterine growth restriction[f], *N (%)*	10/64 (15.62)	11/82 (13.41)	0.706	9/53 (16.98)	11/82 (13.41)	0.569
	Maternal pressure disorders during pregnancy, *N (%)*	17/64 (26.56)	22/82 (26.83)	0.971	16/53 (30.19)	22/82 (26.83)	0.672
	Alterations of Doppler flow velocimetry, *N (%)*	9/64 (14.06)	20/82 (24.39)	0.121	9/53 (16.98)	20/82 (24.39)	0.306
Perinatal variables: data about delivery and patient in the first hour of life	Placental abruption, *N (%)*	6/64 (9.37)	5/82 (6.10)	0.535	3/53 (5.66)	5/82 (6.10)	1.000
	Chorioamnionitis[g], *N (%)*	9/64 (14.06)	4/82 (4.88)	0.053	9/53 (16.98)*	4/82 (4.88)*	0.020*
	One or more risk factors for intrapartum infection[h], *N (%)*	42/64 (65.62)	46/82 (56.10)	0.243	32/53 (60.38)	46/82 (56.10)	0.623

	Intrapartum antibiotics ≥ 4 hours before birth[i], *N (%)*	16/64 (25.00)	19/82 (23.17)	0.797	13/53 (24.53)	19/82 (23.17)	0.856
	Antenatal corticosteroids[j], *number of doses*	1.00 (2.00)*	2.00 (1.00)*	0.001*	1.00 (1.00)	2.00 (1.00)	0.085
	Caesarean section, *N (%)*	54/64 (84.37)	71/82 (86.58)	0.706	46/53 (86.79)	71/82 (86.58)	0.972
	Male sex, *N (%)*	38/64 (59.37)	39/82 (47.56)	0.156	32/53 (60.38)	39/82 (47.56)	0.145
	Gestational age, *weeks*	29.00 (5.00)*	30.50 (3.00)*	< 0.001*	29.00 (3.00)*	30.50 (3.00)*	0.030*
	Birth weight, *g*	1084.00 (545.50)*	1267.50 (489.50)*	0.005*	1200.00 (457.50)	1267.50 (489.50)	0.086
	Birth weight Z-score[k]	0.03 (1.66)	-0.22 (1.87)	0.577	0.03 (1.72)	-0.22 (1.87)	0.653
	Head circumference, *cm*	26.50 (3.85)*	27.80 (2.50)*	0.033*	27.00 (4.10)	27.80 (2.50)	0.174
	Head circumference Z-score[k]	0.09 (1.48)	-0.03 (1.42)	0.283	0.01 ± 1.14	-0.16 ± 1.02	0.386
	Twin infants, *N (%)*	13/64 (20.31)	24/82 (29.27)	0.217	12/53 (22.64)	24/82 (29.27)	0.395
	5-minute Apgar score	7.00 (1.00)*	8.00 (2.00)*	<0.001*	8.00 (1.00)*	8.00 (2.00)*	0.020*
	Umbilical cord arterial pH	7.25 (0.10)	7.27 (0.08)	0.169	7.26 (0.09)	7.27 (0.08)	0.476
	Umbilical cord arterial BD_{ecf}	-5.80 (5.03)	-5.40 (4.53)	0.248	-4.90 (4.75)	-5.40 (4.53)	0.649
	Body temperature at 1 hour of life, *°C*	36.20 (0.50)	36.10 (0.40)	0.302	36.20 (0.50)	36.10 (0.40)	0.485
Postnatal variables: ventilation data	Invasive mechanical ventilation[l], *N (%)*	29/64 (45.31)*	19/82 (23.17)*	0.005*	18/53 (33.96)	19/82 (23.17)	0.170
	Surfactant[m], *number of doses*	1.00 (1.00)	1.00 (1.00)	0.833	0.00 (1.00)	1.00 (1.00)	0.569
Postnatal variables: medications	Duration of fentanyl administration[n], *days*	0.00 (2.00)	0.00 (0.00)	0.120	0.00 (0.00)	0.00 (0.00)	0.622
	Duration of caffeine administration[o], *days*	22.00 (14.00)	22.00 (15.00)	0.914	22.00 (11.00)	22.00 (15.00)	0.978
	Duration of postnatal corticosteroid therapy[p], *days*	0.00 (0.00)*	0.00 (0.00)*	0.008*	0.00 (0.00)*	0.00 (0.00)*	0.040*
	Duration of diuretic therapy[q], *days*	0.00 (0.00)	0.00 (0.00)	0.703	0.00 (0.00)	0.00 (0.00)	0.653
	Duration of antibiotic prophylaxis/therapy, *days*	7.00 (7.25)*	5.00 (7.00)*	0.002*	7.00 (5.00)*	5.00 (7.00)*	0.029*
	Duration of inotropic support[r], *days*	0.00 (1.00)*	0.00 (0.00)*	< 0.001*	0.00 (0.00)	0.00 (0.00)	0.142

Postnatal variables: morbidities	Haemodynamically significant patent ductus arteriosus[s], *N (%)*	20/64 (31.25)	16/82 (19.51)	0.103	12/53 (22.64)	16/82 (19.51)	0.661
	Necrotising enterocolitis (any stage), *N (%)*	5/64 (7.81)	5/82 (6.10)	0.749	1/53 (1.89)	5/82 (6.10)	0.403
	Intraventricular haemorrhage grade III-IV[t], *N (%)*	12/64 (18.75)	7/82 (8.54)	0.069	2/53 (3.77)	7/82 (8.54)	0.482
	Electrical-only and/or electroclinical seizures, *N (%)*	2/64 (3.12)	0/82 (0.00)	0.190	2/53 (3.77)	0/82 (0.00)	0.152
	Early-onset and/or late-onset sepsis, *N (%)*	19/64 (29.69)*	3/82 (3.66)*	<0.001*	9/53 (16.98)*	3/82 (3.66)*	0.012*
	Retinopathy of prematurity (any stage)[u], *N (%)*	18/58 (31.03)*	9/82 (10.98)*	0.003*	18/53 (33.96)*	9/82 (10.98)*	0.001*
	Congenital hearing loss[v], *N (%)*	1/56 (1.79)	0/82 (0.00)	0.406	1/53 (1.89)	0/82 (0.00)	0.393
	Bronchopulmonary dysplasia[w], *N (%)*	7/55 (12.73)*	1/82 (1.22)*	0.007*	5/53 (9.43)*	1/82 (1.22)*	0.034*
	Jaundice requiring phototherapy and/or exchange transfusion[x], *N (%)*	44/64 (68.75)	56/82 (68.29)	0.953	40/53 (75.47)	56/82 (68.29)	0.369
	Anaemia requiring one or more red blood cell transfusions[y], *N (%)*	30/64 (46.87)*	15/82 (18.29)*	<0.001*	20/53 (37.74)*	15/82 (18.29)*	0.012*
Postnatal variables: metabolic disorders	Hyperglycaemia during HS[z], *N (%)*	16/64 (25.00)*	4/82 (4.88)*	<0.001*	9/53 (16.98)*	4/82 (4.88)*	0.020*
	Hypoglycaemia during HS[&], *N (%)*	12/64 (18.75)	9/82 (10.98)	0.184	8/53 (15.09)	9/82 (10.98)	0.481
	Hypophosphataemic hypercalcaemia during HS[/], *N (%)*	10/64 (15.62)	12/82 (14.63)	0.868	8/53 (15.09)	12/82 (14.63)	0.941
	Hypertriglyceridaemia during HS[(], *N (%)*	7/64 (10.94)	6/82 (7.32)	0.446	5/53 (9.43)	6/82 (7.32)	0.751
Postnatal variables: acid-base disorders	Metabolic acidosis during HS[)], *N (%)*	21/64 (32.81)*	9/82 (10.98)*	0.001*	11/53 (20.75)	9/82 (10.98)	0.118
	Metabolic alkalosis during HS[=], *N (%)*	1/64 (1.56)	0/82 (0.00)	0.438	1/53 (1.89)	0/82 (0.00)	0.393
	Respiratory acidosis during HS[?], *N (%)*	8/64 (12.50)	9/82 (10.98)	0.776	5/53 (9.43)	9/82 (10.98)	0.774
	Respiratory alkalosis during HS^, *N (%)*	6/64 (9.37)	2/82 (2.44)	0.138	6/53 (11.32)	2/82 (2.44)	0.057
Postnatal variables: electrolyte disturbances	Hypernatraemia during HS[∞], *N (%)*	13/64 (20.31)	11/82 (13.41)	0.264	6/53 (11.32)	11/82 (13.41)	0.720
	Hyponatraemia during HS[§], *N (%)*	35/64 (54.69)	33/82 (40.24)	0.083	29/53 (54.72)	33/82 (40.24)	0.099
	Hyperkalaemia during HS [>], *N (%)*	13/64 (20.31)	20/82 (24.39)	0.559	7/53 (13.21)	20/82 (24.39)	0.113
	Hypokalaemia during HS[<], *N (%)*	5/64 (7.81)	4/82 (4.88)	0.506	3/53 (5.66)	4/82 (4.88)	1.000

	Hyperchloraemia during HS[•], *N (%)*	37/64 (57.81)	34/82 (41.46)	0.050	30/53 (56.60)	34/82 (41.46)	0.085
	Hypochloraemia during HS[□], *N (%)*	7/64 (10.94)	10/82 (12.19)	0.814	7/53 (13.21)	10/82 (12.19)	0.863
	Hypercalcaemia during HS[◊], *N (%)*	13/64 (20.31)	14/82 (17.07)	0.617	12/53 (22.64)	14/82 (17.07)	0.423
	Hypocalcaemia during HS[϶], *N (%)*	12/64 (18.75)	11/82 (13.41)	0.380	8/53 (15.09)	11/82 (13.41)	0.784
	Hyperphosphataemia during HS[⌈], *N (%)*	14/64 (21.87)	19/82 (23.17)	0.853	9/53 (16.98)	19/82 (23.17)	0.386
	Hypophosphataemia during HS[⌉], *N (%)*	12/64 (18.75)	19/82 (23.17)	0.517	10/53 (18.87)	19/82 (23.17)	0.552
	Hypermagnesaemia during HS[#], *N (%)*	25/64 (39.06)	31/82 (37.80)	0.877	16/53 (30.19)	31/82 (37.80)	0.364
	Hypomagnesaemia during HS[@], *N (%)*	4/64 (6.25)	9/82 (10.98)	0.320	4/53 (7.55)	9/82 (10.98)	0.510
Postnatal variables: nutritional data	Start of enteral nutrition, *days of life*	2.00 (4.00)*	1.00 (1.00)*	0.004*	1.00 (4.00)	1.00 (1.00)	0.079
	Maternal milk intake DoL 0-7, *ml/kg/first week of life*	8.58 (42.04)*	50.30 (235.30)*	0.003*	11.63 (43.61)*	50.30 (235.30)*	0.013*
	Total milk intake DoL 0-7, *ml/kg/first week of life*	61.63 (226.23)*	230.12 (381.16)*	< 0.001*	85.23 (262.79)*	230.12 (381.16)*	0.005*
	Maternal milk intake DoL 0-7/Total milk intake DoL 0-7, *%*	34.10 (71.31)	33.28 (66.79)	0.619	32.55 (68.26)	33.28 (66.79)	0.533
	Duration of parenteral nutrition, *days*	13.00 (15.00)*	8.00 (11.00)*	0.005*	13.00 (15.00)*	8.00 (11.00)*	0.024*
Postnatal variables: body growth	Postnatal weight loss, *%*	-13.22 ± 5.73*	-11.22 ± 3.98*	0.019*	-12.48 ± 5.05	-11.22 ± 3.98	0.110
	Recovery of birth weight, *DoL*	12.00 (6.25)	12.00 (4.00)	0.597	12.00 (6.50)	12.00 (4.00)	0.643
	Body weight at DoL 14 Z-Score[k]	-1.38 ± 1.11	-1.37 ± 1.04	0.968	-1.31 ± 1.03	-1.37 ± 1.04	0.741
	Head circumference at DoL 14 Z-Score[k]	-0.08 (1.71)	-0.34 (1.42)	0.147	-0.16 ± 1.11	-0.47 ± 1.06	0.108
	Body weight at 36 weeks' PMA Z-Score[k]	-1.55 ± 1.16	-1.54 ± 1.15	0.964	-1.56 ± 1.15	-1.54 ± 1.15	0.945
	Head circumference at 36 weeks' PMA Z-Score[k]	-1.06 ± 1.26	-0.78 ± 1.25	0.209	-1.02 ± 1.27	-0.78 ± 1.25	0.285
	ΔZ-score body weight 36 weeks' PMA-DoL 14[k,σ]	-0.16 (0.67)	-0.18 (0.63)	0.828	-0.16 (0.65)	-0.18 (0.63)	0.861
	ΔZ-score head circumference 36 weeks' PMA-DoL 14[k,ψ]	-0.90 ± 1.03*	-0.30 ± 1.11*	0.002*	-0.86 ± 1.02*	-0.30 ± 1.11*	0.005*
Postnatal variables: HD	PMA at hospital discharge, *weeks*	39.00 (5.00)*	38.00 (2.00)*	0.020*	39.00 (5.00)*	38.00 (2.00)*	0.020*

Legend: [a]According to the Bayley Scales of Infant and Toddler Development – III Edition (Bayley-III)[7]; [b]Patients with cognitive composite score < 85 or deceased between DoL 8 and the day of neurodevelopmental assessment at 24 months' CA; [c]Patients with cognitive composite score ≥ 85; [d]Patients with cognitive composite score < 85; [e]Overt diabetes is diagnosed at the first prenatal visit in case of: 1) FPG ≥ 7.0 mmol/L (126 mg/dL), or 2) HbA1c ≥ 6.5%, or 3) RPG ≥ 11.1 mmol/L (200 mg/dL); in the last case, confirmation by FPG or HbA1c is needed. Gestational DM is diagnosed in case of: 1) FPG ≥ 5.1 mmol/L (92 mg/dL) but < 7.0 mmol/L (126 mg/dL) at the first prenatal visit, or 2) pathological 2-h 75-g OGTT at 24-28 weeks' GA. The 75-g OGTT is abnormal in the following cases: FPG ≥ 5.1 mmol/L (92 mg/dL), or 1-h plasma glucose ≥ 10.0 mmol/L (180 mg/dL), or 2-h plasma glucose ≥ 8.5 mmol/L (153 mg/dL). Overt diabetes is also diagnosed in case of FPG ≥ 7.0 mmol/L (126 mg/dL) at the 75-g OGTT[8]; [f]In the present study, FGR includes both early (< 32 weeks' GA) and late cases (≥ 32 weeks' GA). According to the Delphi consensus criteria, early FGR is diagnosed in case of: 1) AC/EFW < 3rd percentile, or 2) UA-AEDF, or 3) AC/EFW < 10th percentile combined with UtA-PI > 95th percentile and/or UA-PI > 95th percentile. Late cases are defined by: AC/EFW < 3rd percentile, or at least 2 out of the following three criteria: 1) AC/EFW < 10th percentile, or 2) AC/EFW crossing percentiles > 2 quartiles on growth percentiles, or 3) CPR < 5th percentile or UA-PI > 95th percentile[9]; [g]Clinical chorioamnionitis is defined by maternal fever (temperature of 37.8°C or greater) that is not attributable to another cause along with two or more of the following conditions: 1) maternal tachycardia (HR >100 beats/min), 2) uterine tenderness, 3) foul-smelling amniotic fluid, 4) fetal tachycardia (HR >160 beats/min), and 5) maternal leucocytosis (leucocyte count >15 000 cells/mm^3). Cases of histologically-diagnosed chorioamnionitis are also included in this study[10]; [h]Intrapartum risk of infection occurs in case of: 1) previous neonate with invasive GBS disease, 2) GBS bacteriuria during any trimester of the current pregnancy, 3) positive GBS culture in the current pregnancy (unless a caesarean birth is performed before onset of labor for a woman with intact amniotic membranes), or 4) unknown GBS status at the onset of labor and any of the following: a) birth < 37$^{0/7}$ weeks' GA, b) amniotic membrane rupture ≥ 18 hours, c) intrapartum temperature ≥ 38.0°C, d) known positive GBS status in a previous pregnancy, e) intrapartum NAAT positive for GBS or negative but risk factors develop (i.e., preterm birth, amniotic membrane rupture ≥ 18 hours, or maternal temperature ≥ 38.0°C)[11]; [i]Penicillin G, ampicillin, or cefazolin; [j]According to our local guidelines, antenatal corticosteroid prophylaxis is based on intramuscular betamethasone and considered complete when two doses of 12 mg each are administered over a 24-h period; [k]Z-score values have been calculated by using the multicentre, multiethnic Intergrowth-21st curves available online at https://intergrowth21.ndog.ox.ac.uk/. Thus, obtained data account for both sex and GA at birth/PMA; [l]Indications for initiation of MV were as follows: 1) Intractable or recurrent apnoea, 2) gasping or poor respiration, 3) O_2 saturation < 90% with FiO_2 > 40%, 4) failure of non-invasive respiratory support, defined as worsening respiratory distress, respiratory acidosis (pH < 7.20 and/or $PaCO_2$ > 65 mmHg), hypoxaemia (PaO_2 < 50 mmHg), or recurrent episodes of apnoea despite PEEP ≥ 6 cmH_2O and FiO_2 > 40%; [m]Poractant alfa has been administered to all newborns < 28 weeks' GA who required intubation for stabilisation at birth and supplemental O_2 at 15 minutes of life, independently of FiO_2 provided. It has also been administered to all patients ≥ 28 weeks' GA with worsening respiratory distress despite FiO_2 > 30% on PEEP ≥ 6 cmH_2O. A second and occasionally a third dose of surfactant have been administered in case of persistent respiratory distress with need of FiO_2 > 30% once other problems have been excluded; [n]Fentanyl has been administered as intermittent, intravenous boluses of 0.5-4.0 mcg/kg/dose, or infusion of 0.5-5.0 mcg/kg/h; [o]The standard dosing regimen of caffeine citrate is loading with 20 mg/kg followed by maintenance of 5-10 mg/kg/day given by the intravenous or oral route of administration. The treatment is started to all newborns < 32 weeks' GA or undergoing MV, and in all patients with apnoeic episodes independently of GA at birth. Caffeine citrate administration is discontinued 7 days after the last episode of apnoea recorded; [p]Dexamethasone has been administered as follows: 0.15-0.20 mg/kg every 24 hours through the intravenous or oral route of administration for 5 days. The entire course of therapy is repeatable in case of necessity; [q]Diuretic therapy implies the administration of furosemide, hydrochlorothiazide, or spironolactone. The last two pharmaceuticals are usually given together during the same course of treatment. As regards furosemide, we administer 1 mg/kg/day through the intravenous or oral route in patients with GA at birth < 32 weeks; newborns with GA at birth ≥ 32 weeks receive 1 mg/kg every 12 hours through the same routes of administration. In both cases, the highest possible posology is 6 mg/kg for each dose. The dosage of furosemide for continuous infusion is 0.05-0.40 mg/kg/h. Hydrochlorothiazide is given orally at a dosage of 2.0-3.3 mg/kg every 12 hours. Similarly, we usually administer 0.5-1.5 mg/kg of spironolactone every 12 hours through the oral route; [r]In the present study, the following molecules are deemed as inotropes: dopamine, dobutamine, adrenaline, noradrenaline, and milrinone. At our institution, dosages of these molecules are the following ones: dopamine 2-10 mcg/kg/min, dobutamine 5-20 mcg/kg/min, adrenaline 0.1-1.0 mcg/kg/min, noradrenaline 0.02-0.50 mcg/kg/min, milrinone 0.75 mcg/kg/min for 3 hours followed by 0.20 mcg/kg/min; [s]According to our local guidelines, hs-PDA requiring treatment is diagnosed if both the following criteria are satisfied: 1) presence of one or more clinical signs of excessive shunting from the arterial to pulmonary circulation (e.g., continuous or systolic murmur, low diastolic blood pressure, wide pulse pressure, hypotension, bounding pulses, increased serum creatinine concentration or oliguria, hepatomegaly, pulmonary oedema, feeding intolerance); 2) presence of one or more ultrasonographic indicators: a. PDA diameter ≥ 1.5 mm (PMA ≤ 26 weeks), or ≥ 2.0 mm (PMA > 26 weeks), and growing, pulsatile shunt pattern; b. LA:AoR ratio ≥ 1.4, c. LVO ≥ 300 ml/kg/min, d. LPA EDV > 20 cm/s, e. retrograde flow in descending aorta, f. low anterograde flow in systole/diastole or absent/reversed diastolic flow in middle cerebral artery, renal artery, superior mesenteric artery, or coeliac artery[12,13]; [t]For the purposes of the present study, IVH has been classified according to Papile et al.[14]; [u]For the purposes of the present study, ROP screening was started at 31 weeks' PMA; [v]Hearing screening was based on evaluation of ABR, which was performed after 34 weeks' PMA; [w]For the purposes of the present study, BPD is defined as an ongoing need for supplemental oxygen and/or respiratory support at either 28 days' postnatal age (newborns ≥ 32 weeks' GA) or 36 weeks' PMA (infants with GA at birth < 32 weeks) in association with radiographic evidence of parenchymal lung disease[15]; [x]Clinically significant jaundice requiring treatment has been determined on the basis of total serum bilirubin values; cutoff for phototherapy and exchange transfusion have been adopted from the National Institute for Health and Clinical Excellence (NICE) 2010 guideline[16]; [y]As regards the haemoglobin cutoff values for RBC transfusion, we have adopted the low thresholds from the "Premature Infants in Need of Transfusion" (PINT) study by Kirpalani et al., even extended to VLBW infants. The volume of each RBC transfusion was 15 mL/kg[17,18]; [z]> 144 mg/dL (8.0 mmol/L)[19]; [&]< 47 mg/dL (2.6 mmol/L)[19]; [/]Simultaneous occurrence of phosphorus < 5.0 mg/dL and ionised calcium > 1.30 mmol/L[20]; [(]Serum triglyceride concentration > 265 mg/dL[21]; [)]We defined metabolic acidosis as arterial or capillary pH < 7.30 with BE_{ecf} < -6.0 mmol/L and pCO_2 ≤ 45.0 mmHg; [=]We defined metabolic alkalosis as arterial or capillary pH > 7.45 with BE_{ecf} > +4.0 mmol/L and pCO_2 ≥ 35.0 mmHg; [?]We defined respiratory acidosis as arterial or capillary pH < 7.30 with pCO_2 > 50.0 mmHg and $HCO_3^-{}_{std}$ ≥ 22.0 mEq/L; ^We defined respiratory alkalosis as arterial or capillary pH > 7.45 with pCO_2 < 35.0 mmHg and $HCO_3^-{}_{std}$ ≤ 26.0 mEq/L; [∞]Serum sodium > 145.0 mmol/L[22]; [§]Serum sodium < 133.0 mmol/L[23]; [>]Serum potassium > 6.0 mmol/L[24]; [<]Serum potassium < 3.5 mmol/L[24]; [•]Serum chlorine > 111.0 mmol/L[25]; [□]Serum chlorine < 101.0 mmol/L[25]; [◊]Total serum calcium > 11.0 mg/dL or ionised calcium > 1.50 mmol/L[19]; [Э]Total serum calcium < 7.0 mg/dL or ionised calcium < 0.90 mmol/L[24,26]; [[]Serum phosphorus > 7.5 mg/dL; []]Serum phosphorus < 4.5 mg/dL; [#]Serum magnesium > 2.2 mg/dL; [@]Serum magnesium < 1.5 mg/dL; [σ](Z-score body weight at 36 weeks' PMA – Z-score body weight at DoL 14); [ψ](Z-score HC at 36 weeks' PMA – Z-score HC at DoL 14); *p<0.05.

List of abbreviations:

ABR, auditory brainstem response; AC, fetal abdominal circumference; AEDF, absent end-diastolic flow; AoR, aortic root; BD_{ecf}, base deficit in extracellular fluid; BE_{ecf}, base excess in extracellular fluid; BPD, bronchopulmonary dysplasia; CA, corrected age; CPR, cerebroplacental ratio; DM, diabetes mellitus; DoL, day of life; EDV, end-diastolic velocity; EFW, estimated fetal weight; FGR, fetal growth restriction; FPG, fasting plasma glucose; GA, gestational age; GBS, Group B Streptococcus; HbA1c, glycated haemoglobin; HC, head circumference; $HCO_3^-{}_{std}$, standard bicarbonate ion; HD, hospital discharge; HR, heart rate; HS, hospital stay; hs-PDA, haemodynamically-significant patent ductus arteriosus; IQR, interquartile range; IVH, intraventricular haemorrhage; LA, left atrium; LPA, left pulmonary artery; LVO, left ventricular output; MV, mechanical ventilation; N, number of patients; NAAT, nucleic acid amplification test; OGTT, oral glucose tolerance test; PDA, patent ductus arteriosus; PEEP, positive end-expiratory pressure; PI, pulsatility index; PMA, postmenstrual age; RBC, red blood cell; ROP, retinopathy of prematurity; RPG, random plasma glucose; UA, umbilical artery; UtA, uterine artery; VLBW, very low birth weight; Δ, variation.

Supplementary Table S2. Complete version of Table 2: characteristics of the study population in relation to 1) the composite outcome of language development at 24 months' CA and death between DoL 8 and the neurodevelopmental assessment at 24 months' CA, and 2) language development at 24 months' CA for long-term survivors. All the variables analysed are reported, including those not reaching statistical significance. Normally-distributed continuous variables are expressed as mean ± standard deviation; non-normally distributed continuous variables are expressed as median and interquartile range.

		Language development[a] and Death			Language development[a]		
		**Cases[b]**	**Controls[c]**	**p-value**	**Cases[d]**	**Controls[c]**	**p-value**
	Patients, *N (%)*	78 (53.42)	68 (46.58)	-	67 (49.63)	68 (50.37)	-
	Score, *median (IQR)*	77.00 (11.00)*	91.00 (7.25)*	< 0.001*	77.00 (11.00)*	91.00 (7.25)*	< 0.001*
Prenatal variables: maternal data	Maternal age, *years*	33.00 (8.00)	35.00 (9.00)	0.465	33.00 (8.00)	35.00 (9.00)	0.558
	Maternal ethnicity: Caucasian, *N (%)*	66/78 (84.61)	62/68 (91.18)	0.229	57/67 (85.07)	62/68 (91.18)	0.273
	Maternal high school diploma or higher educational level, *N (%)*	57/78 (73.08)	53/68 (77.94)	0.496	51/67 (76.12)	53/68 (77.94)	0.801
	Maternal university degree or higher educational level, *N (%)*	20/78 (25.64)	22/68 (32.35)	0.371	20/67 (29.85)	22/68 (32.35)	0.754
Prenatal variables: pregnancy data	Maternal smoking during pregnancy, *N (%)*	20/78 (25.64)	9/68 (13.23)	0.061	17/67 (25.37)	9/68 (13.23)	0.074
	Maternal alcohol consumption during pregnancy, *N (%)*	2/78 (2.56)	0/68 (0.00)	0.499	2/67 (2.98)	0/68 (0.00)	0.244
	Maternal drug use during pregnancy, *N (%)*	1/78 (1.28)	0/68 (0.00)	1.000	1/67 (1.49)	0/68 (0.00)	0.496
	Maternal thyroid disorders during pregnancy, *N (%)*	7/78 (8.97)	11/68 (16.18)	0.187	6/67 (8.95)	11/68 (16.18)	0.206
	Maternal diabetes during pregnancy[e], *N (%)*	9/78 (11.54)	10/68 (14.71)	0.570	5/67 (7.46)	10/68 (14.71)	0.181
	Intrauterine growth restriction[f], *N (%)*	11/78 (14.10)	10/68 (14.71)	0.917	10/67 (14.92)	10/68 (14.71)	0.971
	Maternal pressure disorders during pregnancy, *N (%)*	17/78 (21.79)	22/68 (32.35)	0.150	16/67 (23.88)	22/68 (32.35)	0.274
	Alterations of Doppler flow velocimetry, *N (%)*	11/78 (14.10)	18/68 (26.47)	0.062	11/67 (16.42)	18/68 (26.47)	0.155
Perinatal variables: data about delivery and patient in the first hour of life	Placental abruption, *N (%)*	8/78 (10.26)	3/68 (4.41)	0.182	5/67 (7.46)	3/68 (4.41)	0.493
	Chorioamnionitis[g], *N (%)*	10/78 (12.82)	3/68 (4.41)	0.075	10/67 (14.92)*	3/68 (4.41)*	0.038*
	One or more risk factors for intrapartum infection[h], *N (%)*	47/78 (60.26)	41/68 (60.29)	0.996	37/67 (55.22)	41/68 (60.29)	0.551

	Intrapartum antibiotics ≥ 4 hours before birth[i], *N (%)*	22/78 (28.20)	13/68 (19.12)	0.199	19/67 (28.36)	13/68 (19.12)	0.207
	Antenatal corticosteroids[j], *number of doses*	1.00 (2.00)	2.00 (1.00)	0.101	2.00 (1.00)	2.00 (1.00)	0.731
	Caesarean section, *N (%)*	63/78 (80.77)	62/68 (91.18)	0.074	55/67 (82.09)	62/68 (91.18)	0.120
	Male sex, *N (%)*	47/78 (60.26)	30/68 (44.12)	0.051	41/67 (61.19)*	30/68 (44.12)*	0.047*
	Gestational age, *weeks*	29.00 (4.00)*	30.00 (4.00)*	0.003*	29.34 ± 2.23*	30.15 ± 2.34*	0.043*
	Birth weight, *g*	1128.00 (575.00)	1253.50 (417.50)	0.050	1225.00 (538.00)	1253.50 (417.50)	0.335
	Birth weight Z-score[k]	0.03 (1.59)	-0.46 (1.88)	0.558	0.03 (1.65)	-0.46 (1.88)	0.621
	Head circumference, *cm*	26.90 (4.12)	27.95 (2.38)	0.054	27.00 (4.00)	27.95 (2.38)	0.196
	Head circumference Z-score[k]	0.14 (1.37)	-0.06 (1.48)	0.298	0.11 (1.33)	-0.06 (1.48)	0.379
	Twin infants, *N (%)*	22/78 (28.20)	15/68 (22.06)	0.394	21/67 (31.34)	15/68 (22.06)	0.223
	5-minute Apgar score	8.00 (1.00)	8.00 (2.00)	0.285	8.00 (1.00)	8.00 (2.00)	0.842
	Umbilical cord arterial pH	7.26 (0.09)	7.26 (0.08)	0.976	7.27 (0.08)	7.26 (0.08)	0.511
	Umbilical cord arterial BD_{ecf}	-5.65 (4.77)	-5.40 (4.70)	0.505	-4.90 (4.40)	-5.40 (4.70)	0.947
	Body temperature at 1 hour of life, *°C*	36.10 (0.50)	36.10 (0.50)	0.694	36.10 (0.40)	36.10 (0.50)	0.446
Postnatal variables: ventilation data	Invasive mechanical ventilation[l], *N (%)*	32/78 (41.03)*	16/68 (23.53)*	0.025*	21/67 (31.34)	16/68 (23.53)	0.309
	Surfactant[m], *number of doses*	1.00 (1.00)	1.00 (1.00)	0.554	1.00 (1.00)	1.00 (1.00)	0.983
Postnatal variables: medications	Duration of fentanyl administration[n], *days*	0.00 (1.00)	0.00 (0.00)	0.201	0.00 (0.00)	0.00 (0.00)	0.704
	Duration of caffeine administration[o], *days*	22.00 (14.00)	21.50 (14.00)	0.837	22.00 (14.00)	21.50 (14.00)	0.895
	Duration of postnatal corticosteroid therapy[p], *days*	0.00 (0.00)	0.00 (0.00)	0.059	0.00 (0.00)	0.00 (0.00)	0.195
	Duration of diuretic therapy[q], *days*	0.00 (0.00)	0.00 (0.00)	0.356	0.00 (0.00)	0.00 (0.00)	0.753
	Duration of antibiotic prophylaxis/therapy, *days*	7.00 (5.75)*	5.00 (4.00)*	< 0.001*	7.00 (5.00)*	5.00 (4.00)*	0.002*
	Duration of inotropic support[r], *days*	0.00 (0.00)*	0.00 (0.00)*	0.014*	0.00 (0.00)	0.00 (0.00)	0.509

Postnatal variables: morbidities	Haemodynamically significant patent ductus arteriosus[s], *N (%)*	20/78 (25.64)	16/68 (23.53)	0.768	12/67 (17.91)	16/68 (23.53)	0.421
	Necrotising enterocolitis (any stage), *N (%)*	7/78 (8.97)	3/68 (4.41)	0.339	3/67 (4.48)	3/68 (4.41)	1.000
	Intraventricular haemorrhage grade III-IV[t], *N (%)*	18/78 (23.08)*	1/68 (1.47)*	<0.001*	8/67 (11.94)*	1/68 (1.47)*	0.017*
	Electrical-only and/or electroclinical seizures, *N (%)*	2/78 (2.56)	0/68 (0.00)	0.499	2/67 (2.98)	0/68 (0.00)	0.244
	Early-onset and/or late-onset sepsis, *N (%)*	19/78 (24.36)*	3/68 (4.41)*	<0.001*	9/67 (13.43)	3/68 (4.41)	0.066
	Retinopathy of prematurity (any stage)[u], *N (%)*	19/72 (26.39)*	8/68 (11.76)*	0.028*	19/67 (28.36)*	8/68 (11.76)*	0.016*
	Congenital hearing loss[v], *N (%)*	1/70 (1.43)	0/68 (0.00)	1.000	1/67 (1.49)	0/68 (0.00)	0.496
	Bronchopulmonary dysplasia[w], *N (%)*	7/69 (10.14)	1/68 (1.47)	0.062	5/67 (7.46)	1/68 (1.47)	0.115
	Jaundice requiring phototherapy and/or exchange transfusion[x], *N (%)*	54/78 (69.23)	46/68 (67.65)	0.837	50/67 (74.63)	46/68 (67.65)	0.371
	Anaemia requiring one or more red blood cell transfusions[y], *N (%)*	33/78 (42.31)*	12/68 (17.65)*	0.001*	23/67 (34.33)*	12/68 (17.65)*	0.027*
Postnatal variables: metabolic disorders	Hyperglycaemia during HS[z], *N (%)*	17/78 (21.79)*	3/68 (4.41)*	0.002*	10/67 (14.92)*	3/68 (4.41)*	0.038*
	Hypoglycaemia during HS[&], *N (%)*	15/78 (19.23)	6/68 (8.82)	0.074	11/67 (16.42)	6/68 (8.82)	0.184
	Hypophosphataemic hypercalcaemia during HS[/], *N (%)*	12/78 (15.38)	10/68 (14.71)	0.909	10/67 (14.92)	10/68 (14.71)	0.971
	Hypertriglyceridaemia during HS[(], *N (%)*	8/78 (10.26)	5/68 (7.35)	0.539	6/67 (8.95)	5/68 (7.35)	0.734
Postnatal variables: acid-base disorders	Metabolic acidosis during HS[)], *N (%)*	23/78 (29.49)*	7/68 (10.29)*	0.004*	13/67 (19.40)	7/68 (10.29)	0.136
	Metabolic alkalosis during HS[=], *N (%)*	1/78 (1.28)	0/68 (0.00)	1.000	1/67 (1.49)	0/68 (0.00)	0.496
	Respiratory acidosis during HS[?], *N (%)*	9/78 (11.54)	8/68 (11.76)	0.966	6/67 (8.95)	8/68 (11.76)	0.592
	Respiratory alkalosis during HS^, *N (%)*	6/78 (7.69)	2/68 (2.94)	0.285	6/67 (8.95)	2/68 (2.94)	0.165
Postnatal variables: electrolyte disturbances	Hypernatraemia during HS[∞], *N (%)*	17/78 (21.79)	7/68 (10.29)	0.061	10/67 (14.92)	7/68 (10.29)	0.417
	Hyponatraemia during HS[§], *N (%)*	42/78 (53.85)	26/68 (38.23)	0.059	36/67 (53.73)	26/68 (38.23)	0.071
	Hyperkalaemia during HS [>], *N (%)*	21/78 (26.92)	12/68 (17.65)	0.181	15/67 (22.39)	12/68 (17.65)	0.491
	Hypokalaemia during HS[<], *N (%)*	5/78 (6.41)	4/68 (5.88)	1.000	3/67 (4.48)	4/68 (5.88)	1.000

	Hyperchloraemia during HS[•], *N (%)*	43/78 (55.13)	28/68 (41.18)	0.092	36/67 (53.73)	28/68 (41.18)	0.144
	Hypochloraemia during HS[□], *N (%)*	8/78 (10.26)	9/68 (13.23)	0.576	8/67 (11.94)	9/68 (13.23)	0.821
	Hypercalcaemia during HS[◊], *N (%)*	17/78 (21.79)	10/68 (14.71)	0.271	16/67 (23.88)	10/68 (14.71)	0.177
	Hypocalcaemia during HS[Ͽ], *N (%)*	13/78 (16.67)	10/68 (14.71)	0.746	9/67 (13.43)	10/68 (14.71)	0.832
	Hyperphosphataemia during HS[⌈], *N (%)*	16/78 (20.51)	17/68 (25.00)	0.518	11/67 (16.42)	17/68 (25.00)	0.219
	Hypophosphataemia during HS[⌉], *N (%)*	16/78 (20.51)	15/68 (22.06)	0.820	14/67 (20.89)	15/68 (22.06)	0.869
	Hypermagnesaemia during HS[#], *N (%)*	32/78 (41.03)	24/68 (35.29)	0.477	23/67 (34.33)	24/68 (35.29)	0.906
	Hypomagnesaemia during HS[@], *N (%)*	8/78 (10.26)	5/68 (7.35)	0.539	8/67 (11.94)	5/68 (7.35)	0.366
Postnatal variables: nutritional data	Start of enteral nutrition, *days of life*	2.00 (4.00)*	1.00 (1.00)*	0.014*	1.00 (3.00)	1.00 (1.00)	0.111
	Maternal milk intake DoL 0-7, *ml/kg/first week of life*	12.13 (74.91)*	43.24 (263.23)*	0.023*	13.15 (81.88)	43.24 (263.23)	0.070
	Total milk intake DoL 0-7, *ml/kg/first week of life*	83.22 (255.04)*	234.51 (411.17)*	0.002*	109.28 (284.39)*	234.51 (411.17)*	0.016*
	Maternal milk intake DoL 0-7/Total milk intake DoL 0-7, *%*	29.96 (67.60)	35.34 (77.60)	0.373	29.87 (64.78)	35.34 (77.60)	0.318
	Duration of parenteral nutrition, *days*	13.50 (15.00)*	8.00 (10.00)*	0.001*	14.00 (15.00)*	8.00 (10.00)*	0.006*
Postnatal variables: body growth	Postnatal weight loss, *%*	-12.72 (6.37)	-11.01 (5.22)	0.055	-12.29 ± 4.51	-11.15 ± 4.36	0.138
	Recovery of birth weight, *DoL*	12.00 (4.75)	12.00 (4.00)	0.965	12.00 (5.00)	12.00 (4.00)	0.924
	Body weight at DoL 14 Z-Score[k]	-1.01 (1.49)	-1.46 (1.57)	0.719	-1.00 (1.45)	-1.46 (1.57)	0.591
	Head circumference at DoL 14 Z-Score[k]	-0.13 (1.50)	-0.36 (1.64)	0.529	-0.14 (1.03)	-0.36 (1.64)	0.499
	Body weight at 36 weeks' PMA Z-Score[k]	-1.43 (1.80)	-1.86 (1.76)	0.395	-1.43 (1.73)	-1.86 (1.76)	0.399
	Head circumference at 36 weeks' PMA Z-Score[k]	-1.07 (1.77)	-0.54 (2.15)	0.246	-1.07 (1.72)	-0.54 (2.15)	0.317
	ΔZ-score body weight 36 weeks' PMA-DoL 14[k,σ]	-0.10 (0.70)	-0.20 (0.58)	0.433	-0.10 (0.66)	-0.20 (0.58)	0.406
	ΔZ-score head circumference 36 weeks' PMA-DoL 14[k,ψ]	-0.71 ± 1.11	-0.38 ± 1.11	0.087	-0.67 ± 1.10	-0.38 ± 1.11	0.133
Postnatal variables: HD	PMA at hospital discharge, *weeks*	38.00 (5.00)*	38.00 (2.00)*	0.033*	38.00 (5.00)*	38.00 (2.00)*	0.033*

For legend and list of abbreviations, see Supplementary Table S1.

Supplementary Table S3. Complete version of Table 3: characteristics of the study population in relation to 1) the composite outcome of motor development at 24 months' CA and death between DoL 8 and the neurodevelopmental assessment at 24 months' CA, and 2) motor development at 24 months' CA for long-term survivors. All the variables analysed are reported, including those not reaching statistical significance. Normally-distributed continuous variables are expressed as mean ± standard deviation; non-normally distributed continuous variables are expressed as median and interquartile range.

		**Motor development[a] and Death**			**Motor development[a]**		
		**Cases[b]**	**Controls[c]**	**p-value**	**Cases[d]**	**Controls[c]**	**p-value**
	Patients, *N (%)*	45 (30.82)	101 (69.18)	-	34 (25.18)	101 (74.81)	-
	Score, *median (IQR)*	79.00 (12.00)*	97.00 (10.50)*	< 0.001*	79.00 (12.00)*	97.00 (10.50)*	< 0.001*
Prenatal variables: maternal data	Maternal age, *years*	33.58 ± 5.85	34.58 ± 6.10	0.353	33.59 ± 6.27	34.58 ± 6.10	0.415
	Maternal ethnicity: Caucasian, *N (%)*	38/45 (84.44)	90/101 (89.11)	0.429	29/34 (85.29)	90/101 (89.11)	0.549
	Maternal high school diploma or higher educational level, *N (%)*	33/45 (73.33)	77/101 (76.24)	0.707	27/34 (79.41)	77/101 (76.24)	0.703
	Maternal university degree or higher educational level, *N (%)*	11/45 (24.44)	31/101 (30.69)	0.441	11/34 (32.35)	31/101 (30.69)	0.856
Prenatal variables: pregnancy data	Maternal smoking during pregnancy, *N (%)*	12/45 (26.67)	17/101 (16.83)	0.169	9/34 (26.47)	17/101 (16.83)	0.218
	Maternal alcohol consumption during pregnancy, *N (%)*	2/45 (4.44)	0/101 (0.00)	0.094	2/34 (5.88)	0/101 (0.00)	0.062
	Maternal drug use during pregnancy, *N (%)*	1/45 (2.22)	0/101 (0.00)	0.308	1/34 (2.94)	0/101 (0.00)	0.252
	Maternal thyroid disorders during pregnancy, *N (%)*	4/45 (8.89)	14/101 (13.86)	0.399	3/34 (8.82)	14/101 (13.86)	0.560
	Maternal diabetes during pregnancy[e], *N (%)*	9/45 (20.00)	10/101 (9.90)	0.094	5/34 (14.71)	10/101 (9.90)	0.528
	Intrauterine growth restriction[f], *N (%)*	8/45 (17.78)	13/101 (12.87)	0.435	7/34 (20.59)	13/101 (12.87)	0.273
	Maternal pressure disorders during pregnancy, *N (%)*	10/45 (22.22)	29/101 (28.71)	0.413	9/34 (26.47)	29/101 (28.71)	0.801
	Alterations of Doppler flow velocimetry, *N (%)*	8/45 (17.78)	21/101 (20.79)	0.673	8/34 (23.53)	21/101 (20.79)	0.737
Perinatal variables: data about delivery and patient in the first hour of life	Placental abruption, *N (%)*	5/45 (11.11)	6/101 (5.94)	0.315	2/34 (5.88)	6/101 (5.94)	1.000
	Chorioamnionitis[g], *N (%)*	6/45 (13.33)	7/101 (6.93)	0.221	6/34 (17.65)	7/101 (6.93)	0.091
	One or more risk factors for intrapartum infection[h], *N (%)*	27/45 (60.00)	61/101 (60.40)	0.964	17/34 (50.00)	61/101 (60.40)	0.288

	Intrapartum antibiotics ≥ 4 hours before birth[i], *N (%)*	12/45 (26.67)	23/101 (22.77)	0.611	9/34 (26.47)	23/101 (22.77)	0.661
	Antenatal corticosteroids[j], *number of doses*	1.00 (2.00)*	2.00 (1.00)*	0.005*	1.00 (1.00)	2.00 (1.00)	0.407
	Caesarean section, *N (%)*	35/45 (77.78)	90/101 (89.11)	0.072	27/34 (79.41)	90/101 (89.11)	0.156
	Male sex, *N (%)*	27/45 (60.00)	50/101 (49.50)	0.241	21/34 (61.76)	50/101 (49.50)	0.216
	Gestational age, *weeks*	28.00 (4.00)*	30.00 (3.00)*	< 0.001*	29.00 (4.00)*	30.00 (3.00)*	0.002*
	Birth weight, *g*	930.00 (526.50)*	1285.00 (391.00)*	< 0.001*	975.00 (522.50)*	1285.00 (391.00)*	< 0.001*
	Birth weight Z-score[k]	0.06 (1.91)	-0.06 (1.69)	0.695	0.05 (2.06)	-0.06 (1.69)	0.542
	Head circumference, *cm*	25.72 ± 2.85*	27.64 ± 1.89*	< 0.001*	25.75 (4.13)*	27.80 (2.50)*	< 0.001*
	Head circumference Z-score[k]	-0.03 (1.89)	0.09 (1.18)	0.790	-0.22 ± 1.13	-0.05 ± 1.04	0.418
	Twin infants, *N (%)*	11/45 (24.44)	26/101 (25.74)	0.868	10/34 (29.41)	26/101 (25.74)	0.676
	5-minute Apgar score	8.00 (1.00)	8.00 (1.00)	0.072	8.00 (2.00)	8.00 (1.00)	0.798
	Umbilical cord arterial pH	7.29 (0.10)	7.26 (0.08)	0.603	7.29 (0.08)	7.26 (0.08)	0.091
	Umbilical cord arterial BD_{ecf}	-5.50 (5.70)	-5.40 (4.40)	0.499	-4.65 (5.03)	-5.40 (4.40)	0.708
	Body temperature at 1 hour of life, *°C*	36.30 (0.50)	36.10 (0.50)	0.486	36.20 (0.40)	36.10 (0.50)	0.832
Postnatal variables: ventilation data	Invasive mechanical ventilation[l], *N (%)*	27/45 (60.00)*	21/101 (20.79)*	< 0.001*	16/34 (47.06)*	21/101 (20.79)*	0.003*
	Surfactant[m], *number of doses*	1.00 (2.00)	0.00 (1.00)	0.078	1.00 (1.00)	0.00 (1.00)	0.392
Postnatal variables: medications	Duration of fentanyl administration[n], *days*	0.00 (6.00)*	0.00 (0.00)*	< 0.001*	0.00 (1.00)	0.00 (0.00)	0.085
	Duration of caffeine administration[o], *days*	22.00 (19.00)	21.00 (14.00)	0.277	23.50 (14.00)	21.00 (14.00)	0.285
	Duration of postnatal corticosteroid therapy[p], *days*	0.00 (0.00)*	0.00 (0.00)*	0.018*	0.00 (0.00)	0.00 (0.00)	0.148
	Duration of diuretic therapy[q], *days*	0.00 (0.00)	0.00 (0.00)	0.083	0.00 (0.00)	0.00 (0.00)	0.403
	Duration of antibiotic prophylaxis/therapy, *days*	10.00 (11.00)*	5.00 (4.00)*	< 0.001*	9.00 (11.50)*	5.00 (4.00)*	0.013*
	Duration of inotropic support[r], *days*	0.00 (3.00)*	0.00 (0.00)*	< 0.001*	0.00 (0.00)*	0.00 (0.00)*	0.004*

Postnatal variables: morbidities	Haemodynamically significant patent ductus arteriosus[s], *N (%)*	15/45 (33.33)	21/101 (20.79)	0.104	7/34 (20.59)	21/101 (20.79)	0.980
	Necrotising enterocolitis (any stage), *N (%)*	4/45 (8.89)	6/101 (5.94)	0.498	0/34 (0.00)	6/101 (5.94)	0.337
	Intraventricular haemorrhage grade III-IV[t], *N (%)*	13/45 (28.89)*	6/101 (5.94)*	<0.001*	3/34 (8.82)	6/101 (5.94)	0.691
	Electrical-only and/or electroclinical seizures, *N (%)*	2/45 (4.44)	0/101 (0.00)	0.094	2/34 (5.88)	0/101 (0.00)	0.062
	Early-onset and/or late-onset sepsis, *N (%)*	20/45 (44.44)*	2/101 (1.98)*	<0.001*	10/34 (29.41)*	2/101 (1.98)*	<0.001*
	Retinopathy of prematurity (any stage)[u], *N (%)*	14/39 (35.90)*	13/101 (12.87)*	0.002*	14/34 (41.18)*	13/101 (12.87)*	<0.001*
	Congenital hearing loss[v], *N (%)*	0/37 (0.00)	1/101 (0.99)	1.000	0/34 (0.00)	1/101 (0.99)	1.000
	Bronchopulmonary dysplasia[w], *N (%)*	8/36 (22.22)*	0/101 (0.00)*	<0.001*	6/34 (17.65)*	0/101 (0.00)*	<0.001*
	Jaundice requiring phototherapy and/or exchange transfusion[x], *N (%)*	31/45 (68.89)	69/101 (68.32)	0.945	27/34 (79.41)	69/101 (68.32)	0.217
	Anaemia requiring one or more red blood cell transfusions[y], *N (%)*	29/45 (64.44)*	16/101 (15.84)*	<0.001*	19/34 (55.88)*	16/101 (15.84)*	<0.001*
Postnatal variables: metabolic disorders	Hyperglycaemia during HS[z], *N (%)*	14/45 (31.11)*	6/101 (5.94)*	<0.001*	7/34 (20.59)*	6/101 (5.94)*	0.019*
	Hypoglycaemia during HS[&], *N (%)*	9/45 (20.00)	12/101 (11.88)	0.197	5/34 (14.71)	12/101 (11.88)	0.766
	Hypophosphataemic hypercalcaemia during HS[/], *N (%)*	10/45 (22.22)	12/101 (11.88)	0.107	8/34 (23.53)	12/101 (11.88)	0.098
	Hypertriglyceridaemia during HS[(], *N (%)*	7/45 (15.56)	6/101 (5.94)	0.111	5/34 (14.71)	6/101 (5.94)	0.144
Postnatal variables: acid-base disorders	Metabolic acidosis during HS[)], *N (%)*	20/45 (44.44)*	10/101 (9.90)*	<0.001*	10/34 (29.41)*	10/101 (9.90)*	0.006*
	Metabolic alkalosis during HS[=], *N (%)*	1/45 (2.22)	0/101 (0.00)	0.308	1/34 (2.94)	0/101 (0.00)	0.252
	Respiratory acidosis during HS[?], *N (%)*	8/45 (17.78)	9/101 (8.91)	0.123	5/34 (14.71)	9/101 (8.91)	0.341
	Respiratory alkalosis during HS^, *N (%)*	1/45 (2.22)	7/101 (6.93)	0.435	1/34 (2.94)	7/101 (6.93)	0.679
Postnatal variables: electrolyte disturbances	Hypernatraemia during HS[∞], *N (%)*	10/45 (22.22)	14/101 (13.86)	0.208	3/34 (8.82)	14/101 (13.86)	0.560
	Hyponatraemia during HS[§], *N (%)*	26/45 (57.78)	42/101 (41.58)	0.070	20/34 (58.82)	42/101 (41.58)	0.081
	Hyperkalaemia during HS[>], *N (%)*	13/45 (28.89)	20/101 (19.80)	0.225	7/34 (20.59)	20/101 (19.80)	0.921
	Hypokalaemia during HS[<], *N (%)*	4/45 (8.89)	5/101 (4.95)	0.458	2/34 (5.88)	5/101 (4.95)	1.000

	Hyperchloraemia during HS[•], *N (%)*	23/45 (51.11)	48/101 (47.52)	0.689	16/34 (47.06)	48/101 (47.52)	0.962
	Hypochloraemia during HS[□], *N (%)*	6/45 (13.33)	11/101 (10.89)	0.671	6/34 (17.65)	11/101 (10.89)	0.370
	Hypercalcaemia during HS[◊], *N (%)*	11/45 (24.44)	16/101 (15.84)	0.216	10/34 (29.41)	16/101 (15.84)	0.083
	Hypocalcaemia during HS[Ͽ], *N (%)*	11/45 (24.44)	12/101 (11.88)	0.054	7/34 (20.59)	12/101 (11.88)	0.254
	Hyperphosphataemia during HS[⌈], *N (%)*	10/45 (22.22)	23/101 (22.77)	0.942	5/34 (14.71)	23/101 (22.77)	0.316
	Hypophosphataemia during HS[⌉], *N (%)*	13/45 (28.89)	18/101 (17.82)	0.131	11/34 (32.35)	18/101 (17.82)	0.074
	Hypermagnesaemia during HS[#], *N (%)*	21/45 (46.67)	35/101 (34.65)	0.168	12/34 (35.29)	35/101 (34.65)	0.946
	Hypomagnesaemia during HS[@], *N (%)*	2/45 (4.44)	11/101 (10.89)	0.345	2/34 (5.88)	11/101 (10.89)	0.516
Postnatal variables: nutritional data	Start of enteral nutrition, *days of life*	2.00 (5.75)*	1.00 (1.00)*	0.006*	1.00 (4.00)	1.00 (1.00)	0.205
	Maternal milk intake DoL 0-7, *ml/kg/first week of life*	10.60 (44.64)*	24.33 (187.46)*	0.037*	12.13 (60.59)	24.33 (187.46)	0.173
	Total milk intake DoL 0-7, *ml/kg/first week of life*	31.93 (187.82)*	223.33 (370.43)*	< 0.001*	38.67 (235.57)*	223.33 (370.43)*	< 0.001*
	Maternal milk intake DoL 0-7/Total milk intake DoL 0-7, *%*	39.45 (72.82)	29.34 (68.91)	0.135	35.66 (68.85)	29.34 (68.91)	0.135
	Duration of parenteral nutrition, *days*	17.00 (18.00)*	9.00 (10.00)*	< 0.001*	17.00 (19.00)*	9.00 (10.00)*	< 0.001*
Postnatal variables: body growth	Postnatal weight loss, *%*	-14.08 ± 5.78*	-11.21 ± 4.20*	0.004*	-13.21 ± 4.90*	-11.21 ± 4.20*	0.023*
	Recovery of birth weight, *DoL*	12.00 (7.00)	12.00 (4.00)	0.327	13.00 (7.25)	12.00 (4.00)	0.358
	Body weight at DoL 14 Z-Score[k]	-1.51 (1.81)	-1.06 (1.57)	0.142	-1.48 (1.54)	-1.06 (1.57)	0.182
	Head circumference at DoL 14 Z-Score[k]	-0.24 (1.98)	-0.33 (1.46)	0.766	-0.43 ± 1.15	-0.33 ± 1.07	0.622
	Body weight at 36 weeks' PMA Z-Score[k]	-1.95 (2.01)	-1.43 (1.77)	0.056	-1.95 (2.00)*	-1.43 (1.77)*	0.047*
	Head circumference at 36 weeks' PMA Z-Score[k]	-1.65 (1.66)*	-0.46 (1.75)*	< 0.001*	-1.57 (1.67)*	-0.46 (1.75)*	0.001*
	ΔZ-score body weight 36 weeks' PMA-DoL 14[k,σ]	-0.15 (0.93)	-0.17 (0.58)	0.654	-0.15 (0.86)	-0.17 (0.58)	0.682
	ΔZ-score head circumference 36 weeks' PMA-DoL 14[k,ψ]	-1.07 ± 1.10*	-0.36 ± 1.06*	< 0.001*	-1.02 ± 1.10*	-0.36 ± 1.06*	0.002*
Postnatal variables: HD	PMA at hospital discharge, *weeks*	40.00 (5.00)*	38.00 (2.00)*	< 0.001*	40.00 (5.00)*	38.00 (2.00)*	< 0.001*

For legend and list of abbreviations, see Supplementary Table S1.

Supplementary Table S4. Complete version of Table 4: characteristics of the study population in relation to 1) the composite outcome of socio-emotional development at 24 months' CA and death between DoL 8 and the neurodevelopmental assessment at 24 months' CA, and 2) socio-emotional development at 24 months' CA for long-term survivors. All the variables analysed are reported, including those not reaching statistical significance. Normally-distributed continuous variables are expressed as mean ± standard deviation; non-normally distributed continuous variables are expressed as median and interquartile range.

		**Socio-emotional development[a] and Death**			**Socio-emotional development[a]**		
		**Cases[b]**	**Controls[c]**	**p-value**	**Cases[d]**	**Controls[c]**	**p-value**
	Patients, *N (%)*	62 (42.47)	84 (57.53)	-	51 (37.78)	84 (62.22)	-
	Score, *median (IQR)*	80.00 (9.00)*	105.00 (15.00)*	< 0.001*	80.00 (9.00)*	105.00 (15.00)*	< 0.001*
Prenatal variables: maternal data	Maternal age, *years*	32.50 (8.00)	35.00 (8.00)	0.774	34.55 ± 7.01	34.20 ± 5.57	0.751
	Maternal ethnicity: Caucasian, *N (%)*	55/62 (88.71)	73/84 (86.90)	0.743	46/51 (90.20)	73/84 (86.90)	0.566
	Maternal high school diploma or higher educational level, *N (%)*	45/62 (72.58)	65/84 (77.38)	0.506	39/51 (76.47)	65/84 (77.38)	0.903
	Maternal university degree or higher educational level, *N (%)*	13/62 (20.97)	29/84 (34.52)	0.074	13/51 (25.49)	29/84 (34.52)	0.272
Prenatal variables: pregnancy data	Maternal smoking during pregnancy, *N (%)*	17/62 (27.42)*	12/84 (14.29)*	0.049*	14/51 (27.45)	12/84 (14.29)	0.060
	Maternal alcohol consumption during pregnancy, *N (%)*	1/62 (1.61)	1/84 (1.19)	1.000	1/51 (1.96)	1/84 (1.19)	1.000
	Maternal drug use during pregnancy, *N (%)*	1/62 (1.61)	0/84 (0.00)	0.425	1/51 (1.96)	0/84 (0.00)	0.378
	Maternal thyroid disorders during pregnancy, *N (%)*	6/62 (9.68)	12/84 (14.29)	0.402	5/51 (9.80)	12/84 (14.29)	0.447
	Maternal diabetes during pregnancy[e], *N (%)*	7/62 (11.29)	12/84 (14.29)	0.595	3/51 (5.88)	12/84 (14.29)	0.132
	Intrauterine growth restriction[f], *N (%)*	6/62 (9.68)	15/84 (17.86)	0.164	5/51 (9.80)	15/84 (17.86)	0.202
	Maternal pressure disorders during pregnancy, *N (%)*	12/62 (19.35)	27/84 (32.14)	0.084	11/51 (21.57)	27/84 (32.14)	0.185
	Alterations of Doppler flow velocimetry, *N (%)*	10/62 (16.13)	19/84 (22.62)	0.331	10/51 (19.61)	19/84 (22.62)	0.680
Perinatal variables: data about delivery and patient in the first hour of life	Placental abruption, *N (%)*	5/62 (8.06)	6/84 (7.14)	1.000	2/51 (3.92)	6/84 (7.14)	0.710
	Chorioamnionitis[g], *N (%)*	7/62 (11.29)	6/84 (7.14)	0.384	7/51 (13.72)	6/84 (7.14)	0.238
	One or more risk factors for intrapartum infection[h], *N (%)*	35/62 (56.45)	53/84 (63.09)	0.417	25/51 (49.02)	53/84 (63.09)	0.108

	Intrapartum antibiotics ≥ 4 hours before birth[i], *N (%)*	15/62 (24.19)	20/84 (23.81)	0.957	12/51 (23.53)	20/84 (23.81)	0.970
	Antenatal corticosteroids[j], *number of doses*	1.00 (1.25)	2.00 (1.00)	0.154	2.00 (1.00)	2.00 (1.00)	0.829
	Caesarean section, *N (%)*	50/62 (80.64)	75/84 (89.29)	0.141	42/51 (82.35)	75/84 (89.29)	0.251
	Male sex, *N (%)*	39/62 (62.90)*	38/84 (45.24)*	0.035*	33/51 (64.71)*	38/84 (45.24)*	0.028*
	Gestational age, *weeks*	29.00 (4.00)*	30.00 (3.00)*	0.004*	29.00 (3.00)	30.00 (3.00)	0.109
	Birth weight, *g*	1192.50 (610.00)	1247.50 (450.25)	0.075	1245.00 (505.00)	1247.50 (450.25)	0.610
	Birth weight Z-score[k]	0.08 (1.47)	-0.41 (1.90)	0.255	0.07 (1.49)	-0.41 (1.90)	0.287
	Head circumference, *cm*	26.53 ± 2.74*	27.42 ± 2.03*	0.032*	27.50 (4.10)	27.75 (3.30)	0.301
	Head circumference Z-score[k]	0.23 (1.39)	-0.05 (1.39)	0.310	0.23 (1.32)	-0.05 (1.39)	0.409
	Twin infants, *N (%)*	19/62 (30.64)	18/84 (21.43)	0.206	18/51 (35.29)	18/84 (21.43)	0.077
	5-minute Apgar score	8.00 (1.00)	8.00 (1.00)	0.619	8.00 (1.00)	8.00 (1.00)	0.071
	Umbilical cord arterial pH	7.27 (0.12)	7.26 (0.07)	0.604	7.29 (0.10)	7.26 (0.07)	0.166
	Umbilical cord arterial BD_{ecf}	-5.45 (4.65)	-5.40 (4.73)	0.854	-4.90 (4.30)	-5.40 (4.73)	0.521
	Body temperature at 1 hour of life, *°C*	36.10 (0.50)	36.20 (0.50)	0.829	36.10 (0.40)	36.20 (0.50)	0.504
Postnatal variables: ventilation data	Invasive mechanical ventilation[l], *N (%)*	27/62 (43.55)*	21/84 (25.00)*	0.018*	16/51 (31.37)	21/84 (25.00)	0.421
	Surfactant[m], *number of doses*	1.00 (1.00)	1.00 (1.00)	0.679	0.00 (1.00)	1.00 (1.00)	0.216
Postnatal variables: medications	Duration of fentanyl administration[n], *days*	0.00 (2.25)	0.00 (0.00)	0.079	0.00 (0.00)	0.00 (0.00)	0.749
	Duration of caffeine administration[o], *days*	22.50 (15.00)	21.00 (13.00)	0.593	23.00 (14.00)	21.00 (13.00)	0.615
	Duration of postnatal corticosteroid therapy[p], *days*	0.00 (0.00)	0.00 (0.00)	0.696	0.00 (0.00)	0.00 (0.00)	0.135
	Duration of diuretic therapy[q], *days*	0.00 (0.00)	0.00 (0.00)	0.986	0.00 (0.00)	0.00 (0.00)	0.356
	Duration of antibiotic prophylaxis/therapy, *days*	7.00 (6.25)*	5.00 (7.00)*	0.011*	5.00 (7.00)	5.00 (7.00)	0.128
	Duration of inotropic support[r], *days*	0.00 (1.00)*	0.00 (0.00)*	0.002*	0.00 (0.00)	0.00 (0.00)	0.358

Postnatal variables: morbidities	Haemodynamically significant patent ductus arteriosus[s], *N (%)*	16/62 (25.81)	20/84 (23.81)	0.782	8/51 (15.69)	20/84 (23.81)	0.259
	Necrotising enterocolitis (any stage), *N (%)*	7/62 (11.29)	3/84 (3.57)	0.097	3/51 (5.88)	3/84 (3.57)	0.673
	Intraventricular haemorrhage grade III-IV[t], *N (%)*	11/62 (17.74)	8/84 (9.52)	0.145	1/51 (1.96)	8/84 (9.52)	0.153
	Electrical-only and/or electroclinical seizures, *N (%)*	2/62 (3.23)	0/84 (0.00)	0.179	2/51 (3.92)	0/84 (0.00)	0.141
	Early-onset and/or late-onset sepsis, *N (%)*	18/62 (29.03)*	4/84 (4.76)*	<0.001*	8/51 (15.69)	4/84 (4.76)	0.057
	Retinopathy of prematurity (any stage)[u], *N (%)*	15/56 (26.79)	12/84 (14.29)	0.066	15/51 (29.41)*	12/84 (14.29)*	0.033*
	Congenital hearing loss[v], *N (%)*	0/54 (0.00)	1/84 (1.19)	1.000	0/51 (0.00)	1/84 (1.19)	1.000
	Bronchopulmonary dysplasia[w], *N (%)*	5/53 (9.43)	3/84 (3.57)	0.260	3/51 (5.88)	3/84 (3.57)	0.673
	Jaundice requiring phototherapy and/or exchange transfusion[x], *N (%)*	45/62 (72.58)	55/84 (65.48)	0.361	41/51 (80.39)	55/84 (65.48)	0.064
	Anaemia requiring one or more red blood cell transfusions[y], *N (%)*	26/62 (41.93)*	19/84 (22.62)*	0.012*	16/51 (31.37)	19/84 (22.62)	0.260
Postnatal variables: metabolic disorders	Hyperglycaemia during HS[z], *N (%)*	14/62 (22.58)*	6/84 (7.14)*	0.007*	7/51 (13.72)	6/84 (7.14)	0.238
	Hypoglycaemia during HS[&], *N (%)*	12/62 (19.35)	9/84 (10.71)	0.141	8/51 (15.69)	9/84 (10.71)	0.399
	Hypophosphataemic hypercalcaemia during HS[/], *N (%)*	8/62 (12.90)	14/84 (16.67)	0.530	6/51 (11.76)	14/84 (16.67)	0.437
	Hypertriglyceridaemia during HS[(], *N (%)*	7/62 (11.29)	6/84 (7.14)	0.384	5/51 (9.80)	6/84 (7.14)	0.747
Postnatal variables: acid-base disorders	Metabolic acidosis during HS[)], *N (%)*	21/62 (33.87)*	9/84 (10.71)*	<0.001*	11/51 (21.57)	9/84 (10.71)	0.085
	Metabolic alkalosis during HS[=], *N (%)*	1/62 (1.61)	0/84 (0.00)	0.425	1/51 (1.96)	0/84 (0.00)	0.378
	Respiratory acidosis during HS[?], *N (%)*	8/62 (12.90)	9/84 (10.71)	0.684	5/51 (9.80)	9/84 (10.71)	0.866
	Respiratory alkalosis during HS^, *N (%)*	2/62 (3.23)	6/84 (7.14)	0.467	2/51 (3.92)	6/84 (7.14)	0.710
Postnatal variables: electrolyte disturbances	Hypernatraemia during HS[∞], *N (%)*	11/62 (17.74)	13/84 (15.48)	0.715	4/51 (7.84)	13/84 (15.48)	0.195
	Hyponatraemia during HS[§], *N (%)*	33/62 (53.23)	35/84 (41.67)	0.166	27/51 (52.94)	35/84 (41.67)	0.202
	Hyperkalaemia during HS [>], *N (%)*	18/62 (29.03)	15/84 (17.86)	0.111	12/51 (23.53)	15/84 (17.86)	0.424
	Hypokalaemia during HS[<], *N (%)*	4/62 (6.45)	5/84 (5.95)	1.000	2/51 (3.92)	5/84 (5.95)	0.710

	Hyperchloraemia during HS[•], *N (%)*	30/62 (48.39)	41/84 (48.81)	0.960	23/51 (45.10)	41/84 (48.81)	0.675
	Hypochloraemia during HS[□], *N (%)*	8/62 (12.90)	9/84 (10.71)	0.684	8/51 (15.69)	9/84 (10.71)	0.399
	Hypercalcaemia during HS[◊], *N (%)*	12/62 (19.35)	15/84 (17.86)	0.818	11/51 (21.57)	15/84 (17.86)	0.596
	Hypocalcaemia during HS[϶], *N (%)*	12/62 (19.35)	11/84 (13.09)	0.305	8/51 (15.69)	11/84 (13.09)	0.675
	Hyperphosphataemia during HS[⌈], *N (%)*	14/62 (22.58)	19/84 (22.62)	0.996	9/51 (17.65)	19/84 (22.62)	0.490
	Hypophosphataemia during HS[⌉], *N (%)*	12/62 (19.35)	19/84 (22.62)	0.634	10/51 (19.61)	19/84 (22.62)	0.680
	Hypermagnesaemia during HS[#], *N (%)*	26/62 (41.93)	30/84 (35.71)	0.445	17/51 (33.33)	30/84 (35.71)	0.778
	Hypomagnesaemia during HS[@], *N (%)*	4/62 (6.45)	9/84 (10.71)	0.371	4/51 (7.84)	9/84 (10.71)	0.766
Postnatal variables: nutritional data	Start of enteral nutrition, *days of life*	2.00 (4.00)	1.00 (1.75)	0.073	1.00 (2.00)	1.00 (1.75)	0.531
	Maternal milk intake DoL 0-7, *ml/kg/first week of life*	11.44 (80.51)	23.98 (220.22)	0.090	12.26 (106.07)	23.98 (220.22)	0.273
	Total milk intake DoL 0-7, *ml/kg/first week of life*	65.65 (253.12)*	223.91 (387.60)*	0.006*	85.23 (304.02)	223.91 (387.60)	0.060
	Maternal milk intake DoL 0-7/Total milk intake DoL 0-7, *%*	34.10 (66.73)	32.36 (70.64)	0.936	32.55 (61.78)	32.36 (70.64)	0.992
	Duration of parenteral nutrition, *days*	13.50 (15.00)*	9.00 (12.00)*	0.009*	14.00 (15.00)*	9.00 (12.00)*	0.041*
Postnatal variables: body growth	Postnatal weight loss, *%*	-13.06 ± 5.86	-11.39 ± 3.96	0.055	-12.26 ± 5.17	-11.39 ± 3.96	0.305
	Recovery of birth weight, *DoL*	12.00 (4.00)	12.00 (5.00)	0.951	12.00 (4.00)	12.00 (5.00)	0.879
	Body weight at DoL 14 Z-Score[k]	-1.07 (1.40)	-1.35 (1.46)	0.580	-1.00 (1.32)	-1.35 (1.46)	0.430
	Head circumference at DoL 14 Z-Score[k]	-0.06 (1.80)	-0.34 (1.43)	0.534	-0.06 (1.66)	-0.34 (1.43)	0.506
	Body weight at 36 weeks' PMA Z-Score[k]	-1.51 (1.79)	-1.78 (1.68)	0.396	-1.51 (1.62)	-1.78 (1.68)	0.397
	Head circumference at 36 weeks' PMA Z-Score[k]	-1.11 ± 1.31	-0.76 ± 1.21	0.107	-1.07 ± 1.32	-0.76 ± 1.21	0.155
	ΔZ-score body weight 36 weeks' PMA-DoL 14[k,σ]	-0.08 (0.74)	-0.20 (0.56)	0.310	-0.08 (0.72)	-0.20 (0.56)	0.281
	ΔZ-score head circumference 36 weeks' PMA-DoL 14[k,ψ]	-0.80 ± 1.03*	-0.38 ± 1.15*	0.036*	-0.75 ± 1.01	-0.38 ± 1.15	0.066
Postnatal variables: HD	PMA at hospital discharge, *weeks*	38.00 (4.00)	38.00 (3.00)	0.303	38.00 (4.00)	38.00 (3.00)	0.303

For legend and list of abbreviations, see Supplementary Table S1.

Supplementary Table S5. Edge lists of the domain-specific directed acyclic graphs used to review the candidate covariates before entry into the multivariable models.

**Edge (cause → effect)**	**Role in covariate selection**
Maternal educational level → developmental environment after discharge (unmeasured) → neurodevelopment	Confounder proxy; retained in the cognitive models
Male sex → neurodevelopment	Non-modifiable common cause; retained
Maternal smoking during pregnancy → fetal growth; → neurodevelopment	Confounder; retained where significant at univariate analysis
Chorioamnionitis → gestational age at birth	Confounder of the gestational-age effect; retained
Chorioamnionitis → fetal inflammatory response → neurodevelopment	Direct pathway of interest; retained
Chorioamnionitis → early-onset sepsis → duration of antibiotic prophylaxis/therapy	Duration of antibiotics is a descendant of sepsis and a marker of its severity; entered only as a severity index, never interpreted causally
Antenatal corticosteroids → respiratory distress syndrome → invasive mechanical ventilation	Antenatal corticosteroids retained as a prenatal confounder; respiratory distress not entered separately (near-duplicate of ventilation)
Gestational age → birth weight	Birth weight is a descendant of gestational age and of the prenatal growth variables already entered; not retained in any model
Intrauterine growth restriction, hypertensive disorders, abnormal Doppler flow → birth weight	Prenatal determinants of growth available as candidates; make birth weight redundant
Gestational age → invasive mechanical ventilation → bronchopulmonary dysplasia → postnatal corticosteroids → postmenstrual age at discharge	Chain of mediators; the upstream variable (gestational age) and the downstream summary (postmenstrual age at discharge) were retained, the intermediate steps were not entered together
Gestational age → intraventricular haemorrhage grade III–IV → neurodevelopment	Exposure of primary interest; retained with gestational age as confounder
Invasive mechanical ventilation, oxygen exposure → retinopathy of prematurity → neurodevelopment	Retinopathy retained as a marker of respiratory morbidity and oxygen exposure
Late-onset sepsis → duration of antibiotic prophylaxis/therapy	Near-duplicate measurement of the same construct; both entered only where collinearity diagnostics allowed
Late-onset sepsis, necrotising enterocolitis, haemodynamic instability → interruption of enteral feeding → duration of parenteral nutrition	Nutritional variables are mediators of the morbidities; coefficients attenuated towards the null when entered together
Total milk intake DoL 0–7 → maternal milk as a proportion of total intake	The proportion is by construction the ratio of two retained variables; not entered
Illness severity (unmeasured) → iatrogenic blood loss → anaemia requiring transfusion	Transfused anaemia entered as a severity index, not as an exposure
All neonatal morbidities → postmenstrual age at discharge	Postmenstrual age at discharge is a summary of the whole stay, interpreted as a marker of illness severity and not as a modifiable exposure
Death between DoL 8 and 24 months ← the same morbidities that affect neurodevelopment	Competing event; motivates the composite primary outcome and the sensitivity analysis of Supplementary Table S11

Legend: The table reports, in the form of an edge list, the causal structure that was assumed when the candidate covariates significant at univariate analysis were reviewed on clinical and causal grounds. The core structure is common to the four domains; the domain-specific graphs differ only in the exposures that reached the univariate threshold for that domain. Variables lying on the pathway between a retained covariate and the outcome, and variables constituting near-duplicate measurements of the same construct, were not entered; confounders were retained. The graphs were used qualitatively, to remove redundancy and to avoid adjusting for mediators, and not to derive a formal minimal sufficient adjustment set, which the number of events would not have supported.

Abbreviation: DoL, day of life.

Supplementary Table S6. Collinearity diagnostics of the eight multivariable models beyond the variance inflation factor.

Model	Covariates	Largest VIF	Smallest tolerance	Largest absolute pairwise correlation (pair)	Largest condition index
S12 – Cognitive, composite outcome	20	3.20	0.313	0.663 (duration of antibiotics / sepsis)	7.0
S13 – Language, composite outcome	14	2.91	0.344	0.687 (maternal milk / total milk intake DoL 0–7)	5.1
S14 – Motor, composite outcome	20	3.40	0.294	0.663 (duration of antibiotics / sepsis)	7.3
S15 – Socio-emotional, composite outcome	13	2.77	0.361	0.687 (duration of antibiotics / sepsis)	4.6
S16 – Cognitive, survivors only	15	3.05	0.328	0.663 (duration of antibiotics / sepsis)	5.3
S17 – Language, survivors only	11	2.73	0.366	0.620 (total milk intake DoL 0–7 / parenteral nutrition)	4.0
S18 – Motor, survivors only	16	3.88	0.257	0.663 (duration of antibiotics / sepsis)	6.1
S19 – Socio-emotional, survivors only	3	1.07	0.936	0.244 (retinopathy of prematurity / parenteral nutrition)	1.3

Legend: Tolerance is the complement of the coefficient of determination obtained by regressing each covariate on all the others and equals the reciprocal of the variance inflation factor; values below 0.10 are conventionally regarded as indicating problematic collinearity. Condition indices were computed as the ratio of the largest singular value to each singular value of the column-centred and column-scaled design matrix; values above 30 are conventionally regarded as indicating problematic collinearity. Diagnostics were computed on the participants with complete data for all covariates entered in the corresponding model (n = 133–135). Gestational age and birth weight were correlated in the cohort (r = 0.620) but were never simultaneously adjusted for: birth weight was not retained in any of the models. Diagnostics are not reported separately for the four primary composite models of Figures 2–5, whose covariates are a subset of those of the corresponding models S12–S15, so that the removal of covariates can only reduce the collinearity reported here. Two caveats apply to these diagnostics. Tolerance is by construction the reciprocal of the variance inflation factor and therefore adds no information beyond it; the diagnostics that are genuinely additional to the variance inflation factor are the largest absolute pairwise correlation and the condition index. Condition indices were computed on the column-centred and column-scaled design matrix, whereas the conventional threshold of 30 was derived for the scaled but uncentred matrix; centring lowers the indices systematically, so that the values reported here should be read as a relative comparison between models rather than against that threshold.

List of abbreviations: DoL, day of life; VIF, variance inflation factor.

Supplementary Table S7. Events per variable and internal validation of the twelve multivariable binary logistic regression models.

Model (outcome)	n	Cases / controls	Covariates	EPV	Apparent c	Optimism	Optimism-corrected c	Calibration slope	Nagelkerke R^2
Primary – Cognitive, composite outcome (Figure 2)	146	64 / 82	17	3.8	0.798	0.095	0.703	0.55	0.367
Primary – Language, composite outcome (Figure 3)	146	78 / 68	12	5.7	0.768	0.072	0.696	0.60	0.284
Primary – Motor, composite outcome (Figure 4)	146	45 / 101	16	2.8	0.825	0.086	0.739	0.57	0.507
Primary – Socio-emotional, composite outcome (Figure 5)	146	62 / 84	12	5.2	0.753	0.081	0.672	0.56	0.267
S12 – Cognitive, composite outcome (sensitivity, complete covariate set)	133	53 / 80	20	2.6	0.797	0.126	0.671	0.41	0.333
S13 – Language, composite outcome (sensitivity, complete covariate set)	135	67 / 68	14	4.8	0.741	0.098	0.643	0.43	0.238
S14 – Motor, composite outcome (sensitivity, complete covariate set)	133	34 / 99	20	1.7	0.832	0.140	0.692	0.37	0.439
S15 – Socio-emotional, composite outcome (sensitivity, complete covariate set)	135	53 / 82	13	4.1	0.711	0.112	0.598	0.43	0.172
S16 – Cognitive, survivors only	133	53 / 80	15	3.5	0.764	0.100	0.664	0.48	0.289
S17 – Language, survivors only	135	67 / 68	11	6.1	0.736	0.073	0.663	0.57	0.255
S18 – Motor, survivors only	133	34 / 99	16	2.1	0.820	0.116	0.704	0.49	0.416
S19 – Socio-emotional, survivors only	135	51 / 84	3	17.0	0.677	0.024	0.653	0.88	0.116

Legend: Cases are infants with a pathological Bayley-III score on the domain considered or, for the composite outcome, death between DoL 8 and the day of neurodevelopmental assessment at 24 months' CA; controls are infants with a normal score on the scale considered. EPV, events per variable, calculated as the number of participants in the less frequent of the two outcome categories divided by the number of estimated covariate parameters; the conventional threshold is 10. Apparent c, c-statistic (area under the receiver operating characteristic curve) in the sample in which the model was fitted. Optimism and the optimism-corrected c-statistic were obtained by non-parametric bootstrapping with 1000 resamples and the covariate set held fixed. Calibration slope, mean slope of the logistic regression of the observed outcome on the linear predictor derived from each bootstrap model; a value below 1 indicates that the regression coefficients require shrinkage by that factor. Performance measures were computed on the participants with complete data for all covariates entered in the corresponding model (n = 133–135). Resamples in which the maximum-likelihood estimate failed to converge because of near-complete separation were excluded from the primary estimate (0.1% of resamples in model S19, 1.4% in S15, 5.3% in S12, 8.2% in S18, 10.0% in S14, 25.5% in S17, 25.7% in S16 and 27.5% in S13); a sensitivity analysis including them changed the optimism-corrected c-statistic by no more than 0.004 and yielded calibration slopes between 0.33 and 0.46. Because the covariate set was held fixed within the bootstrap, the procedure does not account for the optimism introduced by the preceding univariate selection step, so that the optimism reported here is a lower bound and the optimism-corrected c-statistic and the calibration slope are correspondingly upper bounds of the internal validity of the models, that is, still optimistic. The columns n and Cases / controls refer to the analytic sample of each model, that is to the participants with complete data for all of the covariates entered, and not to the whole cohort of 146 infants; events per variable are computed on that sample. The four models labelled Primary are those displayed in Figures 2–5 and reported in full in Supplementary Table S11: they are restricted to the covariates that are defined in every infant of the cohort, so that all 146 infants, including the 11 who died,

contribute to the estimation, and their optimism and calibration slope were obtained by the same bootstrap procedure with 1000 resamples and the covariate set held fixed. Models S12–S15 are the corresponding sensitivity analysis, retaining the complete covariate set: because no infant who died had been discharged, and because the postmenstrual age at hospital discharge, the retinopathy status and the growth Z-scores at 36 weeks' postmenstrual age are undefined before the corresponding time point, listwise deletion left 0 of the 11 deaths in models S12, S13 and S14 and 2 of the 11 in model S15, so that models S12 and S14 have the same outcome distribution as models S16 and S18 respectively.

List of abbreviations: c, concordance statistic; CA, corrected age; DoL, day of life; EPV, events per variable.

Supplementary Table S8. Control of the false discovery rate among the univariate comparisons reported in Supplementary Tables S1–S4, of which Tables 1–4 are the abridged versions.

Outcome	Comparison	Tests performed	p < 0.05	q < 0.05	q < 0.10
Cognitive	Composite outcome	81	24	19	21
Language	Composite outcome	81	15	9	9
Motor	Composite outcome	81	24	22	23
Socio-emotional	Composite outcome	81	14	2	6
Cognitive	Survivors only	81	15	0	1
Language	Survivors only	81	11	0	0
Motor	Survivors only	81	19	15	17
Socio-emotional	Survivors only	81	3	0	0

Legend: The Benjamini-Hochberg procedure was applied separately within each of the eight families of univariate comparisons, a family being defined as all variables tested against one outcome in one of the two comparisons (648 tests in total). q is the Benjamini-Hochberg adjusted p value, that is the estimated false discovery rate at which the association would be declared significant. Values reported as "< 0.001" in Supplementary Tables S1–S4 were treated as 0.001. The number of tests per family (81) is the number of candidate explanatory variables for which a p value is reported in Supplementary Tables S1–S4; the row reporting the Bayley-III score itself, being the outcome rather than a candidate explanatory variable, is excluded.

Supplementary Table S9. Control of the false discovery rate among the covariates of the twelve multivariable models.

Model	Covariate	p	q within the model	q across the 169 covariate tests
Primary – Language, composite outcome	Intraventricular haemorrhage grade III–IV	0.013	0.156	0.507
Primary – Motor, composite outcome	Early-onset and/or late-onset sepsis	0.010	0.160	0.507
Primary – Motor, composite outcome	Anaemia requiring one or more RBC transfusions	0.028	0.224	0.507
Primary – Socio-emotional, composite outcome	Male sex	0.027	0.162	0.507
Primary – Socio-emotional, composite outcome	Early-onset and/or late-onset sepsis	0.027	0.162	0.507
Primary – Socio-emotional, composite outcome	Maternal smoking during pregnancy	0.046	0.184	0.507
S12 – Cognitive, composite outcome	Maternal high school diploma or higher	0.045	0.352	0.507
S12 – Cognitive, composite outcome	Retinopathy of prematurity (any stage)	0.046	0.352	0.507
S13 – Language, composite outcome	Intraventricular haemorrhage grade III–IV	0.025	0.345	0.507
S14 – Motor, composite outcome	PMA at hospital discharge	0.017	0.337	0.507
S15 – Socio-emotional, composite outcome	Male sex	0.046	0.531	0.507
S16 – Cognitive, survivors only	Chorioamnionitis	0.032	0.283	0.507
S17 – Language, survivors only	Intraventricular haemorrhage grade III–IV	0.047	0.303	0.507
S18 – Motor, survivors only	Early-onset and/or late-onset sepsis	0.024	0.382	0.507
S19 – Socio-emotional, survivors only	Male sex	0.019	0.057	0.507

Legend: The table lists every covariate that reached the nominal significance threshold ($p < 0.05$) in the twelve multivariable models fitted — the four primary composite models of Figures 2–5 and Supplementary Table S11, the four sensitivity composite models of Supplementary Tables S12–S15 and the four survivors-only models of Supplementary Tables S16–S19. The Benjamini-Hochberg procedure was applied twice: within each model, the family being all covariates of that model, and across all 169 covariate tests performed in the twelve models. No covariate remained significant at a false discovery rate below 5% under either definition of the family; the smallest adjusted value within a model was $q = 0.156$, and across the 169 tests the adjusted value was $q = 0.507$ for every nominally significant covariate. No covariate reached the nominal threshold in the primary model of the cognitive domain. q is the Benjamini-Hochberg adjusted p value.

List of abbreviations: PMA, postmenstrual age.

Supplementary Table S10. Analysis variables grouped by category.

Category	Subgroup	Variable	Type	Unit
**Prenatal**	Maternal characteristics	Maternal age	Continuous	years
	Maternal characteristics	Maternal ethnicity: Caucasian	Categorical	–
	Maternal characteristics	Maternal high school diploma or higher educational level	Categorical	–
	Maternal characteristics	Maternal university degree or higher educational level	Categorical	–
	Pregnancy	Maternal smoking during pregnancy	Categorical	–
	Pregnancy	Maternal alcohol consumption during pregnancy	Categorical	–
	Pregnancy	Maternal drug use during pregnancy	Categorical	–
	Pregnancy	Maternal thyroid disorders during pregnancy	Categorical	–
	Pregnancy	Maternal diabetes during pregnancy	Categorical	–
	Pregnancy	Intrauterine growth restriction	Categorical	–
	Pregnancy	Maternal pressure disorders during pregnancy	Categorical	–
	Pregnancy	Alterations of Doppler flow velocimetry	Categorical	–
**Perinatal**	Delivery and first hour of life	Placental abruption	Categorical	–
	Delivery and first hour of life	Chorioamnionitis	Categorical	–
	Delivery and first hour of life	One or more risk factors for intrapartum infection	Categorical	–
	Delivery and first hour of life	Intrapartum antibiotics $\geq$ 4 hours before birth	Categorical	–
	Delivery and first hour of life	Antenatal corticosteroids	Continuous	number of doses
	Delivery and first hour of life	Caesarean section	Categorical	–
	Delivery and first hour of life	Male sex	Categorical	–
	Delivery and first hour of life	Gestational age	Continuous	weeks

Category	Subgroup	Variable	Type	Unit
**Perinatal**	Delivery and first hour of life	Birth weight	Continuous	g
	Delivery and first hour of life	Birth weight Z-score	Continuous	
	Delivery and first hour of life	Head circumference	Continuous	cm
	Delivery and first hour of life	Head circumference Z-score	Continuous	
	Delivery and first hour of life	Twin infants	Categorical	–
	Delivery and first hour of life	5-minute Apgar score	Continuous	
	Delivery and first hour of life	Umbilical cord arterial pH	Continuous	
	Delivery and first hour of life	Umbilical cord arterial base deficit	Continuous	
	Delivery and first hour of life	Body temperature at 1 hour of life	Continuous	°C
**Postnatal (clinical)**	Respiratory support	Invasive mechanical ventilation	Categorical	–
	Respiratory support	Surfactant	Continuous	number of doses
	Medications	Duration of fentanyl administration	Continuous	days
	Medications	Duration of caffeine administration	Continuous	days
	Medications	Duration of postnatal corticosteroid therapy	Continuous	days
	Medications	Duration of diuretic therapy	Continuous	days
	Medications	Duration of antibiotic prophylaxis/therapy	Continuous	days
	Medications	Duration of inotropic support	Continuous	days
	Morbidities	Haemodynamically significant patent ductus arteriosus	Categorical	–
	Morbidities	Necrotising enterocolitis (any stage)	Categorical	–
	Morbidities	Intraventricular haemorrhage grade III–IV	Categorical	–
	Morbidities	Electrical-only and/or electroclinical seizures	Categorical	–

Category	Subgroup	Variable	Type	Unit
**Postnatal (clinical)**	Morbidities	Early-onset and/or late-onset sepsis	Categorical	–
	Morbidities	Retinopathy of prematurity (any stage)	Categorical	–
	Morbidities	Congenital hearing loss	Categorical	–
	Morbidities	Bronchopulmonary dysplasia	Categorical	–
	Morbidities	Jaundice requiring phototherapy and/or exchange transfusion	Categorical	–
	Morbidities	Anaemia requiring one or more red blood cell transfusions	Categorical	–
	Hospital discharge	PMA at hospital discharge	Continuous	weeks
**Nutritional**	Nutrition	Start of enteral nutrition	Continuous	days of life
	Nutrition	Maternal milk intake DoL 0-7	Continuous	ml/kg/first week of life
	Nutrition	Total milk intake DoL 0-7	Continuous	ml/kg/first week of life
	Nutrition	Maternal milk intake DoL 0-7/Total milk intake DoL 0-7	Continuous	%
	Nutrition	Duration of parenteral nutrition	Continuous	days
**Growth**	Postnatal growth	Postnatal weight loss	Continuous	%
	Postnatal growth	Recovery of birth weight	Continuous	DoL
	Postnatal growth	Body weight at DoL 14 Z-score	Continuous	
	Postnatal growth	Head circumference at DoL 14 Z-score	Continuous	
	Postnatal growth	Body weight at 36 weeks' PMA Z-score	Continuous	
	Postnatal growth	Head circumference at 36 weeks' PMA Z-score	Continuous	
	Postnatal growth	ΔZ-score body weight, 36 weeks' PMA − DoL 14	Continuous	
	Postnatal growth	ΔZ-score head circumference, 36 weeks' PMA − DoL 14	Continuous	
**Metabolic**	Metabolic disorders	Hyperglycaemia during hospital stay	Categorical	–

Category	Subgroup	Variable	Type	Unit
**Metabolic**	Metabolic disorders	Hypoglycaemia during hospital stay	Categorical	–
	Metabolic disorders	Hypophosphataemic hypercalcaemia during hospital stay	Categorical	–
	Metabolic disorders	Hypertriglyceridaemia during hospital stay	Categorical	–
**Acid-base**	Acid-base disorders	Metabolic acidosis during hospital stay	Categorical	–
	Acid-base disorders	Metabolic alkalosis during hospital stay	Categorical	–
	Acid-base disorders	Respiratory acidosis during hospital stay	Categorical	–
	Acid-base disorders	Respiratory alkalosis during hospital stay	Categorical	–
**Electrolyte**	Electrolyte disturbances	Hypernatraemia during hospital stay	Categorical	–
	Electrolyte disturbances	Hyponatraemia during hospital stay	Categorical	–
	Electrolyte disturbances	Hyperkalaemia during hospital stay	Categorical	–
	Electrolyte disturbances	Hypokalaemia during hospital stay	Categorical	–
	Electrolyte disturbances	Hyperchloraemia during hospital stay	Categorical	–
	Electrolyte disturbances	Hypochloraemia during hospital stay	Categorical	–
	Electrolyte disturbances	Hypercalcaemia during hospital stay	Categorical	–
	Electrolyte disturbances	Hypocalcaemia during hospital stay	Categorical	–
	Electrolyte disturbances	Hyperphosphataemia during hospital stay	Categorical	–
	Electrolyte disturbances	Hypophosphataemia during hospital stay	Categorical	–
	Electrolyte disturbances	Hypermagnesaemia during hospital stay	Categorical	–
	Electrolyte disturbances	Hypomagnesaemia during hospital stay	Categorical	–

Legend: The table lists the 81 candidate explanatory variables analysed in this study, grouped into the eight categories used in the Category column — prenatal (12 variables), perinatal (17), postnatal clinical (19), nutritional (5), growth (8), metabolic (4), acid-base (4) and electrolyte (12) — which resolve into the five broader groupings requested during peer review, the growth variables belonging with the nutritional ones and the acid-base and electrolyte variables with the metabolic ones. Prenatal variables were ascertained from the maternal and obstetric records, perinatal variables refer to delivery and to the first hour of life, postnatal clinical variables to the whole hospital stay up to discharge, nutritional variables to enteral and parenteral intakes, growth variables to postnatal weight loss and its recovery and to the body weight and head circumference Z-scores at day 14 and at 36 weeks' postmenstrual age and their change between those time points, and the metabolic, acid-base and electrolyte variables to the corresponding disturbances recorded during the stay. The four Bayley-III outcome scores and the

derived outcome variables are not listed here. Definitions of the variables that may be open to interpretation are given in the legend to Supplementary Table S1. Of these 81 variables, 66 were complete and 15 had at least one missing value; adding the four domain-specific outcome variables of the survivors-only analysis, which are undefined in the 11 infants who died, 19 variables in all had at least one missing value (Supplementary Table S21).

Supplementary Table S11. Primary analysis of the composite outcome: complete results of the four models displayed in Figures 2–5, restricted to the covariates that are defined in every infant so that all 146 infants, including the 11 who died, contributed to the estimation.

Covariate	B	S.E.	Wald	p	Exp(B)	95% C.I.	Firth Exp(B)	Firth p
Panel (a) Cognitive domain, composite outcome - n = 146 (11 deaths included); cases / controls 64 / 82; 17 covariates; EPV 3.8; Nagelkerke R2 = 0.367; apparent c = 0.798; optimism-corrected c = 0.703; bootstrap calibration slope = 0.55								
Maternal age, years	0.033	0.039	0.721	0.396	1.034	0.958 – 1.115	1.028	0.456
Maternal high school diploma or higher educational level	0.862	0.498	2.989	0.084	2.367	0.891 – 6.285	2.071	0.127
Antenatal corticosteroids, number of doses	0.454	0.271	2.818	0.093	1.575	0.927 – 2.677	1.468	0.140
Gestational age, weeks	-0.023	0.119	0.039	0.844	0.977	0.773 – 1.234	0.982	0.877
5-minute Apgar score	0.369	0.207	3.188	0.074	1.447	0.965 – 2.169	1.369	0.113
Invasive mechanical ventilation	0.732	0.571	1.646	0.199	2.080	0.680 – 6.364	1.762	0.303
Duration of postnatal corticosteroid therapy, days	-0.056	0.074	0.581	0.446	0.945	0.818 – 1.093	0.949	0.450
Duration of antibiotic prophylaxis/therapy, days	0.077	0.053	2.092	0.148	1.080	0.973 – 1.198	1.063	0.194
Duration of inotropic support, days	-0.070	0.208	0.112	0.738	0.933	0.620 – 1.404	1.023	0.887
Early-onset and/or late-onset sepsis	-2.133	1.122	3.616	0.057	0.118	0.013 – 1.068	0.181	0.092
Anaemia requiring one or more RBC transfusions	-0.196	0.575	0.116	0.733	0.822	0.266 – 2.537	0.818	0.715
Hyperglycaemia during hospital stay	-1.238	0.787	2.478	0.115	0.290	0.062 – 1.355	0.356	0.157
Metabolic acidosis during hospital stay	-0.258	0.646	0.160	0.690	0.772	0.218 – 2.741	0.802	0.725
Maternal milk intake DoL 0-7, ml/kg/first week	0.002	0.002	1.299	0.254	1.002	0.998 – 1.007	1.002	0.291
Total milk intake DoL 0-7, ml/kg/first week	0.000	0.002	0.104	0.747	1.000	0.998 – 1.003	1.000	0.763
Duration of parenteral nutrition, days	-0.015	0.025	0.357	0.550	0.985	0.937 – 1.035	0.990	0.642
Postnatal weight loss, %	0.051	0.051	0.998	0.318	1.053	0.952 – 1.164	1.044	0.374
Panel (b) Language domain, composite outcome - n = 146 (11 deaths included); cases / controls 78 / 68; 12 covariates; EPV 5.7; Nagelkerke R2 = 0.284; apparent c = 0.768; optimism-corrected c = 0.696; bootstrap calibration slope = 0.60								
Gestational age, weeks	0.054	0.101	0.284	0.594	1.055	0.866 – 1.286	1.051	0.614
Invasive mechanical ventilation	0.652	0.554	1.387	0.239	1.920	0.648 – 5.684	1.776	0.285
Duration of antibiotic prophylaxis/therapy, days	-0.033	0.042	0.626	0.429	0.968	0.892 – 1.050	0.973	0.487
Duration of inotropic support, days	0.098	0.112	0.764	0.382	1.103	0.885 – 1.375	1.086	0.434
Intraventricular haemorrhage grade III-IV	-2.782	1.126	6.107	0.013*	0.062	0.007 – 0.562	0.107	0.016
Early-onset and/or late-onset sepsis	-0.729	1.057	0.475	0.491	0.483	0.061 – 3.831	0.601	0.603
Anaemia requiring one or more RBC transfusions	0.023	0.556	0.002	0.967	1.023	0.344 – 3.042	1.003	0.995
Hyperglycaemia during hospital stay	-0.973	0.755	1.664	0.197	0.378	0.086 – 1.658	0.453	0.264
Metabolic acidosis during hospital stay	-0.504	0.653	0.596	0.440	0.604	0.168 – 2.172	0.657	0.504
Maternal milk intake DoL 0-7, ml/kg/first week	0.001	0.002	0.604	0.437	1.001	0.998 – 1.005	1.001	0.450
Total milk intake DoL 0-7, ml/kg/first week	0.001	0.001	0.521	0.471	1.001	0.998 – 1.004	1.001	0.494

Duration of parenteral nutrition, days	-0.004	0.022	0.041	0.840	0.996	0.953 – 1.040	0.997	0.866
Panel (c) Motor domain, composite outcome - n = 146 (11 deaths included); cases / controls 45 / 101; 16 covariates; EPV 2.8; Nagelkerke R2 = 0.507; apparent c = 0.825; optimism-corrected c = 0.739; bootstrap calibration slope = 0.57								
Antenatal corticosteroids, number of doses	0.085	0.329	0.066	0.797	1.088	0.571 – 2.074	1.059	0.849
Gestational age, weeks	-0.005	0.133	0.002	0.968	0.995	0.766 – 1.292	1.002	0.984
Invasive mechanical ventilation	-0.267	0.652	0.167	0.683	0.766	0.213 – 2.751	0.687	0.530
Duration of fentanyl administration, days	-0.009	0.074	0.015	0.902	0.991	0.857 – 1.146	1.016	0.714
Duration of postnatal corticosteroid therapy, days	0.054	0.082	0.431	0.512	1.055	0.899 – 1.238	1.031	0.681
Duration of antibiotic prophylaxis/therapy, days	0.125	0.068	3.355	0.067	1.133	0.991 – 1.295	1.087	0.120
Duration of inotropic support, days	-0.247	0.246	1.004	0.316	0.781	0.482 – 1.266	0.897	0.556
Intraventricular haemorrhage grade III-IV	0.030	0.948	0.001	0.975	1.031	0.161 – 6.608	0.987	0.988
Early-onset and/or late-onset sepsis	-3.999	1.549	6.664	0.010*	0.018	0.001 – 0.382	0.056	0.019
Anaemia requiring one or more RBC transfusions	-1.444	0.657	4.829	0.028*	0.236	0.065 – 0.856	0.288	0.039
Hyperglycaemia during hospital stay	-0.422	0.809	0.272	0.602	0.656	0.134 – 3.202	0.670	0.587
Metabolic acidosis during hospital stay	0.067	0.721	0.009	0.926	1.070	0.260 – 4.394	1.025	0.971
Maternal milk intake DoL 0-7, ml/kg/first week	-0.001	0.003	0.044	0.833	0.999	0.994 – 1.004	1.000	0.848
Total milk intake DoL 0-7, ml/kg/first week	0.001	0.002	0.399	0.528	1.001	0.997 – 1.005	1.001	0.577
Duration of parenteral nutrition, days	-0.022	0.033	0.429	0.513	0.978	0.916 – 1.045	0.988	0.665
Postnatal weight loss, %	0.084	0.061	1.898	0.168	1.088	0.965 – 1.226	1.070	0.225
Panel (d) Socio-emotional domain, composite outcome - n = 146 (11 deaths included); cases / controls 62 / 84; 12 covariates; EPV 5.2; Nagelkerke R2 = 0.267; apparent c = 0.753; optimism-corrected c = 0.672; bootstrap calibration slope = 0.56								
Maternal smoking during pregnancy	-0.963	0.482	3.986	0.046*	0.382	0.148 – 0.983	0.414	0.061
Male sex	-0.882	0.400	4.877	0.027*	0.414	0.189 – 0.906	0.450	0.040
Gestational age, weeks	0.071	0.105	0.459	0.498	1.074	0.873 – 1.321	1.065	0.542
Invasive mechanical ventilation	0.252	0.531	0.225	0.635	1.287	0.454 – 3.644	1.230	0.691
Duration of antibiotic prophylaxis/therapy, days	0.062	0.042	2.174	0.140	1.064	0.980 – 1.155	1.054	0.184
Duration of inotropic support, days	0.029	0.110	0.070	0.792	1.030	0.829 – 1.279	1.032	0.760
Early-onset and/or late-onset sepsis	-2.117	0.956	4.908	0.027*	0.120	0.019 – 0.783	0.169	0.048
Anaemia requiring one or more RBC transfusions	0.128	0.546	0.055	0.815	1.136	0.389 – 3.316	1.081	0.884
Hyperglycaemia during hospital stay	-0.530	0.665	0.635	0.425	0.589	0.160 – 2.166	0.640	0.488
Metabolic acidosis during hospital stay	-0.643	0.611	1.108	0.292	0.526	0.159 – 1.741	0.573	0.352
Total milk intake DoL 0-7, ml/kg/first week	0.001	0.001	0.216	0.642	1.001	0.998 – 1.003	1.001	0.629
Duration of parenteral nutrition, days	-0.024	0.021	1.249	0.264	0.976	0.936 – 1.018	0.981	0.331

Legend: These are the primary models of the composite outcome, displayed as forest plots in Figures 2–5. Because the postmenstrual age at hospital discharge is undefined in every infant who died before discharge, and the retinopathy status and the growth Z-scores at 36 weeks' postmenstrual age are undefined in infants who died before those time points, entering any of these covariates would remove the deaths from the model by listwise deletion; they were therefore not entered, so that the composite outcome is estimated on the whole cohort of 146 infants as intended. The corresponding models retaining the complete covariate set, which achieve a more complete adjustment but which listwise deletion restricts to survivors, are reported as a sensitivity analysis in Supplementary Tables S12–S15. The dependent variable was coded so that the modelled event was normal neurodevelopment; Exp(B) > 1 indicates higher odds of a normal outcome and Exp(B) < 1 higher odds of the adverse or composite outcome. * $p < 0.05$ at the nominal level; no covariate remained significant after Benjamini-

Hochberg adjustment within its own model, the smallest adjusted value being q = 0.16. Firth Exp(B) and Firth p are the corresponding estimates obtained by Firth's penalised likelihood, used because of the low number of events per variable and of the near-complete separation observed for some covariates. Optimism, optimism-corrected c-statistic and calibration slope were obtained by non-parametric bootstrapping with 1000 resamples and the covariate set held fixed, as in Supplementary Table S7.

Abbreviations: DoL, day of life; EPV, events per variable; RBC, red blood cell; S.E., standard error.

Supplementary Table S12. Sensitivity analysis. Multivariate analysis, retaining the complete covariate set, of covariates influencing the composite outcome of pathological cognitive score at Bayley-III assessment and death between DoL 8 and 24 months' CA vs normal cognitive composite score at 24 months' CA in patients with GA at birth ≤ 32 weeks and/or BW ≤ 1500 g. [Nagelkerke R-squared = 0.333; Hosmer-Lemeshow test *p*-value = 0.168; Variance inflation factor (VIF) < 5 for all covariates]. Because the postmenstrual age at hospital discharge, the retinopathy status and the growth Z-scores at 36 weeks' PMA are undefined in infants who died before the corresponding time point, listwise deletion restricts this model to survivors; the primary analysis of this composite outcome, restricted to the covariates defined in every infant so that all 146 infants contribute, is reported in Figure 2 and Supplementary Table S11.

Dependent variable	Cognitive development at 24 months' CA and Death DoL 8-24 months' CA	B	S.E.	Wald	*p*-value	Exp(B)	95% C.I. for Exp(B)	
							Lower	Upper
Covariates	Maternal age, *years*	0.033	0.041	0.659	0.417	1.034	0.954	1.120
	Maternal high school diploma or higher educational level	1.092	0.545	4.008	0.045*	2.980	1.023	8.680
	Antenatal corticosteroids[j], *number of doses*	0.408	0.288	2.007	0.157	1.504	0.855	2.645
	Gestational age, *weeks*	-0.061	0.147	0.173	0.678	0.941	0.705	1.255
	5-minute Apgar score	0.363	0.218	2.765	0.096	1.437	0.937	2.204
	Invasive mechanical ventilation[l]	1.050	0.637	2.715	0.099	2.857	0.820	9.959
	Duration of postnatal corticosteroid therapy[p], *days*	-0.021	0.086	0.060	0.807	0.979	0.827	1.160
	Duration of antibiotic prophylaxis/therapy, *days*	0.052	0.055	0.898	0.343	1.053	0.946	1.172
	Duration of inotropic support[r], *days*	-0.146	0.263	0.309	0.578	0.864	0.516	1.447
	Early-onset and/or late-onset sepsis	-1.682	1.291	1.698	0.193	0.186	0.015	2.336
	Retinopathy of prematurity (any stage)[u]	-1.393	0.698	3.980	0.046*	0.248	0.063	0.976
	Anaemia requiring one or more red blood cell transfusions[y]	0.353	0.689	0.262	0.609	1.423	0.368	5.497
	Hyperglycaemia during HS[z]	-0.649	0.811	0.641	0.423	0.523	0.107	2.560
	Metabolic acidosis during HS[)]	0.311	0.750	0.172	0.678	1.365	0.314	5.931
	Maternal milk intake DoL 0-7, *ml/kg/first week of life*	0.003	0.002	1.291	0.256	1.003	0.998	1.007
	Total milk intake DoL 0-7, *ml/kg/first week of life*	0.000	0.002	0.024	0.877	1.000	0.996	1.003

	Duration of parenteral nutrition, *days*	-0.012	0.032	0.127	0.721	0.989	0.928	1.053
	Postnatal weight loss, *%*	0.029	0.056	0.264	0.608	1.029	0.922	1.148
	ΔZ-score head circumference 36 weeks' PMA-DoL 14[k,ψ]	0.433	0.224	3.748	0.053	1.542	0.995	2.392
	PMA at hospital discharge, *weeks*	-0.143	0.124	1.330	0.249	0.867	0.680	1.105

For Legend, see Supplementary Table S1.

List of abbreviations: BW, birth weight; CA, corrected age; C.I., confidence interval; DoL, day of life; GA, gestational age; HS, hospital stay; PMA, postmenstrual age; S.E., standard error; Δ, variation.

Supplementary Table S13. Sensitivity analysis. Multivariate analysis, retaining the complete covariate set, of covariates influencing the composite outcome of pathological language score at Bayley-III assessment and death between DoL 8 and 24 months' CA vs normal language composite score at 24 months' CA in patients with GA at birth ≤ 32 weeks and/or BW ≤ 1500 g. [Nagelkerke R-squared = 0.238; Hosmer-Lemeshow test *p*-value = 0.190; Variance inflation factor (VIF) < 5 for all covariates]. Because the postmenstrual age at hospital discharge, the retinopathy status and the growth Z-scores at 36 weeks' PMA are undefined in infants who died before the corresponding time point, listwise deletion restricts this model to survivors; the primary analysis of this composite outcome, restricted to the covariates defined in every infant so that all 146 infants contribute, is reported in Figure 3 and Supplementary Table S11.

Dependent variable	Language development at 24 months' CA and Death DoL 8-24 months' CA	B	S.E.	Wald	*p*-value	Exp(B)	95% C.I. for Exp(B)	
							Lower	Upper
Covariates	Gestational age, *weeks*	0.110	0.114	0.925	0.336	1.116	0.892	1.397
	Invasive mechanical ventilation[l]	0.958	0.590	2.640	0.104	2.606	0.821	8.277
	Duration of antibiotic prophylaxis/therapy, *days*	-0.033	0.043	0.584	0.445	0.968	0.890	1.053
	Duration of inotropic support[r], *days*	0.104	0.123	0.714	0.398	1.109	0.872	1.411
	Intraventricular haemorrhage grade III-IV[t]	-2.675	1.190	5.050	0.025*	0.069	0.007	0.710
	Early-onset and/or late-onset sepsis	-0.432	1.149	0.142	0.707	0.649	0.068	6.174
	Retinopathy of prematurity (any stage)[u]	-0.435	0.625	0.483	0.487	0.647	0.190	2.205
	Anaemia requiring one or more red blood cell transfusions[y]	0.511	0.629	0.658	0.417	1.666	0.485	5.721
	Hyperglycaemia during HS[z]	-0.736	0.782	0.886	0.347	0.479	0.103	2.219
	Metabolic acidosis during HS[)]	-0.269	0.701	0.147	0.701	0.764	0.193	3.020
	Maternal milk intake DoL 0-7, *ml/kg/first week of life*	0.002	0.002	1.084	0.298	1.002	0.998	1.005
	Total milk intake DoL 0-7, *ml/kg/first week of life*	0.001	0.001	0.345	0.557	1.001	0.998	1.003
	Duration of parenteral nutrition, *days*	0.007	0.027	0.076	0.782	1.007	0.956	1.062
	PMA at hospital discharge, *weeks*	-0.195	0.104	3.493	0.062	0.823	0.670	1.010

For Legend, see Supplementary Table S1.

List of abbreviations: BW, birth weight; CA, corrected age; C.I., confidence interval; DoL, day of life; GA, gestational age; HS, hospital stay; PMA, postmenstrual age; S.E., standard error.

Supplementary Table S14. Sensitivity analysis. Multivariate analysis, retaining the complete covariate set, of covariates influencing the composite outcome of pathological motor score at Bayley-III assessment and death between DoL 8 and 24 months' CA vs normal motor composite score at 24 months' CA in patients with GA at birth ≤ 32 weeks and/or BW ≤ 1500 g. [Nagelkerke R-squared = 0.439; Hosmer-Lemeshow test *p*-value = 0.906; Variance inflation factor (VIF) < 5 for all covariates]. Because the postmenstrual age at hospital discharge, the retinopathy status and the growth Z-scores at 36 weeks' PMA are undefined in infants who died before the corresponding time point, listwise deletion restricts this model to survivors; the primary analysis of this composite outcome, restricted to the covariates defined in every infant so that all 146 infants contribute, is reported in Figure 4 and Supplementary Table S11.

Dependent variable	Motor development at 24 months' CA and Death DoL 8-24 months' CA	B	S.E.	Wald	*p*-value	Exp(B)	95% C.I. for Exp(B)	
							Lower	Upper
Covariates	Antenatal corticosteroids[j], *number of doses*	-0.043	0.363	0.014	0.906	0.958	0.470	1.953
	Gestational age, *weeks*	0.118	0.176	0.444	0.505	1.125	0.796	1.590
	Invasive mechanical ventilation[l]	0.080	0.735	0.012	0.913	1.083	0.256	4.577
	Duration of fentanyl administration[n], *days*	0.030	0.085	0.123	0.726	1.030	0.872	1.218
	Duration of postnatal corticosteroid therapy[p], *days*	0.172	0.107	2.600	0.107	1.188	0.964	1.464
	Duration of antibiotic prophylaxis/therapy, *days*	0.071	0.078	0.824	0.364	1.073	0.921	1.250
	Duration of inotropic support[r], *days*	-0.356	0.292	1.489	0.222	0.700	0.395	1.241
	Intraventricular haemorrhage grade III-IV[t]	0.116	1.142	0.010	0.919	1.123	0.120	10.519
	Early-onset and/or late-onset sepsis	-3.458	1.793	3.718	0.054	0.032	0.001	1.059
	Retinopathy of prematurity (any stage)[u]	-0.491	0.783	0.393	0.531	0.612	0.132	2.840
	Anaemia requiring one or more red blood cell transfusions[y]	-0.850	0.735	1.336	0.248	0.427	0.101	1.806
	Hyperglycaemia during HS[z]	-0.080	0.938	0.007	0.932	0.923	0.147	5.802
	Metabolic acidosis during HS[)]	0.575	0.834	0.476	0.490	1.778	0.347	9.116
	Maternal milk intake DoL 0-7, *ml/kg/first week of life*	0.001	0.003	0.089	0.765	1.001	0.996	1.006
	Total milk intake DoL 0-7, *ml/kg/first week of life*	0.000	0.002	0.000	0.991	1.000	0.996	1.004
	Duration of parenteral nutrition, *days*	0.014	0.043	0.108	0.743	1.014	0.933	1.102

	Postnatal weight loss, *%*	0.050	0.066	0.581	0.446	1.052	0.924	1.197
	Head circumference at 36 weeks' PMA Z-Score[k]	0.047	0.318	0.022	0.883	1.048	0.562	1.953
	ΔZ-score head circumference 36 weeks' PMA-DoL 14[k,ψ]	0.256	0.338	0.574	0.449	1.292	0.666	2.504
	PMA at hospital discharge, *weeks*	-0.361	0.151	5.714	0.017*	0.697	0.519	0.937

For Legend, see Supplementary Table S1.

List of abbreviations: BW, birth weight; CA, corrected age; C.I., confidence interval; DoL, day of life; GA, gestational age; HS, hospital stay; PMA, postmenstrual age; S.E., standard error; Δ, variation.

Supplementary Table S15. Sensitivity analysis. Multivariate analysis, retaining the complete covariate set, of covariates influencing the composite outcome of pathological socio-emotional score at Bayley-III assessment and death between DoL 8 and 24 months' CA vs normal socio-emotional composite score at 24 months' CA in patients with GA at birth ≤ 32 weeks and/or BW ≤ 1500 g. [Nagelkerke R-squared = 0.172; Hosmer-Lemeshow test p-value = 0.400; Variance inflation factor (VIF) < 5 for all covariates]. Because the postmenstrual age at hospital discharge, the retinopathy status and the growth Z-scores at 36 weeks' PMA are undefined in infants who died before the corresponding time point, listwise deletion restricts this model to survivors; the primary analysis of this composite outcome, restricted to the covariates defined in every infant so that all 146 infants contribute, is reported in Figure 5 and Supplementary Table S11.

Dependent variable	Socio-emotional development at 24 months' CA and Death DoL 8-24 months' CA	B	S.E.	Wald	*p*-value	Exp(B)	95% C.I. for Exp(B)	
							Lower	Upper
Covariates	Maternal smoking during pregnancy	-0.848	0.487	3.032	0.082	0.428	0.165	1.112
	Male sex	-0.816	0.408	4.001	0.045*	0.442	0.199	0.984
	Gestational age, *weeks*	0.036	0.108	0.112	0.738	1.037	0.838	1.283
	Invasive mechanical ventilation[l]	0.302	0.532	0.322	0.570	1.353	0.476	3.839
	Duration of antibiotic prophylaxis/therapy, *days*	0.036	0.043	0.716	0.397	1.037	0.953	1.128
	Duration of inotropic support[r], *days*	0.028	0.112	0.062	0.804	1.028	0.826	1.280
	Early-onset and/or late-onset sepsis	-1.530	1.034	2.190	0.139	0.217	0.029	1.642
	Anaemia requiring one or more red blood cell transfusions[y]	0.131	0.544	0.058	0.809	1.140	0.393	3.310
	Hyperglycaemia during HS[z]	-0.222	0.700	0.100	0.751	0.801	0.203	3.159
	Metabolic acidosis during HS[)]	-0.443	0.628	0.499	0.480	0.642	0.188	2.196
	Total milk intake DoL 0-7, *ml/kg/first week of life*	0.000	0.001	0.018	0.894	1.000	0.998	1.003
	Duration of parenteral nutrition, *days*	-0.025	0.022	1.334	0.248	0.975	0.934	1.018
	ΔZ-score head circumference 36 weeks' PMA-DoL 14[k,ψ]	0.159	0.200	0.629	0.428	1.172	0.791	1.736

For Legend, see Supplementary Table S1.

List of abbreviations: BW, birth weight; CA, corrected age; C.I., confidence interval; DoL, day of life; GA, gestational age; HS, hospital stay; PMA, postmenstrual age; S.E., standard error; Δ, variation.

Supplementary Figure S1. Flexible calibration plots of the four primary composite models.

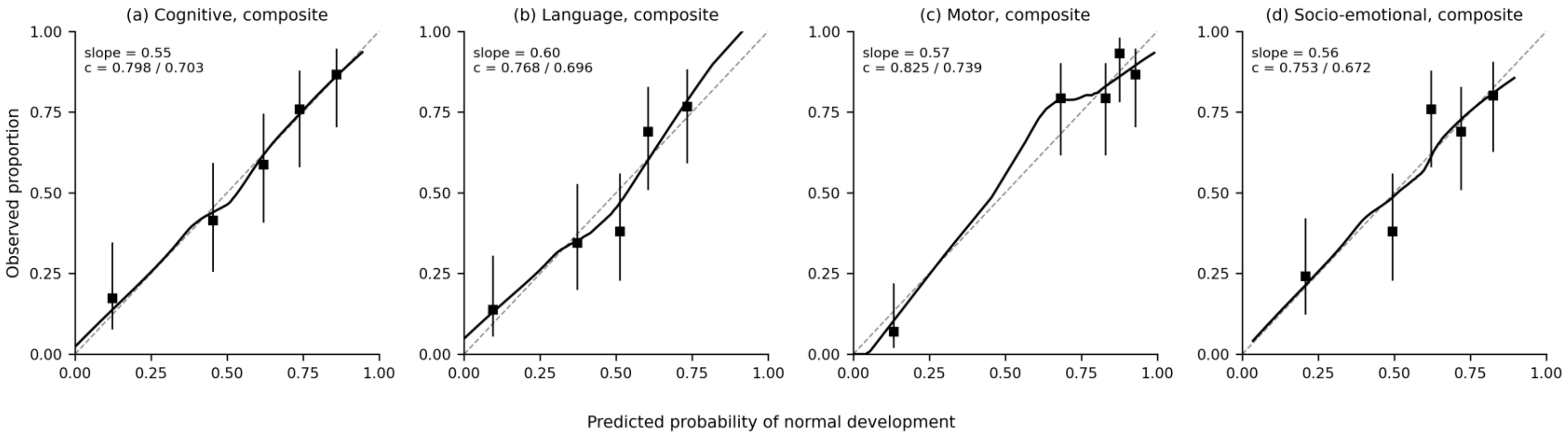

Legend: Observed proportion of infants with normal development plotted against the probability predicted by each of the four primary composite models of Figures 2–5, which are fitted on all 146 infants. Solid line, lowess smoothing of the observed outcome on the predicted probability; squares with bars, observed proportion with 95% confidence interval within quintiles of predicted risk, plotted against the mean predicted probability of the quintile; dashed line, ideal calibration. In each panel, slope is the bootstrap calibration slope and c the apparent and the optimism-corrected concordance statistic, both taken from Supplementary Tables S7 and S11. As in Supplementary Figure S3, the lowess curve and the quintile points describe apparent calibration in the sample in which each model was fitted and are therefore optimistic by construction, whereas the slope is the bootstrap-corrected estimate of the shrinkage that the coefficients require; the two are not expected to agree, and a curve lying close to the diagonal does not indicate that the model is well calibrated out of sample.

Supplementary Figure S2. Receiver operating characteristic curves of the four primary composite models.

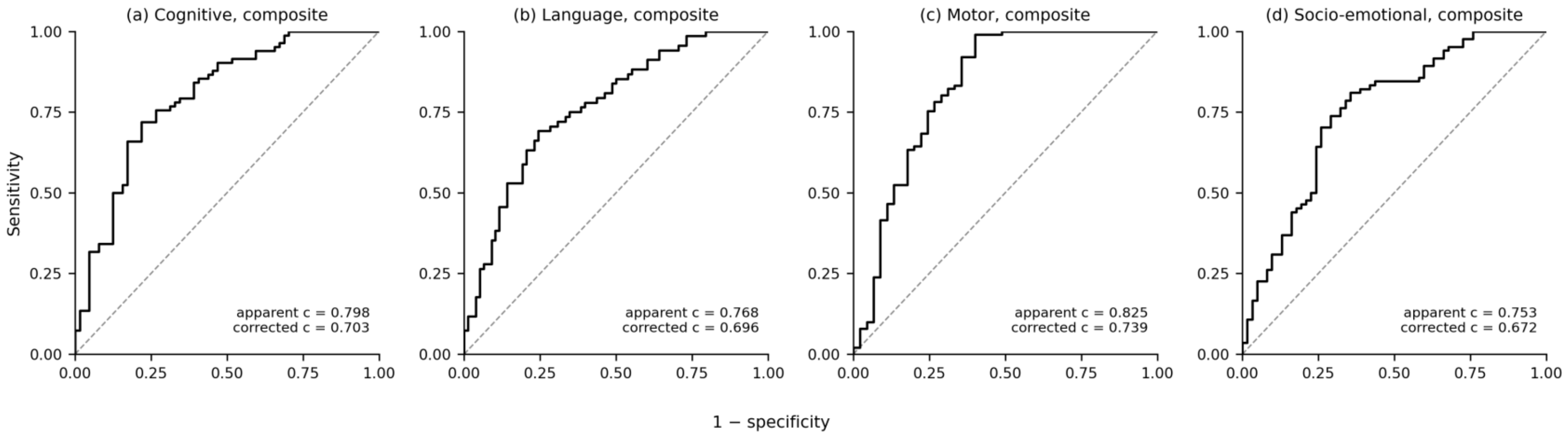

Legend: Solid line, receiver operating characteristic curve of the linear predictor of each of the four primary composite models of Figures 2–5, which are fitted on all 146 infants; dashed line, line of no discrimination. Apparent c is the concordance statistic in the sample in which the model was fitted; corrected c is the same statistic after bootstrap correction for optimism (Supplementary Tables S7 and S11).

Supplementary Table S16. Multivariate analysis of covariates influencing cognitive development at Bayley-III assessment (pathological vs normal) at 24 months' CA in patients with GA at birth ≤ 32 weeks and/or BW ≤ 1500 g. [Nagelkerke R-squared = 0.289; Hosmer-Lemeshow test *p*-value = 0.964; Variance inflation factor (VIF) < 5 for all covariates].

Dependent variable	Cognitive development at 24 months' CA	B	S.E.	Wald	*p*-value	Exp(B)	95% C.I. for Exp(B)	
							Lower	Upper
Covariates	Chorioamnionitis[g]	-1.648	0.768	4.609	0.032*	0.192	0.043	0.866
	Gestational age, *weeks*	-0.133	0.137	0.939	0.332	0.875	0.669	1.146
	5-minute Apgar score	0.362	0.204	3.145	0.076	1.435	0.963	2.140
	Duration of postnatal corticosteroid therapy[p], *days*	-0.024	0.076	0.098	0.754	0.977	0.842	1.132
	Duration of antibiotic prophylaxis/therapy, *days*	0.031	0.049	0.404	0.525	1.032	0.937	1.135
	Early-onset and/or late-onset sepsis	-1.934	1.141	2.873	0.090	0.145	0.015	1.353
	Retinopathy of prematurity (any stage)[u]	-1.101	0.653	2.842	0.092	0.333	0.092	1.196
	Bronchopulmonary dysplasia[w]	1.279	1.740	0.541	0.462	3.594	0.119	108.709
	Anaemia requiring one or more red blood cell transfusions[y]	0.232	0.650	0.127	0.721	1.261	0.353	4.505
	Hyperglycaemia during HS[z]	-0.769	0.799	0.926	0.336	0.463	0.097	2.219
	Maternal milk intake DoL 0-7, *ml/kg/first week of life*	0.003	0.002	2.800	0.094	1.003	0.999	1.007
	Total milk intake DoL 0-7, *ml/kg/first week of life*	-0.001	0.002	0.118	0.732	0.999	0.997	1.002
	Duration of parenteral nutrition, *days*	0.003	0.030	0.008	0.927	1.003	0.945	1.064
	ΔZ-score head circumference 36 weeks' PMA-DoL 14[k,ψ]	0.318	0.217	2.153	0.142	1.374	0.899	2.102
	PMA at hospital discharge, *weeks*	-0.130	0.127	1.044	0.307	0.878	0.685	1.127

For Legend, see Supplementary Table S1.

List of abbreviations: BW, birth weight; CA, corrected age; C.I., confidence interval; DoL, day of life; GA, gestational age; HS, hospital stay; PMA, postmenstrual age; S.E., standard error; Δ, variation.

Supplementary Table S17. Multivariate analysis of covariates influencing language development at Bayley-III assessment (pathological vs normal) at 24 months' CA in patients with GA at birth ≤ 32 weeks and/or BW ≤ 1500 g. [Nagelkerke R-squared = 0.255; Hosmer-Lemeshow test *p*-value = 0.868; Variance inflation factor (VIF) < 5 for all covariates].

Dependent variable	Language development at 24 months' CA	B	S.E.	Wald	*p*-value	Exp(B)	95% C.I. for Exp(B)	
							Lower	Upper
Covariates	Chorioamnionitis[g]	-1.486	0.775	3.679	0.055	0.226	0.050	1.033
	Male sex	-0.498	0.403	1.528	0.216	0.608	0.276	1.339
	Gestational age, *weeks*	0.023	0.116	0.041	0.840	1.024	0.815	1.286
	Duration of antibiotic prophylaxis/therapy, *days*	-0.043	0.042	1.058	0.304	0.958	0.883	1.039
	Intraventricular haemorrhage grade III-IV[t]	-2.250	1.133	3.948	0.047*	0.105	0.011	0.970
	Retinopathy of prematurity (any stage)[u]	-0.352	0.606	0.338	0.561	0.703	0.215	2.304
	Anaemia requiring one or more red blood cell transfusions[y]	0.458	0.634	0.521	0.470	1.580	0.456	5.476
	Hyperglycaemia during HS[z]	-0.739	0.801	0.851	0.356	0.478	0.099	2.296
	Total milk intake DoL 0-7, *ml/kg/first week of life*	0.001	0.001	1.493	0.222	1.001	0.999	1.004
	Duration of parenteral nutrition, *days*	0.007	0.027	0.062	0.804	1.007	0.955	1.062
	PMA at hospital discharge, *weeks*	-0.115	0.094	1.508	0.219	0.891	0.741	1.071

For Legend, see Supplementary Table S1.

List of abbreviations: BW, birth weight; CA, corrected age; C.I., confidence interval; DoL, day of life; GA, gestational age; HS, hospital stay; PMA, postmenstrual age; S.E., standard error.

Supplementary Table S18. Multivariate analysis of covariates influencing motor development at Bayley-III assessment (pathological vs normal) at 24 months' CA in patients with GA at birth ≤ 32 weeks and/or BW ≤ 1500 g. [Nagelkerke R-squared = 0.416; Hosmer-Lemeshow test *p*-value = 0.864; Variance inflation factor (VIF) < 5 for all covariates].

Dependent variable	Motor development at 24 months' CA	B	S.E.	Wald	*p*-value	Exp(B)	95% C.I. for Exp(B)	
							Lower	Upper
Covariates	Gestational age, *weeks*	0.034	0.168	0.042	0.839	1.035	0.745	1.438
	Invasive mechanical ventilation[l]	0.008	0.690	0.000	0.991	1.008	0.261	3.895
	Duration of antibiotic prophylaxis/therapy, *days*	0.103	0.073	1.966	0.161	1.108	0.960	1.280
	Duration of inotropic support[r], *days*	-0.100	0.244	0.170	0.680	0.904	0.561	1.459
	Early-onset and/or late-onset sepsis	-3.769	1.669	5.102	0.024*	0.023	0.001	0.607
	Retinopathy of prematurity (any stage)[u]	-0.511	0.748	0.466	0.495	0.600	0.139	2.599
	Anaemia requiring one or more red blood cell transfusions[y]	-0.655	0.714	0.844	0.358	0.519	0.128	2.102
	Hyperglycaemia during HS[z]	-0.243	0.869	0.078	0.780	0.784	0.143	4.304
	Metabolic acidosis during HS[)]	0.502	0.803	0.391	0.532	1.652	0.343	7.971
	Total milk intake DoL 0-7, *ml/kg/first week of life*	0.000	0.002	0.037	0.847	1.000	0.997	1.004
	Duration of parenteral nutrition, *days*	-0.004	0.039	0.011	0.915	0.996	0.923	1.074
	Postnatal weight loss, *%*	0.065	0.066	0.972	0.324	1.067	0.938	1.215
	Body weight at 36 weeks' PMA Z-Score[k]	0.186	0.340	0.300	0.584	1.205	0.619	2.347
	Head circumference at 36 weeks' PMA Z-Score[k]	-0.068	0.398	0.029	0.865	0.935	0.429	2.038
	ΔZ-score head circumference 36 weeks' PMA-DoL 14[k,ψ]	0.342	0.354	0.934	0.334	1.407	0.704	2.814
	PMA at hospital discharge, *weeks*	-0.228	0.128	3.151	0.076	0.796	0.619	1.024

For Legend, see Supplementary Table S1.

List of abbreviations: BW, birth weight; CA, corrected age; C.I., confidence interval; DoL, day of life; GA, gestational age; HS, hospital stay; PMA, postmenstrual age; S.E., standard error; Δ, variation.

Supplementary Table S19. Multivariate analysis of covariates influencing socio-emotional development at Bayley-III assessment (pathological vs normal) at 24 months' CA in patients with GA at birth ≤ 32 weeks and/or BW ≤ 1500 g. [Nagelkerke R-squared = 0.116; Hosmer-Lemeshow test *p*-value = 0.485; Variance inflation factor (VIF) < 5 for all covariates].

Dependent variable	Socio-emotional development at 24 months' CA	B	S.E.	Wald	*p*-value	Exp(B)	95% C.I. for Exp(B)	
							Lower	Upper
Covariates	Male sex	-0.894	0.381	5.503	0.019*	0.409	0.194	0.863
	Retinopathy of prematurity (any stage)[u]	-0.801	0.463	3.001	0.083	0.449	0.181	1.111
	Duration of parenteral nutrition, *days*	-0.024	0.015	2.426	0.119	0.976	0.947	1.006

For Legend, see Supplementary Table S1.

List of abbreviations: BW, birth weight; CA, corrected age; C.I., confidence interval; GA, gestational age; S.E., standard error.

Supplementary Figure S3. Flexible calibration plots of the eight multivariable binary logistic regression models of Supplementary Tables S12–S19.

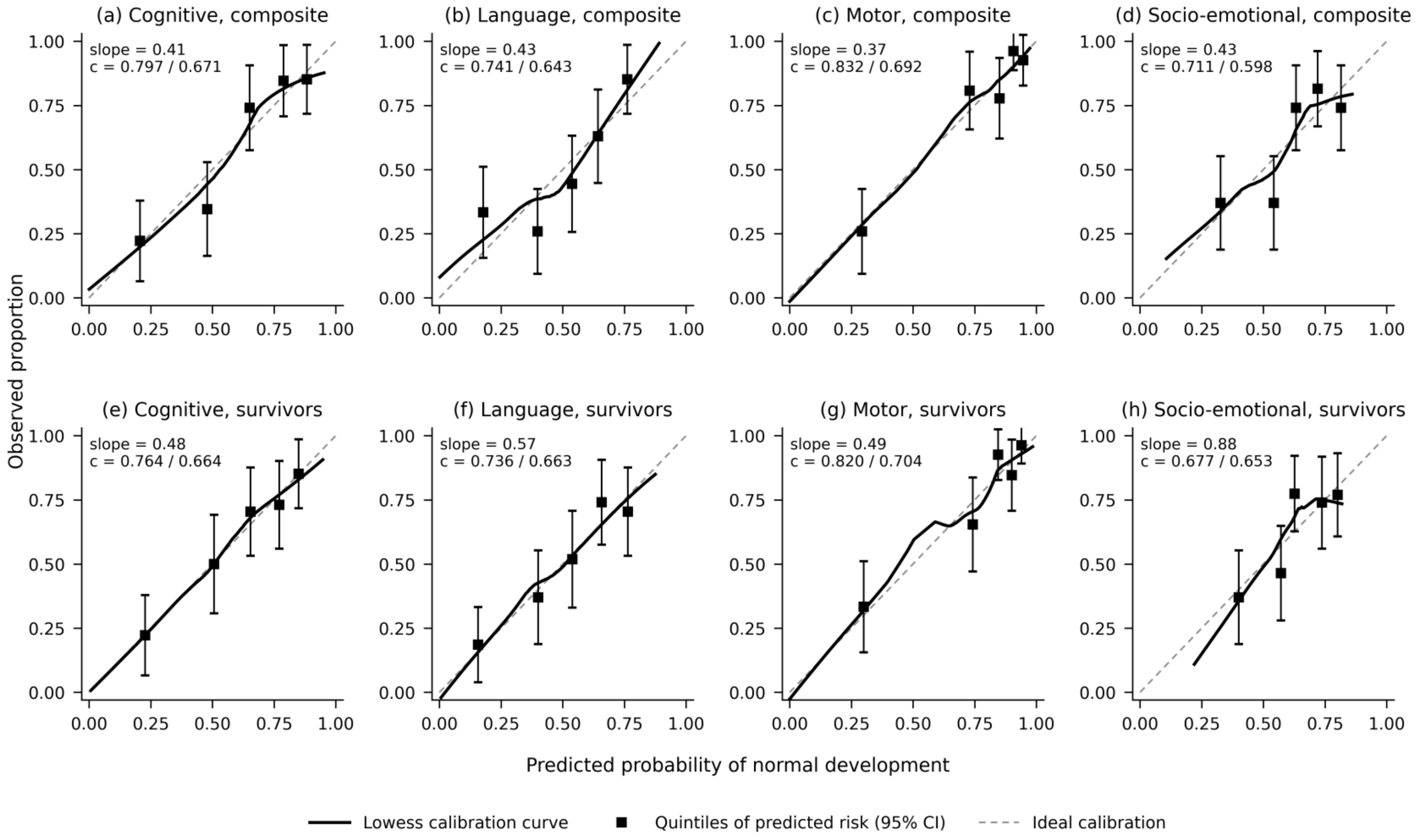

Legend: Observed proportion of infants with normal development plotted against the probability predicted by each model. Solid line, lowess smoothing of the observed outcome on the predicted probability; squares with bars, observed proportion with 95% confidence interval within quintiles of predicted risk, plotted against the mean predicted probability of the quintile; dashed line, ideal calibration. In each panel, slope is the bootstrap calibration slope and c the apparent and optimism-corrected concordance statistic, both taken from Supplementary Table S7. The lowess curve and the quintile points describe apparent calibration in the sample in which each model was fitted and are therefore optimistic by construction, whereas the slope is the bootstrap-corrected estimate of the shrinkage that the coefficients require; the two are not expected to agree, and a curve lying close to the diagonal does not indicate that the model is well calibrated out of sample. Panels (a) to (d) refer to the composite-outcome models of Supplementary Tables S12–S15, which retain the complete covariate set and which listwise deletion restricts to survivors, and are therefore the sensitivity analysis; panels (e) to (h) refer to the survivors-only (secondary) models of Supplementary Tables S16–S19. The corresponding plots for the four primary composite models, fitted on all 146 infants, are given in Supplementary Figures S1 and S2.

Supplementary Figure S4. Receiver operating characteristic curves of the eight multivariable binary logistic regression models of Supplementary Tables S12–S19.

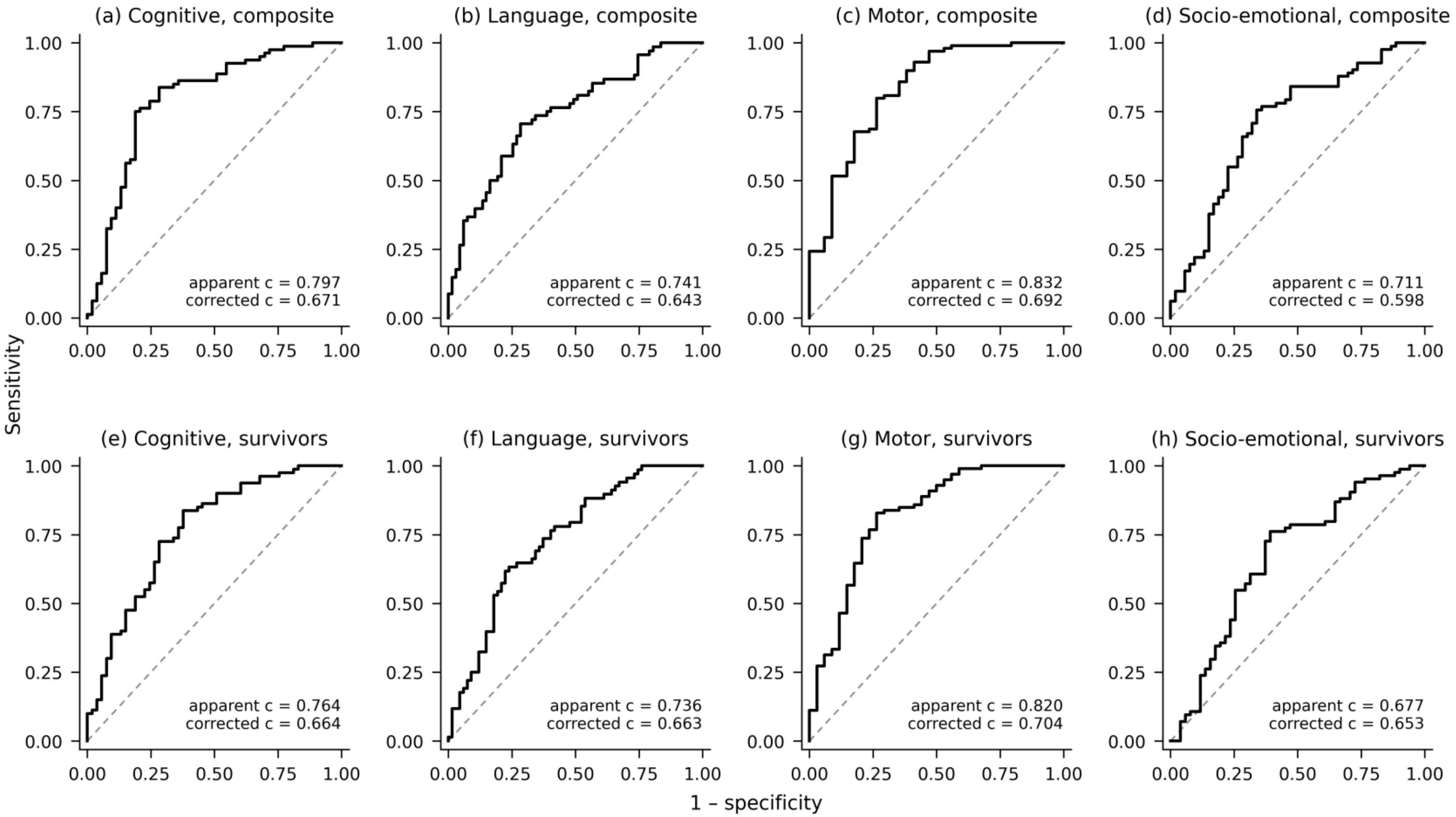

Legend: Solid line, receiver operating characteristic curve of the linear predictor of each model; dashed line, line of no discrimination. Apparent c is the concordance statistic in the sample in which the model was fitted; corrected c is the same statistic after bootstrap correction for optimism (Supplementary Table S7). Panels (a) to (d) refer to the composite-outcome models of Supplementary Tables S12–S15, which retain the complete covariate set and which listwise deletion restricts to survivors, and are therefore the sensitivity analysis; panels (e) to (h) refer to the survivors-only (secondary) models of Supplementary Tables S16–S19. The corresponding plots for the four primary composite models, fitted on all 146 infants, are given in Supplementary Figures S1 and S2.

Supplementary Table S20. Sensitivity analysis: the three models containing a covariate with a nominal departure from linearity refitted with that covariate entered as a restricted cubic spline.

**Model (outcome)**	**Covariate entered as a restricted cubic spline (p of the likelihood-ratio test for non-linearity)**	**Covariate significant at the nominal level in the linear model**	**Exp(B) (p), linear model**	**Exp(B) (p), spline model**	**Further covariates reaching p < 0.05 in the spline model**	**Nagelkerke R^2 and EPV, linear → spline**
S14 – Motor, composite outcome	Head circumference Z-score at 36 weeks' PMA (p = 0.005)	PMA at hospital discharge	0.697 (0.017)	0.618 (0.004)	Duration of postnatal corticosteroid therapy, Exp(B) 1.327 (p = 0.028); head circumference Z-score at 36 weeks' PMA, joint test on 2 degrees of freedom (p = 0.046)	0.439 → 0.499; 1.7 → 1.6
S17 – Language, survivors only	Total milk intake DoL 0–7 (p = 0.049); duration of parenteral nutrition (p = 0.042)	Intraventricular haemorrhage grade III–IV	0.105 (0.047)	0.105 (0.048)	None; neither spline term was significant on the joint test on 2 degrees of freedom (p = 0.19 for both)	0.255 → 0.312; 6.1 → 5.2
S18 – Motor, survivors only	Head circumference Z-score at 36 weeks' PMA (p = 0.035)	Early-onset and/or late-onset sepsis	0.023 (0.024)	0.025 (0.032)	None; the spline term was not significant on the joint test on 2 degrees of freedom (p = 0.145)	0.416 → 0.451; 2.1 → 2.0

Legend: For each of the 53 continuous covariate terms amenable to the test, linearity on the logit scale was examined by replacing the linear term with a restricted cubic spline with three knots placed at the 10th, 50th and 90th centiles of the covariate in the analytic sample of the model and comparing the two models with a likelihood-ratio test on one degree of freedom. The test reached the nominal threshold for four terms in three models; none of the four survived Benjamini-Hochberg control of the false discovery rate among the 53 tests (smallest adjusted value q = 0.25). The three models concerned were refitted with the covariate entered as a spline, all the other covariates being unchanged. In every case the covariate significant at the nominal level in the linear model remained significant, in the same direction and with a similar magnitude. In the motor composite model two further covariates reached the nominal threshold; this is the model with the least favourable ratio of events to variables of the eight (1.6 events per variable after the addition of the spline term), the departure from linearity on which it rests does not survive control of the false discovery rate, and the additional signals are therefore more plausibly attributable to overfitting than to a genuine non-linear effect. The linear specification was accordingly retained for the models presented in the manuscript. Exp(B) refers to the modelled event, which is normal neurodevelopment. Abbreviations: DoL, day of life; EPV, events per variable; PMA, postmenstrual age.

Supplementary Table S21. Missing data for the analysis variables.

Variable	Missing, n	Missing, %	Missing among the 11 deaths, n	Missing among the 135 survivors, n	Reason
Cognitive Bayley-III outcome, survivors only	11	7.5	11	0	Infant died before the assessment at 24 months' CA
Language Bayley-III outcome, survivors only	11	7.5	11	0	Infant died before the assessment at 24 months' CA
Motor Bayley-III outcome, survivors only	11	7.5	11	0	Infant died before the assessment at 24 months' CA
Socio-emotional Bayley-III outcome, survivors only	11	7.5	11	0	Infant died before the assessment at 24 months' CA
PMA at hospital discharge	11	7.5	11	0	Infant died before hospital discharge
ΔZ-score head circumference, 36 weeks' PMA – DoL 14	11	7.5	9	2	Infant died before 36 weeks' PMA (9); infant born at 35 and at 36 weeks' GA, so that the increment between DoL 14 and 36 weeks' PMA is undefined (2)
ΔZ-score body weight, 36 weeks' PMA – DoL 14	11	7.5	9	2	Infant died before 36 weeks' PMA (9); infant born at 35 and at 36 weeks' GA, so that the increment between DoL 14 and 36 weeks' PMA is undefined (2)
Maternal milk as a proportion of total enteral intake, DoL 0–7	10	6.8	4	6	Total enteral intake in the first week of life equal to zero, so that the proportion of maternal milk is undefined (6); infant died (4)
Head circumference Z-score at 36 weeks' PMA	9	6.2	9	0	Infant died before 36 weeks' PMA
Body weight Z-score at 36 weeks' PMA	9	6.2	9	0	Infant died before 36 weeks' PMA
Bronchopulmonary dysplasia	9	6.2	9	0	Infant died before 36 weeks' PMA, when the diagnosis is made
Congenital deafness (auditory brainstem response)	8	5.5	8	0	Infant died before 34 weeks' PMA, when screening is performed
Days to regain birth weight	6	4.1	6	0	Infant died; datum not recorded
Retinopathy of prematurity (any stage)	6	4.1	6	0	Infant died before 31 weeks' PMA, when screening starts
Head circumference Z-score at DoL 14	4	2.7	4	0	Infant died by DoL 14
Body weight Z-score at DoL 14	4	2.7	4	0	Infant died by DoL 14
Head circumference Z-score at birth	3	2.1	2	1	Infant born at 23 weeks' GA, below the 24 weeks + 0 days lower limit of validity of the INTERGROWTH-21st standards (1); infant died (2)

Variable	Missing, n	Missing, %	Missing among the 11 deaths, n	Missing among the 135 survivors, n	Reason
Birth weight Z-score	3	2.1	2	1	Infant born at 23 weeks' GA, below the 24 weeks + 0 days lower limit of validity of the INTERGROWTH-21st standards (1); infant died (2)
DoL of initiation of enteral feeding	1	0.7	1	0	Infant died; datum not recorded

Legend: The table lists all analysis variables with at least one missing value. Of the 85 variables analysed - the 81 candidate explanatory variables listed in Supplementary Table S10 and the four domain-specific outcome variables of the survivors-only analysis — 66 were complete and 19 are listed here; no variable exceeded 7.5% of missing values. Of the 149 missing values in total, 137 (91.9%) occurred in the 11 infants who died between DoL 8 and 24 months' CA. For the variables defined at 36 weeks' PMA, at the age of a scheduled screening or at hospital discharge, every missing value in an infant who died corresponds to a quantity that was not undetermined but undefined, the infant having died before the time at which the variable is measured; missingness in these variables is therefore deterministic given death rather than random. Multiple imputation was consequently not applied: imputing, for instance, a postmenstrual age at discharge or a 36-week growth Z-score for an infant who died beforehand would generate values without a referent rather than recover unobserved ones. Analyses were performed on available cases and the corresponding denominators are reported in Tables 1–4. For every variable ascertained at a defined postmenstrual age or day of life, the correspondence between the missing value and death before that time point was verified against the postmenstrual age at death of each of the 11 infants who died. Of the 12 missing values that occurred in survivors, all correspond to quantities that are likewise undefined rather than unobserved: six to a proportion of maternal milk whose denominator (total enteral intake in the first week of life) is zero, four to the changes in Z-score of body weight and of head circumference between DoL 14 and 36 weeks' PMA in the two infants born at 35 and 36 weeks' GA, and two to the Z-scores at birth of an infant born at 23 weeks' GA, the INTERGROWTH-21st standards being applicable from 24 weeks + 0 days onwards. Missingness in this database is therefore deterministic rather than random, with the exception of the seven values noted below, and this is the reason why multiple imputation was not applied. More precisely, missingness is entirely predictable from death, which is itself observed, so that the situation is one of truncation by death rather than of missing data in the usual sense: the quantity does not exist for the infant concerned and there is therefore nothing for an imputation model to recover. Seven of the 149 missing values are an exception, being quantities that existed but were not recorded, namely the day of initiation of enteral feeding in one infant and the day of recovery of birth weight in six infants who died; given their number these were also analysed on available cases.

List of abbreviations: CA, corrected age; DoL, day of life; PMA, postmenstrual age.

Supplementary Figure S5. Multivariable analysis of the secondary (survivors-only) outcome - cognitive domain (pathological versus normal cognitive Bayley-III score at 24 months' corrected age).

Legend: Data are adjusted odds ratios [Exp(B)] with 95% confidence intervals from a multivariable binary logistic regression model restricted to survivors (n = 133). The dependent variable was coded so that the modelled event was normal development; therefore Exp(B) > 1 indicates higher odds of a normal outcome and Exp(B) < 1 higher odds of a pathological outcome. Dashed line, reference value (odds ratio = 1); arrowheads, confidence limits extending beyond the axis range. * $p < 0.05$. Model fit: Nagelkerke $R^2 = 0.289$; Hosmer-Lemeshow $p = 0.964$; variance inflation factor < 5 for all covariates. **Abbreviations:** CI, confidence interval; DoL, day of life; PMA, postmenstrual age; RBC, red blood cell; Δ, variation.

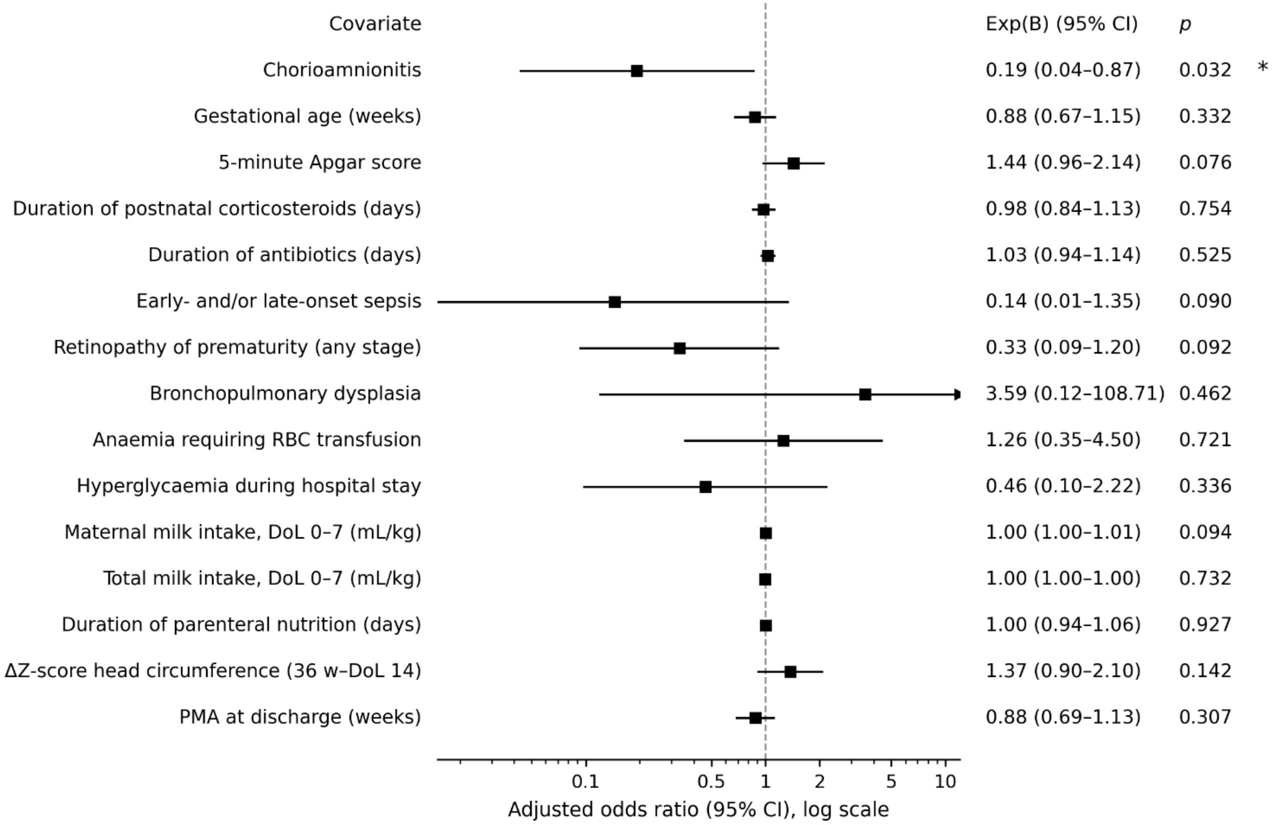

Supplementary Figure S6. Multivariable analysis of the secondary (survivors-only) outcome - language domain (pathological versus normal language Bayley-III score at 24 months' corrected age).

Legend: Data are adjusted odds ratios [Exp(B)] with 95% confidence intervals from a multivariable binary logistic regression model restricted to survivors (n = 135). The dependent variable was coded so that the modelled event was normal development; therefore Exp(B) > 1 indicates higher odds of a normal outcome and Exp(B) < 1 higher odds of a pathological outcome. Dashed line, reference value (odds ratio = 1); arrowheads, confidence limits extending beyond the axis range. * $p < 0.05$. Model fit: Nagelkerke $R^2 = 0.255$; Hosmer-Lemeshow $p = 0.868$; variance inflation factor < 5 for all covariates. **Abbreviations:** CI, confidence interval; DoL, day of life; PMA, postmenstrual age; RBC, red blood cell.

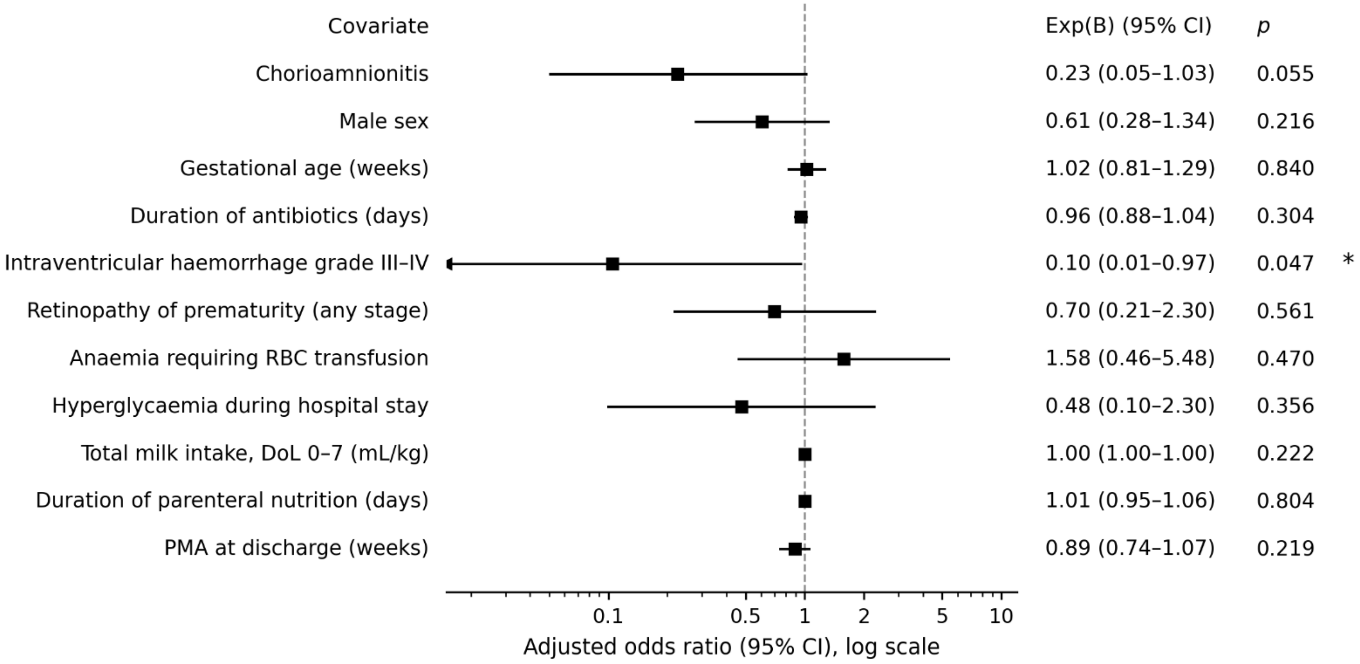

Supplementary Figure S7. Multivariable analysis of the secondary (survivors-only) outcome - motor domain (pathological versus normal motor Bayley-III score at 24 months' corrected age).

Legend: Data are adjusted odds ratios [Exp(B)] with 95% confidence intervals from a multivariable binary logistic regression model restricted to survivors (n = 133). The dependent variable was coded so that the modelled event was normal development; therefore Exp(B) > 1 indicates higher odds of a normal outcome and Exp(B) < 1 higher odds of a pathological outcome. Dashed line, reference value (odds ratio = 1); arrowheads, confidence limits extending beyond the axis range. * $p < 0.05$. Model fit: Nagelkerke $R^2 = 0.416$; Hosmer-Lemeshow $p = 0.864$; variance inflation factor < 5 for all covariates. **Abbreviations:** CI, confidence interval; DoL, day of life; PMA, postmenstrual age; RBC, red blood cell; Δ, variation.

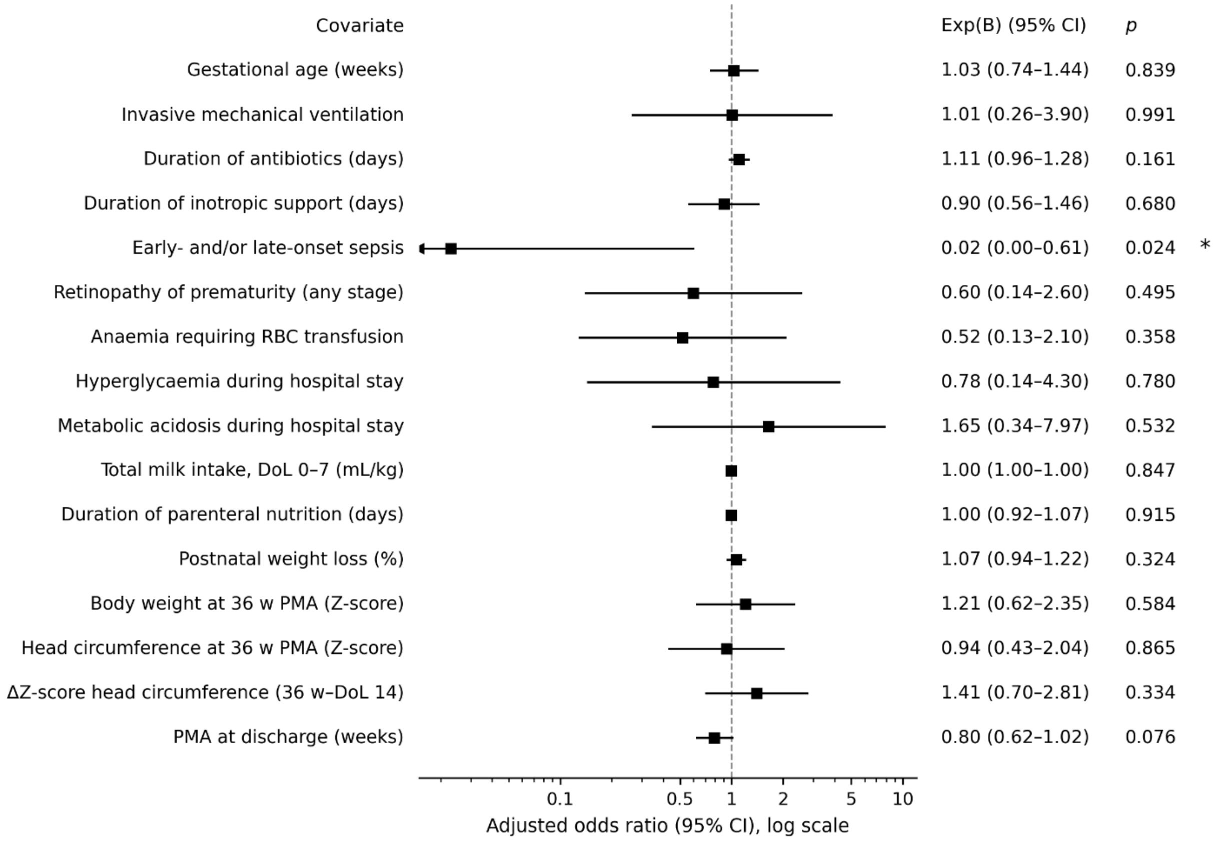

Supplementary Figure S8. Multivariable analysis of the secondary (survivors-only) outcome - socio-emotional domain (pathological versus normal socio-emotional Bayley-III score at 24 months' corrected age).

Legend: Data are adjusted odds ratios [Exp(B)] with 95% confidence intervals from a multivariable binary logistic regression model restricted to survivors (n = 135). The dependent variable was coded so that the modelled event was normal development; therefore Exp(B) > 1 indicates higher odds of a normal outcome and Exp(B) < 1 higher odds of a pathological outcome. Dashed line, reference value (odds ratio = 1); arrowheads, confidence limits extending beyond the axis range. * $p < 0.05$. Model fit: Nagelkerke $R^2 = 0.116$; Hosmer-Lemeshow $p = 0.485$; variance inflation factor < 5 for all covariates. **Abbreviations:** CI, confidence interval.

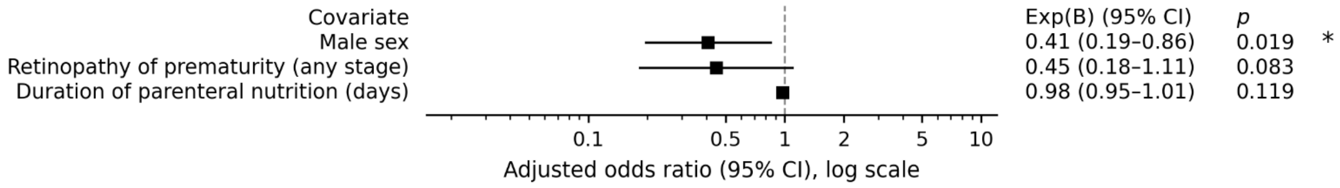

Supplementary Table S22. Covariates significant at the nominal level in the twelve multivariable models (abridged version of Supplementary Tables S12–S19 and S11).

Model	Covariate	B	S.E.	Wald	p-value	Exp(B)	95% C.I. for Exp(B)
**Primary – Language, composite outcome**	Intraventricular haemorrhage grade III–IV	-2.782	1.126	6.107	0.013*	0.062	0.007–0.562
**Primary – Motor, composite outcome**	Early-onset and/or late-onset sepsis	-3.999	1.549	6.664	0.010*	0.018	0.001–0.382
**Primary – Motor, composite outcome**	Anaemia requiring one or more RBC transfusions	-1.444	0.657	4.829	0.028*	0.236	0.065–0.856
**Primary – Socio-emotional, composite outcome**	Maternal smoking during pregnancy	-0.963	0.482	3.986	0.046*	0.382	0.148–0.983
**Primary – Socio-emotional, composite outcome**	Male sex	-0.882	0.400	4.877	0.027*	0.414	0.189–0.906
**Primary – Socio-emotional, composite outcome**	Early-onset and/or late-onset sepsis	-2.117	0.956	4.908	0.027*	0.120	0.019–0.783
**S12 – Cognitive, composite outcome**	Maternal high school diploma or higher educational level	1.092	0.545	4.008	0.045*	2.980	1.023–8.680
	Retinopathy of prematurity (any stage)	-1.393	0.698	3.980	0.046*	0.248	0.063–0.976
**S13 – Language, composite outcome**	Intraventricular haemorrhage grade III–IV	-2.675	1.190	5.050	0.025*	0.069	0.007–0.710
**S14 – Motor, composite outcome**	PMA at hospital discharge	-0.361	0.151	5.714	0.017*	0.697	0.519–0.937
**S15 – Socio-emotional, composite outcome**	Male sex	-0.816	0.408	4.001	0.045*	0.442	0.199–0.984
**S16 – Cognitive, survivors only**	Chorioamnionitis	-1.648	0.768	4.609	0.032*	0.192	0.043–0.866
**S17 – Language, survivors only**	Intraventricular haemorrhage grade III–IV	-2.250	1.133	3.948	0.047*	0.105	0.011–0.970
**S18 – Motor, survivors only**	Early-onset and/or late-onset sepsis	-3.769	1.669	5.102	0.024*	0.023	0.001–0.607
**S19 – Socio-emotional, survivors only**	Male sex	-0.894	0.381	5.503	0.019*	0.409	0.194–0.863

Legend: Abridged version of Supplementary Tables S12–S19 and S11, restricted to the covariates that reached the nominal significance threshold ($p < 0.05$) in each of the twelve multivariable binary logistic regression models. The rows labelled Primary belong to the four primary composite models displayed in Figures 2–5 and reported in full in Supplementary Table S11; no covariate reached the nominal threshold in the primary model of the cognitive domain, which is therefore absent from this table. It is provided, as suggested during peer review, as a rapid-reading counterpart to the complete models, which are retained unabridged in Supplementary Tables S12–S19 and S11 because the simultaneous adjustment for the whole covariate set is what the estimates reported here are conditional on: reading a coefficient without the covariates alongside which it was estimated would misrepresent it. In every model the dependent variable was coded so that the modelled event was normal neurodevelopment, so that Exp(B) > 1 indicates higher odds of a normal outcome and Exp(B) < 1 higher odds of the adverse or composite outcome. None of these covariates remained significant after control of the false discovery rate (Supplementary Table S9).

List of abbreviations: C.I., confidence interval; PMA, postmenstrual age; S.E., standard error.
